# Targeting autophagy peptidase ATG4B with a novel natural product inhibitor Azalomycin F4a for advanced gastric cancer

**DOI:** 10.1038/s41419-022-04608-z

**Published:** 2022-02-18

**Authors:** Lin Zhong, Bin Yang, Zhenhua Zhang, Junfeng Wang, Xiaojuan Wang, Yinfeng Guo, Weifeng Huang, Qianqian Wang, Guodi Cai, Fan Xia, Shengning Zhou, Shuai Ma, Yichu Nie, Jinping Lei, Min Li, Peiqing Liu, Wenbin Deng, Yonghong Liu, Fanghai Han, Junjian Wang

**Affiliations:** 1 Department of Gastrointestinal Surgery, Sun Yat-sen Memorial Hospital, Sun Yat-sen University, 510120 Guangzhou, Guangdong China; 2 Department of Pharmaceutical Sciences (Shenzhen), Sun Yat-Sen University, 510006 Guangzhou, Guangdong China; 3 CAS Key Laboratory of Tropical Marine Bio-resources and Ecology/Guangdong Key Laboratory of Marine Materia Medica, South China Sea Institute of Oceanology, Chinese Academy of Sciences, 510301 Guangzhou, China; 4 Hepatopancreatobiliary Center, Beijing Tsinghua Changgung Hospital, Tsinghua University, No.168, Litang Road, Changping District, 102218 Beijing, China; 5 School of Pharmaceutical Sciences, Sun Yat-sen University, 510006 Guangzhou, Guangdong China; 6 Guangdong Provincial Key Laboratory of New Drug Design and Evaluation, National-Local Joint Engineering Laboratory of Druggability and New Drugs Evaluation, Sun Yat-sen University, 510006 Guangzhou, Guangdong China

**Keywords:** Drug development, Gastric cancer

## Abstract

Advanced gastric cancer (GCa) remains highly lethal due to the lack of effective therapies. Identifying promising therapeutic targets and developing effective treatment against GCa are urgently needed. Through mRNA and protein analysis of GCa clinical tumor samples, we found that autophagy-related gene 4B (ATG4B) was overexpressed in GCa tumors and that its high expression was associated with patients’ poor prognosis. Knockdown of ATG4B significantly inhibited GCa cell survival and tumor growth. To further probe the role of ATG4B in GCa by pharmacological means, we screened an in-house marine natural compound library against ATG4B and identified Azalomycin F4a (Am-F4a) as a novel and potent ATG4B inhibitor. Am-F4a directly bound to ATG4B with high affinity and effectively suppressed GCa cell autophagy via inhibition of ATG4B both in vitro and in vivo. Moreover, Am-F4a or ATG4B knockdown significantly suppressed tumor growth as well as GCa cell migration and invasion. Am-F4a effectively blocked the metastatic progression of primary GCa and sensitized tumors to chemotherapy. Taken together, our findings indicate that ATG4B is a potential therapeutic target against GCa and the natural product Am-F4a is a novel ATG4B inhibitor that can be further developed for the treatment of GCa.

## Introduction

Gastric cancer (GCa) is an aggressive cancer and the fourth leading cause of cancer-related death worldwide [1]. It is characterized by rapid cancer progression and widespread metastasis. Approximately 60% of GC patients are initially diagnosed with local or distant metastasis [2], which accounts for about 90% cancer-associated death [3, 4]. Over the past two decades, the development of surgical operation and neoadjuvant chemotherapy (NACT) have significantly improved the 5-year overall survival rate of patients with localized GCa (>60%), whereas the 5-year overall survival rates of GCa patients with local and distant metastasis are approximately 30% and 5%, respectively [5–7]. So far, chemotherapy remains the backbone of advanced GCa treatment, although it only modestly improves patient’s survival [8]. Recently, targeted therapies in GCa have made promising progress. Trastuzumab, a HER2 monoclonal antibody, was approved for advanced HER2-positive GCa treatment and successfully improved patient’s survival [7, 9–11]. In addition, antibodies against VEGFR-2 (ramucirumab) and PD-1 (nivolumab or pembrolizumab), as second-line and third-line treatments, also improved overall survival of patients with advanced GCa [7, 12]. However, options of targeted therapy for GCa are still limited and they only benefit a small fraction of patients, therefore, identifying novel antitumor targets and developing effective drugs for advanced GCa is urgently needed.

Autophagy-related gene 4 (ATG4) is a cysteine protease required for autophagosome formation and consists of four homologues—ATG4A, ATG4B, ATG4C, and ATG4D [13]. ATG4B shows high proteolytic activity on autophagy marker Atg8 orthologs (GATE-16, GABARAP, LC3, and Apg8L) [14, 15], while ATG4A only works on GABARAP subfamily proteins and ATG4C/D are almost inactive [16, 17]. In ATG4B knockout mice, ATG4B depleted tissues exhibit notable defects in LC3-II conversion, autophagic flux, and autophagosome formation [18, 19]. Dysregulated autophagy tightly correlates with tumorigenesis and cancer progression. Accumulating studies suggest that ATG4B plays oncogenic roles in a number of cancers, such as colorectal cancer and glioblastoma (GBM) [20]. ATG4B is overexpressed in tumor cells of colorectal patients. Pharmacological and genetic inhibition of ATG4B inhibited colorectal cancer cell proliferation and xenograft tumor growth [14]. In GBM cells, ATG4B inhibition significantly suppressed cellular autophagic activity and tumorigenicity, as well as enhanced the anti-GBM efficacy of radiotherapy [21]. In addition, ATG4B overexpression conferred cancer drug resistance while its inhibition markedly sensitized tumors to chemotherapy in lung cancer [22], colon cancer [23], and chronic myeloid leukemia [24]. These studies indicate that ATG4B is a potential anticancer target. However, the function of ATG4B in GCa is unclear.

Although several ATG4B inhibitors (S130, NSC185058, Tioconazole, et al.) have been identified [14, 25, 26], their activity and selectivity are limited. The development of novel and potent ATG4B inhibitors with different scaffolds are still needed to explore the therapeutic potential of targeting ATG4B in cancer. Natural products exhibit high diversity in chemical structures and are one major source of drug development [27]. More than 40% of the approved anticancer drugs, including first-line chemotherapy drug paclitaxel, etoposide, and irinotecan, were either natural products or derived from natural products [27, 28]. Recently, drug development from marine natural products gained much attention. There are remarkably high hit rates from marine source, due to their unique and diverse molecular structures [29]. Several marine-derived compounds include Adcetris^®^ [30], Halaven^®^ [31], Yondelis® [32], and Cytosar-u^®^ [33] have already been approved for cancer therapy. In addition, new technologies have markedly accelerated the discovery of novel marine natural products and drug candidates.

In this study, we found that high ATG4B expression correlated with poor survival of patients with GCa and was essential for tumor growth. We also discovered a novel ATG4B inhibitor Am-F4a from a marine natural product library. The compound could effectively suppress GCa cell growth both in vitro and in vivo. Furthermore, ATG4B inhibition significantly blocked the progression of GCa metastasis. Therefore, our results suggest that targeting ATG4B with the novel inhibitor Am-F4a might be a new approach to treat advanced GCa.

## Materials and methods

### Cell culture and reagents

The AGS, MKN45, HGC27, SNU1, KATOIII, Hela and HEK293T cells were obtained from the American Type Culture Collection (ATCC). Human GCa cell lines MGC803 were purchased from China Academia Sinica (Shanghai, PR China). The GCa MGC803, AGS, MKN45, HGC27, SNU1, KATOIII cells were cultured in RPMI-1640 medium and Hela cells, HEK293T embryonic kidney cells were cultured in DMEM medium, supplemented with 10% fetal bovine serum and 1% penicillin/streptomycin in a humidified incubator at 37 °C with 5% CO_2_. Antibodies against the following proteins were used with source and dilution ratios indicated: ATG4B (Cell signaling, #13507, 1:1000 and Proteintech, # Cat No. 15131-1-AP, 1:1000); P62 (Sigma, #P0067, 1:1000); LC3 (Sigma, #ABC929, 1:1000; immunofluorescence 1:100); LAMP1(CST, #15665S, 1:100); Snail (CST, #4719, 1:1000); N-cadherin (CST, #4061, 1:1000); C-caspase7 (CST, #12827, 1:1000); PARP-1 (CST, #9542, 1:1000); GAPDH (CST, #2118, 1:1000); Anti-rabbit IgG Fab2 (CST, #4412s, 1:500); Anti-mouse IgG Fab2 (CST, #4409s, 1:500). AzalomycinF4a was isolated from *Streptomyces solisilvae* HNM30702 and verified by the NMR and HRESIMS data [34]. Rapamycin (MCE, #HY-10219); BafilomycinA1 (MCE, # HY-100558); Tioconazole (MCE, #HY-1303191 CS-2360); Acridine Orange (Sigma, #MKCD9806); FMK9a (MCE, HY-100522); DAPI (Beyotime, ON.C1005).

### Immunohistochemistry

Immunohistochemistry (IHC) was performed in 30 GCa and five normal tissues. Immunohistochemical detection was performed using the Universal three-step detection kit (SP-0022, China). Slices were treated with EDTA buffer for antigen retrieval at 100 °C temperature for 20 min, incubated with 3% H_2_O_2_ for 10 min at room temperature to inactivate endogenous peroxidase activity. ATG4B antibody was diluted at 1:200 and incubated for 12 h at 4 °C. DAB was used as a chromogenic substrate. A negative control was also established using the same experimental conditions. Three fields with ×200 magnification were randomly selected, and the immunoreactivity score was performed to calculate the results by Image J (IHC Profiler), Score = (Number of pixelsina zone) × (Score of the zone)/Total number of pixels in the image. The score is divided into 4 grades, as previous report [35]. High Positive (Score = 4), Positive (Score = 3), Low Positive (Score = 2), Negative (Score = 1).

### Analysis of ATG4B expression and Kaplan–Meier survival curve in clinical tumors

Publicly available GCa expression datasets GSE13911 and GSE19826 were downloaded from GEO at http://www.ncbi.nlm.nih.gov/geo/. The datasets contain gene expression profiles of normal and GCa tumor samples. The expression of ATG4B in different groups was analyzed as described previously. For Kaplan–Meier survival curve analysis, patients with GCa tumors were stratified by the ATG4B transcript levels, the overall survival and relapse-free survival of patients were analyzed using an online survival analysis tool (http://kmplot.com/), statistical significance was assessed by the log-rank test.

### Western blotting, siRNA transfection, and shRNA lentivirus transduction

Western blotting was performed with the indicated primary antibodies as our previous reports [36]. For siRNA transfection, siRNAs were obtained from Sangon Biotech (shanghai) Co., Ltd. The siRNA sequences for ATG4B were as follows: siATG4B#1, GAAAGAUUCGACUCAGAATT; siATG4B#2, GGUGUGGACAGAUGAUCUUUGTT; siControl, CAGUCGCGUUUGCGACUGG. For shRNA assay, the ATG4B shRNA sequence was as follows: ATG4B: 5′-TGATGTGGCATCTAGACTTTG-3′; it was purchased from Sangon Biotech (shanghai) Co., Ltd and cloned into a PLKO.1 lentivirus vector. ATG4B and control shRNA Lentiviral particles were produced in 293T cells after co-transfected with the lentivirus vectors, packaging plasmid psPAX2 and envelope plasmid pMD2.G. siRNA transfections and shRNA lentivirus transduction were performed as described previously.

### Cell growth and colony formation

For cell growth, GCa cells were seeded in 6 well plates at 1 × 10^6^ per well in triplicate. After indicated 48 h of drug treatment, total viable cell numbers were counted by a cell counter. In addition, the cell viability was also determined by CCK-8 assay (kit. 40203ES60*, Yeasen, China). Briefly, cells were seeded in 96-well plates at 800 cells per well, after 12 h, different doses of compounds were added to cell culture medium and cells were cultured for another 96 h. Cell viability was measured by a CCK-8 kit according to the manufacturer’s instruction. For colony formation, GCa cells were seeded in 6-well plates at 800 per well and cultured for 7–12 days, cell culture medium containing indicated compounds were replaced every 3 days. Cells were then fixed in 4% paraformaldehyde for 15 min, and then washed with PBS three times. Cell colonies were stained with crystal violet for 30 min and washed with PBS six times. Colonies consisted of more than 50 cells were counted and graphed.

### Flow cytometry

Cell apoptosis was detected by flow cytometry using an Annexin V-FITC Apoptosis Detection Kit I (BestBio) following the manufacturer’s protocols. Briefly, 1 × 10^5^ per well AGS and MGC803 cells were seeded in the six-well plates. Then, cells were treated with Am-F4a at indicated concentrations or transfected with siRNA for 48 h. Cells were harvested with trypsin, washed twice with cold PBS, resuspended with 400 μL binding buffer, incubated with 2.5 μL Annexin V-FITC for 15 min and 5 μL propidium iodide for 5 min at 4 °C. Finally, cells were detected by CytoFLEX S and data were analyzed with FlowJo Version 10.0 software.

### Migration and invasion assays

The migration of GCa cells was determined using wound healing assay. Briefly, when cells in 6-well plates were about 100% confluent, cell monolayer was scratched using pipette tips. Cell culture medium were replaced and drugs were added. After indicated time, the images of the wound were photographed by a microscope and wound closure rates were calculated. Cell invasion assay were performed using transwell chamber (Costar, USA). Upper insert of chambers was plated with 100 μL Matrigel per well and kept in 37 °C incubator for 4 h. 300 μL serum-free RPMI-1640 medium containing GC cells (1–2 × 10^5^) were added into the upper insert, 500 μL RPMI-1640 containing 5% FBS was added to the bottom chamber. After cells were treated with indicated drugs for 24 h, invading cells were fixed with 4% paraformaldehyde and stained with crystal violet staining dilution. Cells were counted in five randomly microscope field each well and experiments were conducted in triplicate.

### Molecular docking

The crystal structure of human ATG4B (PDB code: 2CY7.pdb) downloaded from Protein Data Bank (http://www.pdb.org) was used for the molecular docking. The molecular docking was performed by Schrodinger program following our previous study [37]. The ligand and protein structure preparation, including water deletion, protonation-state adjustment, and hydrogen atoms and disulfide bonds adding were performed by Maestro (version 11.6.013, Schrödinger, LLC, New York, NY, 2018). The Glide docking program in Maestro 11.6.013 was used for docking studies. The designed molecule Am-F4a was docked into ATG4B using Glide SP mode, the grid was defined using a 45 Å box centered on the CG atom of residue LEU11 of ATG4B. All other parameters were kept as default. The PyMOL software (DeLano Scientific, Palo Alto, CA, USA) was used to obtain the 3D structure of the docking model.

### FRET assay and surface plasmon resonance (SPR) analysis

The inhibition of compounds on ATG4B activity was performed as our previous report [36]. The percentage of inhibition was used to plot drug concentration-response curve and calculate IC50 values (Graphpad 8.0, GraphPad Software, La Jolla, CA, USA). The binding affinity of Am-F4a and ATG4B protein was measured by SPR assay on a Biacore 8 K instrument (GE Healthcare, Piscataway, NJ, USA). Briefly, purified ATG4B protein (200 μg/mL, pH 8.0) were immobilized (~10,000RU) on a Series S Sensor Chip (GE Healthcare, Piscataway, NJ, USA) according to a standard amine coupling procedure. Running buffer for immobilization was PBS (Servicebio, G0002, pH7.2-7.4) containing 5% DMSO. After immobilization, compound Am-F4a serially diluted in running buffer was as stock solution. Seven concentrations of Am-F4a (20, 10, 5, 2.5, 1.25, 0.625, 0 μM) were simultaneously injected at a flow rate of 65 μL/min for 60 s of association phase at 25 °C. Biacore 8K manager software was used to calculate the equilibrium dissociation constant (*K*_d_).

### mRFP-eGFP-LC3 translocation and lysosomal function analysis

Hela cells were transfected with the plasmid encoding mRFP-eGFP-LC3 as described previously [14]. After 24 h, Hela cells expressing mRFP-eGFP-LC3 were treated with compounds at the indicated concentrations. For lysosomal function analysis, cells were treated with Am-F4a (10 μM), Rap (1 μM), Baf (0.5 μM) for 4 h, followed by staining with Acridine Orange (AO, 0.5 μg/mL) for 30 min. Fluorescence images of live cells were taken by confocal microscopy (Olympus Corporation, Japan).

### Immunofluorescence of LC3 and LAMP1

Hela cells were treated with Am-F4a (10 μM), Rap (1 μM) for 6 h. After fixation by 4% paraformaldehyde for 15 min and then incubated for another 15 min with 0.5% Triton X-100. Incubation with 1% BSA for 1 h. Then cells were incubated with a combination of antibodies overnight at 4 °C. The combination of antibodies used was LC3 rabbit polyclonal (1:100) and LAMP1 mouse polyclonal (1:100). Incubated with a combination of secondary antibodies for 1 h. The combination of secondary antibodies used was Anti-mouse IgG Fab2 Alexa Fluor (R) 488 (1:500) and Anti-rabbit IgG Fab2 Alexa Fluor (R) 555 (1:500). The cells were then counterstained with DAPI (Beyotime, ON.C1005, China) for 3 min. Fluorescence images were taken by confocal microscopy (Olympus Corporation, Japan) with ×60 objective.

### Transmission electron microscopy (TEM)

MGC803 cells in 6-well plates were fixed in 2.5% glutaraldehyde for 2 h, and then dehydrated in a graded ethanol series and embedded. Ultrathin sections were mounted and post-stained with 2% uranyl acetate followed by 0.3% lead citrate. Sections were imaged using a transmission electron microscope G2 20 Twin (FEI, USA).

### Patients and specimens

The GCa and normal tissues of patients were obtained from Sun Yat-sen Memorial Hospital of Sun Yat-sen University, Guangzhou, China. All the specimens were confirmed by pathological examination. Informed consent was obtained from all patients. All clinical studies were approved by the Clinical Research Ethics Committee of Sun Yat-sen University, and were performed in accordance with approved guidelines (Helsinki Declaration of 2013).

### Mouse models and treatments

All procedures were conducted in accordance with the “Guiding Principles in the Care and Use of Animals” (China) and were approved by the Animal Ethics Committee at Sun Yat-sen University. Four-week-old male NOD/SCID mice were purchased from Gempharmatech Inc (China). BALB/c nu/nu mice were purchased from the Experimental Animal Center of Sun Yat-Sen University. Approximately 5 × 10^6^ MGC803 cells were suspended in total of 100 μL PBS and Matrigel (1:1) mixture and implanted subcutaneously into the dorsal flank on both sides of the BALB/c nu/nu mice. When the tumor volume was approximately 50 mm^3^, the mice were randomized and treated intraperitoneally (i.p.) as indicated for five times per week. Tumor growth was monitored by calipers and volume was calculated using the equation: π/6 (length × width^2^). Body weight during the course of the study was also monitored. At the end of the studies, mice were sacrificed and tumors were dissected and weighed. To assess the effect of shRNA-mediated silencing of ATG4B on GCa xenograft tumor growth. 5 × 10^6^ MGC803 cells infected with lentivirus shControl or shATG4B were implanted into the dorsal flank of mice as above. Tumor volume and mice body weight was monitored. To assess the effect of ATG4B on GCa tumor metastasis in vivo. 5 × 10^6^ MKN45 cells expressing pLenti-Firefly Luciferase-EGFP were infected with lentiviruses expressing control or shATG4B shRNA before injecting into the peritoneal cavity of nude mice, monitored by bioluminescence. For the patient-derived xenograft, tumor sample was from patient with GCa adenocarcinoma. Characteristics of this patient are as follows: male, 42 years, Asian, primary GCa tumor, AJCC IB/grade 3, surgical sample. The GCa tumor tissues were divided into around 2 mm^3^ microtissues and were engrafted subcutaneously into the mice dorsal flank. The mice were randomized into five groups. The effect of drugs on the PDX tumor growth was monitored as above description. For orthotopic mice xenografts, four-week-old male NOD/SCID mice abdomen was incised, 1 × 10^7^ cells MKN45 cells stable expressing luciferase were injected into the subserosal layer of the stomach with a needle, and then the mice were randomized into four groups. Tumors grew in the stomach wall and metastases to the peritoneal cavity or other organs were monitored by Bioluminescence imaging. Mice body weight was also monitored. At the end of the studies, mice were sacrificed and tumors were dissected and weighed.

### Patient-derived organoid (PDO) culture

Organoids were cultured from fresh dissected tumors of PDX xenografts when the tumor size was around 500 mm^3^. Briefly, dissected tumors were finely minced and transferred to a 50 - mL conical tube, including a digestion mix consisting of serum-free DMEM/F-12 medium and 1 mg/mL collagenase IV (Sigma), and incubated for 1 h at 37 °C. Isolated organoids were mixed with 50 μL of Matrigel and seeded in 96-well plates. The culture medium contains phenol red-free DMEM/F-12 with primocin (50 mg/mL), B27 supplement (1×), FGF 7 (5 ng/mL), R-Spondin 3 (250 ng/mL), penicillin/streptomycin/glutamine (100 mg/mL), Y-27632 (5 mM), Hepes (10 mM), A83- 01 (500 nM), neuregulin 1 (5 nM), FGF 10 (20 ng/mL), EGF (100 ng/mL), SB202190 (500 nM), N-acetylcysteine (1.25 mM) and nicotinamide (5 mM). One milliliter of supplemented culture medium was added per well, and organoids were maintained in a 37 °C humidified atmosphere under 5% CO_2_. After one week, PDX-derived organoids were treated with DMSO or Am-F4a for another 3 days, representative images were taken under a fluorescence microscope, and cell viability in organoids was measured with Cell Titer-Glo.

### RNA-seq data analysis

AGS cells were transfected with ATG4B or control siRNA for 48 h before RNA extraction. Sequence libraries were validated by a MGISEQ2000 SE50 system (BGI Tech, Wuhan, China). Briefly, sequence reads were mapped to the reference human-genome assembly (GRCh37/hg19) with BWA and Bowtie 2. Change in expression of ≥ 1.5-fold (increase or decrease) was clustered with the k-means clustering algorithm in Cluster software 63. Gene Set Enrichment Analysis (GSEA v.4.1.0) was used to rank genes based on the shrunken limma log2 fold changes. The cluster Profiler package was used for gene ontology (GO) enrichment and KEGG pathway analysis, and *p* adjust (FDR) < 0.05 was considered as statistically significant.

### Statistics

Results were expressed as mean values ± S.E.M or mean values ± SD from at least 3 independent experiments. Statistics analysis were assessed using the Student’s 2-tailed *t* test. *p < 0.05 was considered as being significant.

## Results

### ATG4B is highly overexpressed in GCa clinical tumors and required for GCa cell growth

**Fig. 1:**
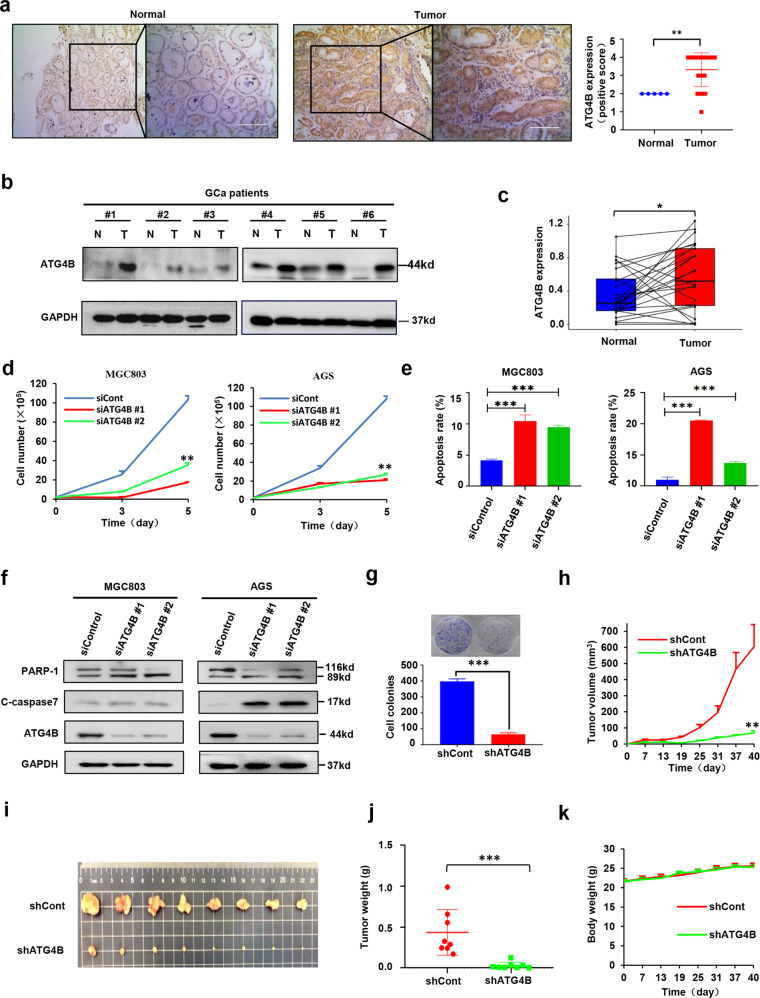
ATG4B is highly overexpressed in GCa clinical tumors and is required for GCa cell growth. Immunocytochemical staining for ATG4B expression in the human stomach normal and tumor tissues. Representative images were shown (scale bar, 100 μm). *P* value were calculated by two-tailed Student’s *t* test. **p < 0.001. **b**, **c** ATG4B protein levels were analyzed by immunoblotting analysis of in tissues (tumor and adjacent normal tissues) and representative images were shown. The data were statistically analyzed as in (**a**). **d** MGC803 and AGS cells were transfected with ATG4B or control siRNA. After indicated time points, viable cells were counted. Data shown are mean ± SD. Student’s *t* test. **p < 0.01, *n* = 3. **e** The apoptosis percentage of AGS and MGC803 cells transfected with ATG4B or control siRNA was detected by flow cytometry using AnnexinV/PI staining. Data shown are mean ± SD. Student’s *t* test. **p < 0.01, *n* = 3. **f** Immunobloting analysis of indicated protein in MGC803 and AGS cells transfected with ATG4B or control siRNA and incubated for 2 days. Representative blot, *n* = 3. **g** Colony formation of MGC803 cells infected with control or ATG4B shRNA lentiviruses. Representative images were shown, and colonies were counted. *n* = 3. **h**–**k** MGC803 cells infected with lentiviruses expressing control or shATG4B shRNA were injected into the dorsal flank of mice. Tumor growth and mice body weight were monitored. Representative tumor image and tumor weight at the end time point were captured. Mean tumor volume ± S.E.M (**h**), mean tumor weight ± S.E.M (**j**) and mean mice body weight ± S.E.M (**k**) were shown. Significance was calculated using two-tailed Student’s *t* test, **p < 0.01, ***p < 0.001.

Our previous studies revealed that ATG4B is a potential therapeutic target in colon cancer [14]. This promoted us to examine whether ATG4B exerted critical functions in other cancer types. Analysis of GEO datasets showed that the mRNA level of ATG4B was significantly higher in GCa tumors than in normal tissues (Fig. S1a). Consistently, the protein level of ATG4B was also significantly upregulated in gastric tumor tissues compared with normal tissues (Fig. 1a). Paired *t* test performed on the results of immunoblotting analysis of paired gastric normal and tumor tissues revealed that ATG4B was overexpressed in over 50% of the tumors (Fig. 1b, c and Fig. S1b). In addition, ATG4B protein was easily detectable in GCa cell lines (Fig. S1c). Kaplan–Meier survival analysis demonstrated that high ATG4B was strongly correlated with poor overall survival outcome of patients with GCa (Fig. S1d). These data indicate that ATG4B could be a potential GCa prognostic biomarker.

We next examined the function of ATG4B in GCa cell lines. Silencing ATG4B with two specific siRNA demonstrated that ATG4B knockdown significantly inhibited cell proliferation and induced apoptosis in GCa lines MGC803 and AGS (Fig. 1d–f, Fig. S1e). Consistently, ATG4B silencing also markedly decreased GCa cell’s colony formation ability (Fig. 1g). Encouraged by our in vitro results, we further assessed whether ATG4B contributes to GCa tumorigenesis in vivo. Employing GCa cell-based xenograft models, we found that ATG4B knockdown effectively blocked tumorigenesis and growth (Fig. 1h). At 40 days after the implantation of MGC803 cells, most of the mice with shATG4B-treated cells did not develop measurable tumors while the tumor volume in shControl group reached 600 mm^3^ on average (Fig. 1h, k). These results suggested that ATG4B be a critical gene in GCa tumorigenesis and growth.

### Identification of Am-F4a as a novel ATG4B inhibitor

**Fig. 2:**
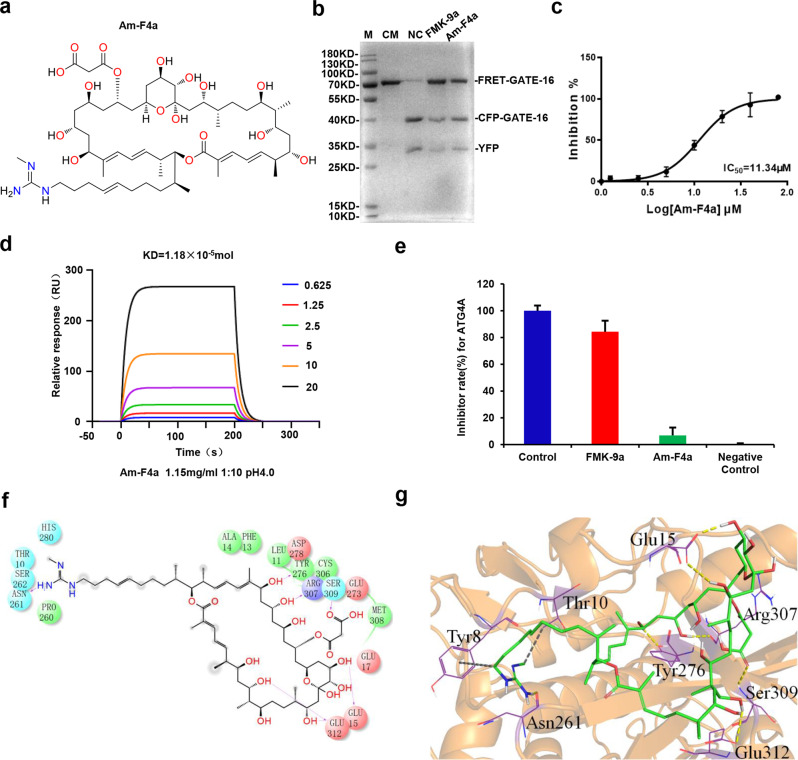
Identification of Am-F4a as a novel ATG4B inhibitor. **a** Chemical structure of Am-F4a. **b** ATG4B (0.75 μg/mL) was incubated with or without Am-F4a (10 μM) at 37 °C for 30 min, FRET-GATE-16 was then added and incubated at 37 °C for another 30 min. The inhibitory effect of Am-F4a was detected by SDS-PAGE according to the cleavage of FRET substrates. FMK-9a was used as a positive control. **c** The inhibitory effect and IC50 of Am-F4a on ATG4B activity were obtained from FRET assay. **d** Surface plasmon resonance (SPR) analysis of binding affinity of Am-F4a to ATG4B. The *K*_d_ value was calculated based on the fitted curves. **e** Am-F4a on ATG4A activity, the inhibitory effect of Am-F4a was detected by FREAT. **f**, **g** The predicted 2D and 3D binding mode of Am-F4a with ATG4B by molecular docking. In 3D mode, the protein and ligand Am-F4a are shown by cartoon and stick respectively, the highlighted interacting residues are shown by lines, the hydrogen bonds are labeled by yellow dashed lines, and the hydrophobic interaction are labeled by gray dashed lines.

Several ATG4B inhibitors have been identified and reported in the literature, [13] however, their efficacy and structural diversity are rather limited. To further explore the pharmacological functions of ATG4B in GCa, we firstly employed an in vitro FRET assay based on ATG4B catalytic activity to screen a natural product library, which contains more than 400 marine natural compounds. The 36-membered macrocyclic antitumor Azalomycin F4a (Am-F4a) is the main product of Streptomyces solisilvae HNM30702, which possesses one characteristic guanidino group rarely discovered in nature (Fig. 2a). As shown in Fig. 2b, c, Am-F4a effectively inhibited ATG4B activity with IC50 1.13 × 10^−5^ mol, in FRET assay. The gel-based assay which we previously developed [38] confirmed that Am-F4a strongly suppressed the proteolytic activity of ATG4B using FRET-GATE16 as substrate (Fig. 2b, c). Surface plasmon resonance (SPR) showed that Am-F4a could directly bind to ATG4B with a dissociation constant of 1.18 × 10^−5^ mol/L (Fig. 2d). Moreover, Am-F4a showed reasonable selectivity against ATG4A (Fig. 2e) with only 10% inhibitory activity at 20 μg/mL. To understand the inhibitory mechanism of Am-F4a at molecular level, we performed molecular docking of Am-F4a with ATG4B protein structure. The Am-F4a was docked into a binding pocket that is composed of Thr10, Leu11, Phe13, Ala14, Glu15, Glu17, Pro260, Asn261, Ser262, Glu273, Tyr276, Asp278, His280, Cys306, Arg307, Met308, Ser309 and Glu312 (Fig. 2f, g) as reported in our previous works. [14, 38] As shown in Fig. 2d, Am-F4a binds to the protein through potential hydrogen bonds with residues Glu15, Asn261, Tyr276, Arg307, Ser309 and Glu312. Especially, the guanidine group of Am-F4a could form hydrogen bonding interactions with Asn261 which is part of the regulatory loop (residues Lys259, Pro260, Asn261 and Ser262) covering the entrance of catalytic site and going through large conformational changes when LC3 interacts with ATG4B [38, 39]. These results collectively confirm that Am-F4a is a potent ATG4B inhibitor.

### Am-F4a suppresses autophagy flux in cells

**Fig. 3:**
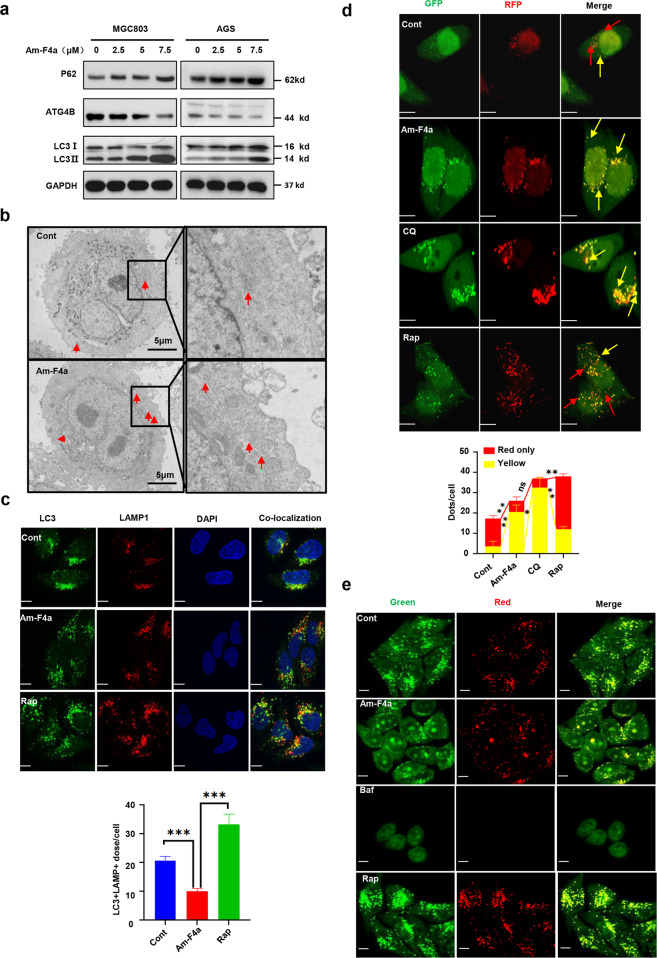
Am-F4a suppresses autophagy flux in cells. **a** Immunoblotting analysis of indicated protein in MGC803 and AGS cells treated with Am-F4a for 48 h. Representative blots, *n* = 3. **b** Representative images of transmission electron microscopy (TEM) exhibit ultrastructure of MGC803 cells treated with or without Am-F4a (10 μM) for 6 h. Red arrows indicate normal autophagosome structures. **c** Hela cells were treated with Am-F4a (10 μM) and Rap (1 μM) for 6 h. The colocalization of LC3 and LAMP1 puncta was examined and quantified (scale bar, 10 μm). Data are shown as mean ± SD, *n* = 3, Student’s *t* test, ***p < 0.001. **d** Hela cells expressing GFP-RFP-LC3 were treated with Am-F4a (10 μM) 、Rap (1 μM) and CQ (40 μM) for 6 h. The colocalization of GFP and RFP puncta was examined and quantified. Red arrows indicate GFP- or LC3-positive structure, yellow arrows indicate the colocalization of GFP and RFP. Fluorescence images of cells were recorded with fixation (scale bar, 10 μm). Data are shown as mean ± SD, *n* = 3, Student’s *t* test, *p < 0.05, **p < 0.01, ***p < 0.001. **e** Acridine Orange (AO) can indicate normal autolysosome structures of cells. Hela cells were treated with Am-F4a (10 μM), Rap (1 μM), Baf (0.5 μM) for 4 h, followed by AO (0.5 μg/mL) for 30 min. Fluorescence images of live cells were recorded without fixation (scale bar, 10 μm).

Given the crucial functions of ATG4B in autophagy, we examined whether its inhibitors affected autophagy process in GCa cells. LC3-II and P62 are validated indicators of autophagy activity of ATG4B [40]. Our immunoblotting results showed that Am-F4a caused significant accumulation of LC3-II and increased the protein level of P62 substantially, which suggested that Am-F4a treatment resulted in autophagosome accumulation in GCa cells (Fig. 3a). We used transmission electron microscopy to analyze the effect of Am-F4a on cellular ultrastructural morphological changes. Images showed that Am-F4a-treated cells accumulated more autophagosomes compared to the control cells (Fig. 3b). To further distinguish whether the accumulation of LC3-II caused by Am-F4a was a consequence of the increase in autophagosome formation or decrease of autophagosome degradation, we added chloroquine (CQ, a late phase of autophagy inhibitor, blocks autophagy predominantly by inhibiting autophagosome–lysome fusion) into Am-F4a treated cells and control cells. The results showed that CQ did not further enhance LC3-II accumulation induced by Am-F4a (Fig. S2), which indicated that the inhibition of autophagy caused by Am-F4a performed at the terminal stages, and the increase of LC3-II may result from a decrease of autophagosome degradation. Consistently, to monitor convergence of autophagosome and lysosome, we performed immunofluorescence colocalization of LC3 and LAMP1, our data further demonstrated that Am-F4a inhibits the fusion of autophagosomes and lysosomes (Fig. 3c, Fig. S3a). We next used a tandem mRFP-eGFP-LC3 construct to analyze the changes of autophagic flux as we previously reported [14]. Briefly, an acidic lysosome environment leads to reduction of the pH-sensitive green fluorescence (GFP) and keeps red fluorescence (RFP), autophagosomes display both mRFP and eGFP signals while lysosomes exhibit high mRFP signals and low GFP signals, which can be used to indicate the fusion step of autophagosomes with lysosomes. As expected, Rapamycin (Rap, an autophagy inducer) treatment resulted in greater red-only fluorescent puncta in the GCa cells, while Am-F4a- and chloroquine (CQ, an autophagy inhibitor) - treated cells showed high level of yellow puncta, from a mixture of GFP and RFP (Fig. 3d, Fig. S3b). The results indicated that Am-F4a might efficiently suppress autophagic flux. To examine whether the effect of Am-F4a on autophagy was caused by lysosome dysfunction, the pH of lysosome was measured by acridine orange (AO) [14]. Compared with control cells, there was no significant change in fluorescence in Am-F4a-treated cells, suggesting that Am-F4a did not cause the dysfunction of lysosome (Fig. 3e, Fig. S3c). Taken together, these data suggested that Am-F4a treatment result in the accumulation of autophagosomes and the suppression of autophagy flux in GCa cells.

### Am-F4a inhibits GCa cell growth via inhibition of ATG4B and improve anti-GCa efficacy of 5-FU

**Fig. 4:**
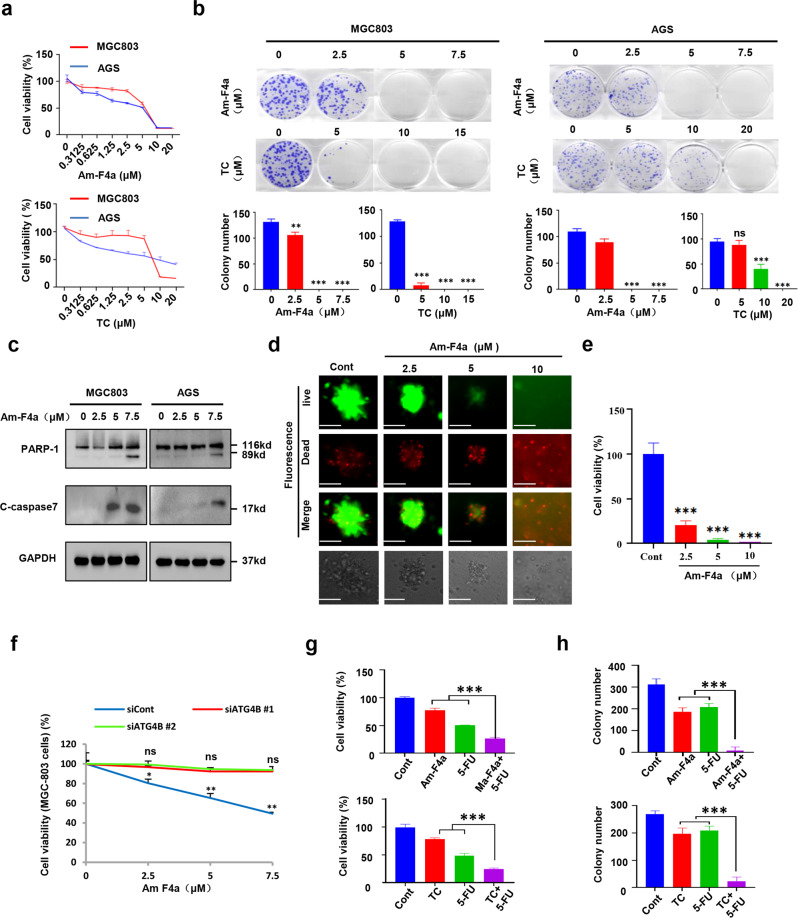
Am-F4a inhibits GCa cell growth via inhibition of ATG4B and improves anti-GCa efficacy of 5-FU. **a** Cell viability was evaluated by CCK-8 assay of MGC803 and AGS cells treated with Am-F4a and Tioconazole (TC). **b** Colony formation of MGC803 and AGS cells treated with Am-F4a at indicated concentration. Representative images were shown, and colonies were counted. **c** Immunoblotting analysis of cleaved PARP-1 and caspase 7 in MGC803 and AGS cells treated with Am-F4a at indicated concentration. Representative blots were shown. **d**, **e** PDX-derived organoids were treated with DMSO or indicated concentrations of Am-F4a for 4 days. Representative images were taken under a fluorescence microscope or a standard light microscope (scale bar, 50 μm) (**d**). Cell viability in organoids was measured with Cell Titer-Glo (**e**). **f** MGC803 cells were transfected with ATG4B or control siRNA for 48 h and then treated with Am-F4a for another 24 h. Viable cells were collected and counted. **g** MGC803 cells were treated with Am-F4a (4 μM) or TC (5 μM) alone, or in combination with 5-FU (2 μM) for 48 h. Viable cells were collected and counted. **h** MGC803 cells were treated with Am-F4a (4 μM) or TC (5 μM) alone, or in combination with 5-FU (2 μM) for 48 h. Colonies were stained and counted. All data shown above are mean ± SD, *n* = 3, Student’s *t* test, *p < 0.05, **p < 0.01, ***p < 0.001.

The prominent inhibition of Am-F4a on ATG4B activity and autophagy promoted us to examine whether Am-F4a mediates cell fate in GCa. Indeed, our results showed that Am-F4a significantly inhibited the growth of GCa cell MGC803 (IC50 = 5.391 μM) and AGS (IC50 = 3.136 μM), the antitumor effect of Am-F4a showed in a time-dependent manner (Fig. 4a, Fig. S4). Consistently, the growth inhibition by another established ATG4B inhibitor tioconazole (TC) was also observed; however, the IC50 values are higher than that of Am-F4a (Fig. 4a, Fig. S4a). In line with the inhibition of survival by ATG4B siRNA, treatment of MGC803 and AGS cells with Am-F4a or TC potently reduced cell colony formation and induced cell apoptosis, as compared to treatment with vehicle (Fig. 4b, c, Fig. S5). Due to PDX-derived organoids can precisely imitate clinical tumors in response to therapeutics, we treated organoids with Am-F4a to investigate their therapeutic potential. We found Am-F4a can significantly inhibit the growth of organoids (Fig. 4d, e). To provide further evidence that Am-F4a decreased the viability of GCa cells via inhibiting ATG4B activity, we used ATG4B siRNA approach to specifically silence ATG4B expression, results revealed that the effect of Am-F4a on GCa cell growth and apoptosis was obviously attenuated in ATG4B siRNA treated cells compared to control cells (Fig. 4f and Fig. S6). Since accumulating studies showed that autophagy inhibitors might improve the effects of chemotherapy in various cancer types [41], we wondered whether ATG4B inhibitors have the same function in GCa. As shown in Fig. 4g, pharmacological inhibition of ATG4B by Am-F4a or TC significantly enhanced antiproliferation of 5-FU treatment on GCa cells. Next, we performed clonogenic survival assay and found highly synergistic inhibitory effects on the survival of GCa cells after they were treated by a combination of 5-FU and either Am-F4a or TC (Fig. 4h, Fig. S7). These results collectively indicated that pharmacological inhibition of ATG4B with the novel inhibitor Am-F4a or TIC might effectively inhibit the viability of GCa cells via suppressing ATG4B activity and improve anti-GCa efficacy of 5-FU.

### Am-F4a inhibits GCa tumor growth and sensitizes 5-FU treatment

**Fig. 5:**
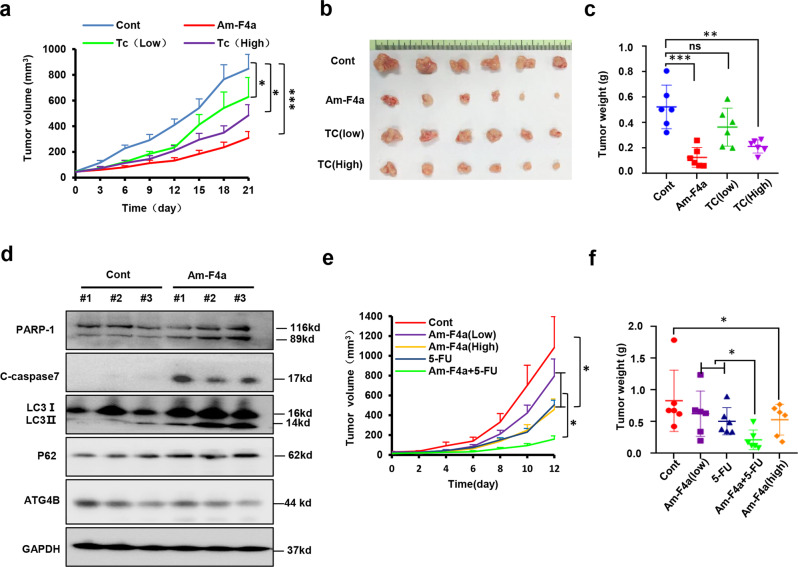
Am-F4a inhibits GCa tumor growth and sensitizes 5-FU treatment. **a**–**c** Effects of the indicated treatments (Am-F4a 5 mg/kg, TC low 50 mg/kg, TC high 100 mg/kg, or vehicle, i.p., 5 times per week) on the growth of MGC803 cell-based xenografts. Representative tumor image and tumor weight at the end time point were captured. Mean tumor volume ± S.E.M (**a**) and mean tumor weight ± S.E.M (**c**) were shown. **d** Immunoblotting analysis of indicated proteins in MGC803 xenograft tumors after 21 days of treatment with vehicle or Am-F4a, as in (**a**), representative images were shown. **e**, **f** Effects of the indicated treatments (Am-F4a low 2.5 mg/kg, Am-F4a high 5 mg/kg, 5-FU 20 mg/kg, Am-F4a low 2.5 mg/kg and 5-FU 20 mg/kg, or vehicle, i.p., 5 times per week) on the growth of PDX xenografts. Mean tumor volume ± S.E.M (**e**) and mean tumor weight ± S.E.M (**f**) were shown. All Data shown above was calculated using two-tailed Student’s *t* test, *n* = 6 mice per group, *p < 0.05, **p < 0.01, ***p < 0.001.

To evaluate the therapeutic efficacy of Am-F4a against GCa in vivo, we generated xenografts in immune-deficient nude mice from MGC803 cells. When the volume of tumors reached approximately 50 mm^3^, the mice were randomized into four groups and treated with vehicle, Am-F4a (5 mg/kg, five times per week), low dose TC (50 mg/kg, five times per week) and high dose TC (100 mg/kg, five times per week), respectively. Tumor volume and body weight of mice were measured every three days. Compared with the vehicle group, Am-F4a and high dose TC significantly suppressed tumor growth (Fig. 5a–c), no obvious mice body weight change was observed (Fig. S8a). Immunoblotting analysis of xenograft tumors demonstrated that Am-F4a significantly improved the level of cleaved-caspase-7, PARP-1, LC3 - II, and P62 protein, which indicated that Am-F4a might promote apoptosis and inhibit autophagy in the MGC803 xenograft tumors (Fig. 5d). Moreover, in a patient-derived GCa xenograft model (PDX), Am-F4a also effectively inhibited PDX tumor growth in a dose-dependent manner. Interestingly, in line with the results in vitro, the combination of Am-F4a and 5-FU synergistically decreased PDX tumor growth in mice (Fig. 5e, f and Fig. S8b, c), which indicated that Am-F4a might improve 5-FU treatment in GCa.

### ATG4B inhibition inhibits GC tumor metastasis

**Fig. 6:**
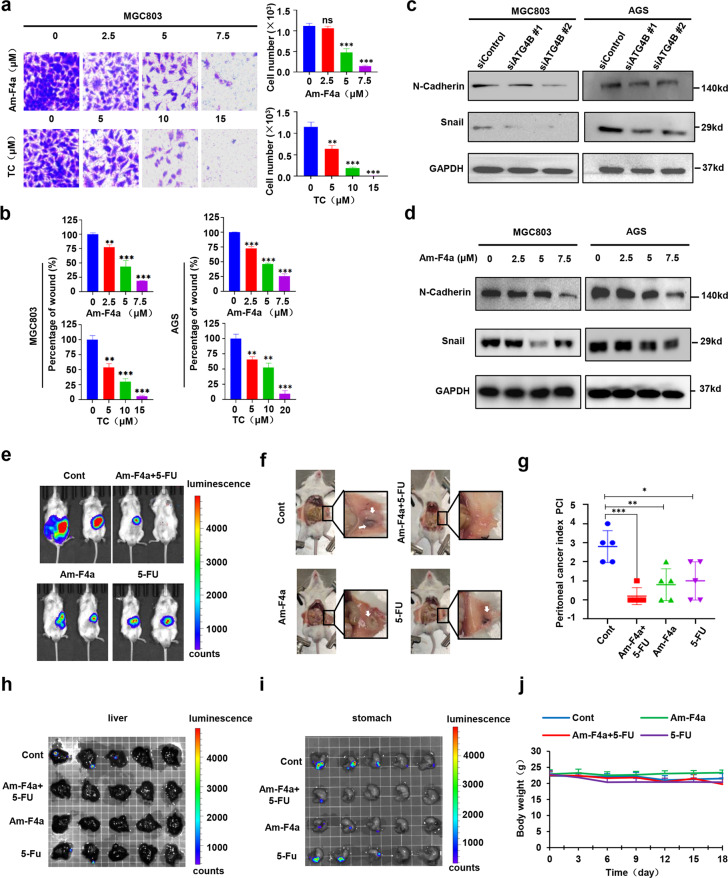
ATG4B inhibition inhibits GC tumor metastasis. **a** The invasion ability of MGC803 cells treated with Am-F4a, TC or vehicle at indicated concentration were evaluated by transwell assay. **b** The migration of GCa cells were determined using would healing assay. **c** Immunoblotting analysis of indicated proteins in MGC803 and AGS cells transfected with ATG4B or control siRNA and incubated for 48 h. **d** Immunoblotting analysis of indicated proteins in MGC803 and AGS cells treated with Am-F4a or vehicle for 48 h. **e** MKN45 cells stable expressing pLenti-Firefly Luciferase-EGFP were injected into the stomach wall of NCG mice, and randomly divided into four groups as indicated. Tumor growth in the stomach wall and metastases to the peritoneal cavity or other organs were monitored by bioluminescence at 14th day. **f, g** Representative image of the peritoneal metastasis (white arrows) of MKN45 cells in mice at the end of study (**f**). The peritoneal nodules were evaluated by peritoneal cancer index (PCI) (**g**). **h, i** Metastasis on the liver and stomach were monitored by bioluminescence at the end of study. **j** The body weight of the mice in all groups have no obvious change. All Data shown above was calculated using two-tailed Student’s *t* test, results of animal experiments were shown as mean ± S.E.M, *n* = 5 mice per group. Cell culture experiments were repeated at least three times and shown as mean ± SD. *p < 0.05, **p < 0.01, ***p < 0.001.

Metastasis is a major cause of GCa-associated death, therefore we investigated whether ATG4B inhibitors could be used as anti-metastatic therapy. Results showed that both ATG4B inhibitors and gene knockdown can significantly suppress migration and invasion of GC cells in vitro (Fig. 6a, b, Fig. S9a, b and S10). Meanwhile, the effect of Am-F4a on GCa cell migration and invasion was obviously attenuated in ATG4B siRNA treated cells compared to control cells, which indicated that Am-F4a functions via inhibiting ATG4B activity in GCa cells (Fig. S6b, c). Immunoblotting analysis demonstrated that ATG4B inhibition reduced the protein levels of key metastasis genes including Snail and N-cadherin in cells (Fig. 6c, d). Consistent with the in vitro results, ATG4B knockdown also effectively suppressed peritoneal metastasis in GCa xenograft models (Fig. S9c–h). To assess the effects of Am-F4a on the gastric tumor metastasis in vivo, we established the orthotopic GCa metastasis models using MKN45-luciferase cells. Tumor growth and metastasis were measured by bioluminescence (Fig. 6e). Compared to the vehicle group, Am-F4a could effectively inhibit tumor growth and metastasis, and the combination of Am-F4a and 5-FU showed the strongest inhibition (Fig. 6e). GCa metastasis typically occurs in livers, peritoneum and lungs. Peritoneal cancer index (PCI) is an effective indicator to evaluate peritoneal metastasis. After 18 days’ treatment, the mice were then euthanized and macroscopic appearance of GCa peritoneal metastases is shown in Fig. 6f. Peritoneal metastatic nodules could be observed in all the vehicle treated mice, whereas only a few mice showed smaller metastatic nodules in the drug-treated mice. Consistently, Am-F4a and 5-FU effectively reduced mice PCI, and their combination resulted in lowest PCI (Fig. 6g). Moreover, Am-F4a also effectively inhibited the liver metastasis, suppressed GCa orthotopic tumor growth and sensitize tumor to 5-FU treatment (Fig. 6h). Taken together, our data suggested that Am-F4a might be an effective agent for GC tumor metastasis therapy, especially combined with 5-FU treatment.

## Discussion

In the past two decades, the prognosis for localized GCa has greatly improved, however, the outcomes of advanced disease remain poorly [42]. Recently, promising progress has been made in targeted therapy for advanced GCa. Unfortunately, the options for targeted therapy and the number of patients benefited are limited. The Discovery of new therapeutic targets and the development of novel agents for advanced GC therapy are still urgently needed. Here, we demonstrated that ATG4B overexpression is significantly correlated with poor survival in patients with GC and is a critical dependency of GCa cell growth. Am-F4a, as a novel ATG4B inhibitor, potently inhibited GCa tumor growth and metastasis in vitro and in vivo.

ATG4B plays critical physiological roles in normal development and diseases via promoting autophagosome formation and autophagy process. In cancers, ATG4B has been identified as a potential therapeutic target in a number of cancers, including colon, glioblastoma, osteosarcoma. Our study here advanced our understanding of the functions of ATG4B in GCa. Analysis of patient samples showed that ATG4B is overexpressed in GCa tumors, and its high expression correlated with patients’ poor survival. Silencing ATG4B with specific siRNAs significantly inhibited GCa cell autophagy, survival and promoted their apoptosis. Notably, knockdown of ATG4B potently suppressed tumorigenesis and tumor growth in mice GCa xenograft models. Therefore, ATG4B is essential for GCa cell survival and tumor growth.

Recently, several ATG4B inhibitors were discovered and used to explore the physiological functions of ATG4B in cancers. In line with the effect of ATG4B silencing in cancer cells, most of ATG4B inhibitors exerted anticancer activity via suppressing autophagy process. However, the efficacy and diversity of current ATG4B inhibitors are limited. A potent and selective ATG4B inhibitor is still needed to explore the therapeutic potentials of ATG4B inhibition. Here, we discovered a novel ATG4B inhibitor, Am-F4a, from a natural product library. FRET assay and SPR analysis demonstrated that Am-F4a selectively bound to ATG4B protein and potently inhibited ATG4B activity. Molecular docking study indicated that Am-F4a could form several H-bonds with ATG4B in binding pocket site 5 (Fig. 2f, g), and especially the H-bond between guanidine group of Am-F4a and residue Asn261 in the regulatory loop (residues 259 to 262) of the entrance of catalytic site makes Am-F4a a potent inhibitor for ATG4B from the atomic level perspective. In cancer cells, Am-F4a effectively inhibited autophagic flux, we found that Am-F4a treatment was able to inhibit the autophagosome–lysosome fusion, not autolysosome accumulation. The effects of Am-F4a on LC3B-II accumulation, but not pro-LC3B or LC3B-I accumulation, which are consistent with reduced LC3B-II delipidation or deconjugation mediated by ATG4B, thus result in the failure of the autophagosome–lysosome fusion. Due to the fusion of autophagosome and lysosome was suppressed, the accumulation of LC3-II in cells caused by Am-F4a was mainly present in autophagosomes, not autolysosome. These data suggested that Am-F4a might be an excellent lead compound to uncover the pharmacological roles of ATG4B in cancers.

Notably, our data revealed that Am-F4a significantly inhibited GCa cell growth and triggered their apoptosis via suppressing ATG4B activity. Moreover, Am-F4a potently inhibited tumor growth in both cell line-derived and patient-derived GCa xenograft models without obvious toxicity. As shown in Figs. 4c, 5d, the cleavage of PARP1 and caspase-7 mediated by Am-F4a. PARP1 is a substrate of caspase-3, whether Am-F4a increases cleavage of PARP1 via mediating caspase-3 activity requires further exploration. Chemotherapy resistance and metastasis are the main obstacles for advanced GCa therapy. Several studies indicated that ATG4B inhibition might be helpful to overcome drug resistance; however, the pharmacological roles of ATG4B in cancer metastasis remained unknown. We found that genetic and pharmacologic inhibition of ATG4B significantly suppressed GCa cell migration and invasion in vitro. Employing orthotopic GCa metastasis xenograft models, we demonstrated herein that Am-F4a could markedly block liver and peritoneal metastasis, and sensitized tumors to 5-FU treatment. 5-FU chemotherapy is the first choice for advanced GCa therapy, but its effectiveness is limited by drug resistance. Therefore, this study suggested that ATG4B inhibition might have broad clinical utilities in GCa. Our data showed that the effect of ATG4B inhibition on GCa cells might be through suppressing the autophagy process. Genetic and pharmacological inhibition of ATG4B significantly inhibited autophagic flux and accumulated lipidated LC3 in autolysosomes both in vitro and in vivo. However, the overall impact of ATG4B inhibition on tumor growth is unlikely limited to suppress autophagy. A previous study showed that ATG4B knockdown increased CCND1 expression and resulted in colorectal cancer cell growth arrest independent of autophagy [43]. Our study also showed that ATG4B inhibition increased CCND1 expression in GCa cells (Fig. S11). Moreover, gene ontology (GO) analysis of the transcripts changed by ATG4B knockdown in GCa cells revealed that, in addition to autophagy, multiple pathways involved in the cell cycle, survival and metastasis were also changed (Fig. S12). Therefore, ATG4B inhibition blocks GCa tumor growth and metastasis, possibly through inhibiting multiple pathways, including autophagy.

## Conclusions

In summary, we demonstrated here that ATG4B might be a potential therapeutic target for advanced GCa and identified a natural product Am-F4a as a novel ATG4B inhibitor. Am-F4a significantly inhibited GCa cell survival and tumor growth via suppressing ATG4B activity and downstream processes. Furthermore, in orthotopic GCa metastasis xenograft models, we found that Am-F4a could effectively block GCa liver and peritoneal metastasis, and sensitized tumors to 5-FU treatment. These data collectively suggested that ATG4B inhibition might have a broad clinical utility in GCa, and Am-F4a might be an efficacious antitumor therapeutic agent.

## Supplementary information

Coauthors confirmation on author list change

Supplemental material

Original western blots

Checklist

## Data Availability

All data generated or analyzed during this study are available from the corresponding author on reasonable request.
