# Supplementary material for: Targeting autophagy peptidase ATG4B with a novel natural product inhibitor Azalomycin F4a for advanced gastric cancer
Source: Cell Death Dis. 2022 Feb 18;13(2):161. doi: 10.1038/s41419-022-04608-z (PMC8858318; doi:10.1038/s41419-022-04608-z)
Supplement: Supplementary file 1 — Coauthors confirmation on author list change [file 41419_2022_4608_MOESM1_ESM.pdf]

| | |
|---|---|
| 主　题: | 回复: Confirming co-authors list changes for our manuscript submitted to Cell Death & Disease |
| 发件人: | "钟林" <zhonglin6@mail2.sysu.edu.cn> 2022-1-27 17:14:07 |
| 收件人: | "王军舰" <wangjj87@mail.sysu.edu.cn> |

I agree to these author list changes.

LinZhong

---------------原始邮件---------------

**发件人:** 王军舰<wangjj87@mail.sysu.edu.cn>
**发送时间:** 2022-01-27(周四) 17:02
**收件人:** zhonglin6<zhonglin6@mail2.sysu.edu.cn>, zhoushn3<zhoushn3@mail.sysu.edu.cn>, yangb23<yangb23@mail.sysu.edu.cn>, zhangzhh68<zhangzhh68@mail2.sysu.edu.cn>, wxj0250<wxj0250@163.com>, guoyf36<guoyf36@mail2.sysu.edu.cn>, huangwf26<huangwf26@mail2.sysu.edu.cn>, wangqq36<wangqq36@mail2.sysu.edu.cn>, caigd3<caigd3@mail2.sysu.edu.cn>, xiaf6<xiaf6@mail2.sysu.edu.cn>, mash33<mash33@mail2.sysu.edu.cn>, nieyichu2<nieyichu2@126.com>, leijp<leijp@mail.sysu.edu.cn>, limin65<limin65@mail.sysu.edu.cn>, liupq<liupq@mail.sysu.edu.cn>, dengwb5<dengwb5@mail.sysu.edu.cn>, yonghongliu<yonghongliu@scsio.ac.cn>, wangjunfeng<wangjunfeng@scsio.ac.cn>, hanfh<hanfh@mail.sysu.edu.cn>
**主题:** Confirming co-authors list changes for our manuscript submitted to Cell Death & Disease

Dear ,

I am very pleased to inform you that our manuscript CDDIS-21-2849RR entitled "Targeting autophagy peptidase ATG4B with a novel natural product inhibitor Azalomycin F4a for advanced gastric cancer" has been provisionally accepted for publication in Cell Death & Disease.

Since we changed author list from our original submission (CDDIS-21-2849: Lin Zhong[a†], Bin Yang[a†], Zhenhua zhang[b†], Xiaojuan Wang[d], Yinfeng Guo[e], Weifeng Huang[e], Qianqian Wang[e], Guodi Cai[e], Fan Xia[e], Shengning Zhou[a], Shuai Ma[a], Yichu Nie[b], Jinping Lei[e], Min Li[e, f], Peiqing Liu[e, f], Wenbin Deng[b], Yonghong Liu[c], Junfeng Wang[c]*, Fanghai Han[a]*, Junjian Wang[e, f]*) to current author list (CDDIS-21-2849RR:Lin Zhong[a†], Bin Yang[a†], Zhenhua zhang[b†], Junfeng Wang[c]*, Xiaojuan Wang[d], Yinfeng Guo[e], Weifeng Huang[e], Qianqian Wang[e], Guodi Cai[e], Fan Xia[e], Shengning Zhou[a], Shuai Ma[a], Yichu Nie[b], Jinping Lei[e], Min Li[e, f], Peiqing Liu[e, f], Wenbin Deng[b], Yonghong Liu[c], Fanghai Han[a]*, Junjian Wang[e, f]*) .

We need all the coauthors to confirm whether they agree to these changes. If you agree to these changes, please reply "I agree to these author list changes" followed by your name, otherwise, please let me know if you have any questions!

Thank you for your help!

Junjian Wang

本邮件及其附件含有发送给特定个人和用于特定目的的信息。如果您不是预期的收件人，请立即删除本邮件并通知发件人。严禁任何非预期的收件人使用、传播、分发或复制本邮件或其附件。
This email and its attachments may contain confidential information intended for a specific individual and purpose. If you are not the intended recipient, you should delete this email and notify the sender immediately. Any use, dissemination, distribution, or copying of this email or its attachments by persons other than the intended recipient(s), is strictly prohibited.

| | |
|---|---|
| 主　题: | 回复：Confirming co-authors list changes for our manuscript submitted to Cell Death & Disease |
| 发件人: | "yangb23@mail.sysu.edu.cn" <yangb23@mail.sysu.edu.cn> 2022-1-27 18:17:59 |
| 收件人: | "王军舰" <wangjj87@mail.sysu.edu.cn> |

Dear,

I agree to these author list changes, Thank you!

Best regards

Bin Yang

-------- 原始邮件 --------
发件人： 王俊锋 <wangjunfeng@scsio.ac.cn>
日期： 2022年1月27日周四 傍晚5:18
收件人： 王军舰 <wangjj87@mail.sysu.edu.cn>
抄送： zhonglin6@mail2.sysu.edu.cn, yangb23@mail.sysu.edu.cn, zhangzhh68@mail2.sysu.edu.cn, wxj0250@163.com, guoyf36@mail2.sysu.edu.cn, huangwf26@mail2.sysu.edu.cn, wangqq36@mail2.sysu.edu.cn, caigd3@mail2.sysu.edu.cn, xiaf6@mail2.sysu.edu.cn, zhoushn3@mail.sysu.edu.cn, mash33@mail2.sysu.edu.cn, nieyichu2@126.com, leijp@mail.sysu.edu.cn, limin65@mail.sysu.edu.cn, liupq@mail.sysu.edu.cn, dengwb5@mail.sysu.edu.cn, yonghongliu@scsio.ac.cn, hanfh@mail.sysu.edu.cn
主 题： Re: Confirming co-authors list changes for our manuscript submitted to Cell Death & Disease

Dear,

I agree to these author list changes, Thank you!

Best regards

Junfeng Wang

-----原始邮件-----
**发件人:**"王军舰" <wangjj87@mail.sysu.edu.cn>
**发送时间:**2022-01-27 17:02:54 (星期四)
**收件人:** zhonglin6@mail2.sysu.edu.cn, yangb23@mail.sysu.edu.cn, zhangzhh68@mail2.sysu.edu.cn, wangjunfeng@scsio.ac.cn, wxj0250@163.com, guoyf36@mail2.sysu.edu.cn, huangwf26@mail2.sysu.edu.cn, wangqq36@mail2.sysu.edu.cn, caigd3@mail2.sysu.edu.cn, xiaf6@mail2.sysu.edu.cn, zhoushn3@mail.sysu.edu.cn, mash33@mail2.sysu.edu.cn, nieyichu2@126.com, leijp@mail.sysu.edu.cn, limin65@mail.sysu.edu.cn, liupq@mail.sysu.edu.cn, dengwb5@mail.sysu.edu.cn, yonghongliu@scsio.ac.cn, hanfh@mail.sysu.edu.cn
**抄送:**
**主题:** Confirming co-authors list changes for our manuscript submitted to Cell Death & Disease

Dear ,

I am very pleased to inform you that our manuscript CDDIS-21-2849RR entitled "Targeting autophagy peptidase ATG4B with a novel natural product inhibitor Azalomycin F4a for advanced gastric cancer" has been provisionally accepted for publication in Cell Death & Disease.

Since we changed author list from our original submission (CDDIS-21-2849: Lin Zhong[a†], Bin Yang[a†], Zhenhua zhang[b†], Xiaojuan Wang[d], Yinfeng Guo[e], Weifeng Huang[e], Qianqian Wang[e], Guodi Cai[e], Fan Xia[e], Shengning Zhou[a], Shuai Ma[a], Yichu Nie[b], Jinping Lei[e], Min Li[e, f], Peiqing Liu[e, f], Wenbin Deng[b], Yonghong Liu[c], Junfeng Wang[c]*, Fanghai Han[a]*, Junjian Wang[e, f]*) to current author list (CDDIS-21-2849RR:Lin Zhong[a†], Bin Yang[a†], Zhenhua zhang[b†], Junfeng Wang[c]*, Xiaojuan Wang[d], Yinfeng Guo[e], Weifeng Huang[e], Qianqian Wang[e], Guodi Cai[e], Fan Xia[e], Shengning Zhou[a], Shuai Ma[a], Yichu Nie[b], Jinping Lei[e], Min Li[e, f], Peiqing Liu[e, f], Wenbin Deng[b], Yonghong Liu[c], Fanghai Han[a]*, Junjian Wang[e, f]*).

We need all the coauthors to confirm whether they agree to these changes. If you agree to these changes, please reply “I agree to these author list changes” followed by your name, otherwise, please let me know if you have any questions!

Thank you for your help!

Junjian Wang

---

本邮件及其附件含有发送给特定个人和用于特定目的的信息。如果您不是预期的收件人，请立即删除本邮件并通知发件人。严禁任何非预期的收件人使用、传播、分发或复制本邮件或其附件。

This email and its attachments may contain confidential information intended for a specific individual and purpose. If you are not the intended recipient, you should delete this email and notify the sender immediately. Any use, dissemination, distribution, or copying of this email or its attachments by persons other than the intended recipient(s), is strictly prohibited.

| | | |
|---|---|---|
| 主　题: | Re:Confirming co-authors list changes for our manuscript submitted to Cell Death & Disease | |
| 发件人: | "张振华" <zhangzhh68@mail2.sysu.edu.cn> | 2022-1-27 19:50:33 |
| 收件人: | "王军舰" <wangjj87@mail.sysu.edu.cn> | |

I agree to these author list changes  Zhenhua zhang

------------------ Original ------------------

**From:** "王军舰"<wangjj87@mail.sysu.edu.cn>;
**Date:** Thu, Jan 27, 2022 05:03 PM
**To:** "zhonglin6"<zhonglin6@mail2.sysu.edu.cn>; "yangb23"<yangb23@mail.sysu.edu.cn>; "张振华" <zhangzhh68@mail2.sysu.edu.cn>; "wangjunfeng"<wangjunfeng@scsio.ac.cn>; "wxj0250"<wxj0250@163.com>; "guoyf36" <guoyf36@mail2.sysu.edu.cn>; "huangwf26"<huangwf26@mail2.sysu.edu.cn>; "wangqq36"<wangqq36@mail2.sysu.edu.cn>; "caigd3"<caigd3@mail2.sysu.edu.cn>; "xiaf6"<xiaf6@mail2.sysu.edu.cn>; "zhoushn3"<zhoushn3@mail.sysu.edu.cn>; "mash33"<mash33@mail2.sysu.edu.cn>; "nieyichu2"<nieyichu2@126.com>; "leijp"<leijp@mail.sysu.edu.cn>; "limin65" <limin65@mail.sysu.edu.cn>; "liupq"<liupq@mail.sysu.edu.cn>; "dengwb5"<dengwb5@mail.sysu.edu.cn>; "yonghongliu" <yonghongliu@scsio.ac.cn>; "hanfh"<hanfh@mail.sysu.edu.cn>;
**Subject:** Confirming co-authors list changes for our manuscript submitted to Cell Death & Disease

Dear ,

I am very pleased to inform you that our manuscript CDDIS-21-2849RR entitled ″Targeting autophagy peptidase ATG4B with a novel natural product inhibitor Azalomycin F4a for advanced gastric cancer″ has been provisionally accepted for publication in Cell Death & Disease.

Since we changed author list from our original submission (CDDIS-21-2849: Lin Zhong[a†], Bin Yang[a†], Zhenhua zhang[b†], Xiaojuan Wang[d], Yinfeng Guo[e], Weifeng Huang[e], Qianqian Wang[e], Guodi Cai[e], Fan Xia[e], Shengning Zhou[a], Shuai Ma[a], Yichu Nie[b], Jinping Lei[e], Min Li[e, f], Peiqing Liu[e, f], Wenbin Deng[b], Yonghong Liu[c], Junfeng Wang[c]*, Fanghai Han[a]*, Junjian Wang[e, f]*) to current author list (CDDIS-21-2849RR:Lin Zhong[a†], Bin Yang[a†], Zhenhua zhang[b†], Junfeng Wang[c]*, Xiaojuan Wang[d], Yinfeng Guo[e], Weifeng Huang[e], Qianqian Wang[e], Guodi Cai[e], Fan Xia[e], Shengning Zhou[a], Shuai Ma[a], Yichu Nie[b], Jinping Lei[e], Min Li[e, f], Peiqing Liu[e, f], Wenbin Deng[b], Yonghong Liu[c], Fanghai Han[a]*, Junjian Wang[e, f]*) .

We need all the coauthors to confirm whether they agree to these changes. If you agree to these changes, please reply “I agree to these author list changes” followed by your name, otherwise, please let me know if you have any questions!

Thank you for your help!

Junjian Wang

---

本邮件及其附件含有发送给特定个人和用于特定目的的信息。如果您不是预期的收件人，请立即删除本邮件并通知发件人。严禁任何非预期的收件人使用、传播、分发或复制本邮件或其附件。
This email and its attachments may contain confidential information intended for a specific individual and purpose. If you are not the intended recipient, you should delete this email and notify the sender immediately. Any use, dissemination, distribution, or copying of this email or its attachments by persons other than the intended recipient(s), is strictly prohibited.

| 主　题: | Re: Confirming co-authors list changes for our manuscript submitted to Cell Death & Disease |
|---|---|
| 发件人: | "王俊锋" <wangjunfeng@scsio.ac.cn> 2022-1-27 17:18:31 |
| 收件人: | "王军舰" <wangjj87@mail.sysu.edu.cn> |
| 抄　送: | zhonglin6@mail2.sysu.edu.cn, yangb23@mail.sysu.edu.cn, zhangzhh68@mail2.sysu.edu.cn, wxj0250@163.com, guoyf36@mail2.sysu.edu.cn, huangwf26@mail2.sysu.edu.cn, wangqq36@mail2.sysu.edu.cn, caigd3@mail2.sysu.edu.cn, xiaf6@mail2.sysu.edu.cn, zhoushn3@mail.sysu.edu.cn, mash33@mail2.sysu.edu.cn, nieyichu2@126.com, leijp@mail.sysu.edu.cn, limin65@mail.sysu.edu.cn, liupq@mail.sysu.edu.cn, dengwb5@mail.sysu.edu.cn, yonghongliu@scsio.ac.cn, hanfh@mail.sysu.edu.cn |

Dear,

I agree to these author list changes, Thank you!

Best regards

Junfeng Wang

-----原始邮件-----
**发件人:**"王军舰" <wangjj87@mail.sysu.edu.cn>
**发送时间:**2022-01-27 17:02:54 (星期四)
**收件人:** zhonglin6@mail2.sysu.edu.cn, yangb23@mail.sysu.edu.cn, zhangzhh68@mail2.sysu.edu.cn, wangjunfeng@scsio.ac.cn, wxj0250@163.com, guoyf36@mail2.sysu.edu.cn, huangwf26@mail2.sysu.edu.cn, wangqq36@mail2.sysu.edu.cn, caigd3@mail2.sysu.edu.cn, xiaf6@mail2.sysu.edu.cn, zhoushn3@mail.sysu.edu.cn, mash33@mail2.sysu.edu.cn, nieyichu2@126.com, leijp@mail.sysu.edu.cn, limin65@mail.sysu.edu.cn, liupq@mail.sysu.edu.cn, dengwb5@mail.sysu.edu.cn, yonghongliu@scsio.ac.cn, hanfh@mail.sysu.edu.cn
**抄送:**
**主题:** Confirming co-authors list changes for our manuscript submitted to Cell Death & Disease

Dear ,

I am very pleased to inform you that our manuscript CDDIS-21-2849RR entitled "Targeting autophagy peptidase ATG4B with a novel natural product inhibitor Azalomycin F4a for advanced gastric cancer" has been provisionally accepted for publication in Cell Death & Disease.

Since we changed author list from our original submission (CDDIS-21-2849: Lin Zhong[a†], Bin Yang[a†], Zhenhua zhang[b†], Xiaojuan Wang[d], Yinfeng Guo[e], Weifeng Huang[e], Qianqian Wang[e], Guodi Cai[e], Fan Xia[e], Shengning Zhou[a], Shuai Ma[a], Yichu Nie[b], Jinping Lei[e], Min Li[e, f], Peiqing Liu[e, f], Wenbin Deng[b], Yonghong Liu[c], Junfeng Wang[c*], Fanghai Han[a*], Junjian Wang[e, f*]) to current author list (CDDIS-21-2849RR:Lin Zhong[a†], Bin Yang[a†], Zhenhua zhang[b†], Junfeng Wang[c*], Xiaojuan Wang[d], Yinfeng Guo[e], Weifeng Huang[e], Qianqian Wang[e], Guodi Cai[e], Fan Xia[e], Shengning Zhou[a], Shuai Ma[a], Yichu Nie[b], Jinping Lei[e], Min Li[e, f], Peiqing Liu[e, f], Wenbin Deng[b], Yonghong Liu[c], Fanghai Han[a*], Junjian Wang[e, f*]).

We need all the coauthors to confirm whether they agree to these changes. If you agree to these changes, please reply "I agree to these author list changes" followed by your name, otherwise, please let me know if you have any questions!

Thank you for your help!

Junjian Wang

---

本邮件及其附件含有发送给特定个人和用于特定目的的信息。如果您不是预期的收件人，请立即删除本邮件并通知发件人。严禁任何非预期的收件人使用、传播、分发或复制本邮件或其附件。
This email and its attachments may contain confidential information intended for a specific individual and purpose. If you are not the intended recipient, you should delete this email and notify the sender immediately. Any use, dissemination, distribution, or copying of this email or its attachments by persons other than the intended recipient(s), is strictly prohibited.

| | |
|---|---|
| 主　题: | Re: Confirming co-authors list changes for our manuscript submitted to Cell Death & Disease |
| 发件人: | "WANG Xiaojuan" <wxj0250@163.com> 2022-1-27 22:44:03 |
| 收件人: | "王军舰" <wangjj87@mail.sysu.edu.cn> |
| 抄　送: | zhonglin6 <zhonglin6@mail2.sysu.edu.cn>, yangb23 <yangb23@mail.sysu.edu.cn>, zhangzhh68 <zhangzhh68@mail2.sysu.edu.cn>, wangjunfeng <wangjunfeng@scsio.ac.cn>, guoyf36 <guoyf36@mail2.sysu.edu.cn>, huangwf26 <huangwf26@mail2.sysu.edu.cn>, wangqq36 <wangqq36@mail2.sysu.edu.cn>, caigd3 <caigd3@mail2.sysu.edu.cn>, xiaf6 <xiaf6@mail2.sysu.edu.cn>, zhoushn3 <zhoushn3@mail.sysu.edu.cn>, mash33 <mash33@mail2.sysu.edu.cn>, nieyichu2 <nieyichu2@126.com>, leijp <leijp@mail.sysu.edu.cn>, limin65 <limin65@mail.sysu.edu.cn>, liupq <liupq@mail.sysu.edu.cn>, dengwb5 <dengwb5@mail.sysu.edu.cn>, yonghongliu <yonghongliu@scsio.ac.cn>, hanfh <hanfh@mail.sysu.edu.cn> |

Dear Dr. Wang,

I agree to these author list changes.

Kinds,
Wang Xiaojuan

---- Replied Message ----

| | |
|---|---|
| From | 王军舰<wangjj87@mail.sysu.edu.cn> |
| Date | 01/27/2022 17:02 |
| To | zhonglin6<zhonglin6@mail2.sysu.edu.cn>,<br>yangb23<yangb23@mail.sysu.edu.cn>,<br>zhangzhh68<zhangzhh68@mail2.sysu.edu.cn>,<br>wangjunfeng<wangjunfeng@scsio.ac.cn>,<br>wxj0250<wxj0250@163.com>,<br>guoyf36<guoyf36@mail2.sysu.edu.cn>,<br>huangwf26<huangwf26@mail2.sysu.edu.cn>,<br>wangqq36<wangqq36@mail2.sysu.edu.cn>,<br>caigd3<caigd3@mail2.sysu.edu.cn>,<br>xiaf6<xiaf6@mail2.sysu.edu.cn>,<br>zhoushn3<zhoushn3@mail.sysu.edu.cn>,<br>mash33<mash33@mail2.sysu.edu.cn>,<br>nieyichu2<nieyichu2@126.com>,<br>leijp<leijp@mail.sysu.edu.cn>,<br>limin65<limin65@mail.sysu.edu.cn>,<br>liupq<liupq@mail.sysu.edu.cn>,<br>dengwb5<dengwb5@mail.sysu.edu.cn>,<br>yonghongliu<yonghongliu@scsio.ac.cn>,<br>hanfh<hanfh@mail.sysu.edu.cn> |
| Subject | Confirming co-authors list changes for our manuscript submitted to Cell Death & Disease |

Dear ,

I am very pleased to inform you that our manuscript CDDIS-21-2849RR entitled "Targeting autophagy peptidase ATG4B with a novel natural product inhibitor Azalomycin F4a for advanced gastric cancer" has been provisionally accepted for publication in Cell Death & Disease.

Since we changed author list from our original submission (CDDIS-21-2849: Lin Zhong[a†], Bin Yang[a†], Zhenhua zhang[b†], Xiaojuan Wang[d], Yinfeng Guo[e], Weifeng Huang[e], Qianqian Wang[e], Guodi Cai[e], Fan Xia[e], Shengning Zhou[a], Shuai Ma[a], Yichu Nie[b], Jinping Lei[e], Min Li[e, f], Peiqing Liu[e, f], Wenbin Deng[b], Yonghong Liu[c], Junfeng Wang[c*], Fanghai Han[a*], Junjian Wang[e, f*]) to current author list (CDDIS-21-2849RR:Lin Zhong[a†], Bin Yang[a†], Zhenhua zhang[b†],

Junfeng Wang[c]*, Xiaojuan Wang[d], Yinfeng Guo[e], Weifeng Huang[e], Qianqian Wang[e], Guodi Cai[e], Fan Xia[e], Shengning Zhou[a], Shuai Ma[a], Yichu Nie[b], Jinping Lei[e], Min Li[e, f], Peiqing Liu[e, f], Wenbin Deng[b], Yonghong Liu[c], Fanghai Han[a]*, Junjian Wang[e, f]*) .

We need all the coauthors to confirm whether they agree to these changes. If you agree to these changes, please reply “I agree to these author list changes” followed by your name, otherwise, please let me know if you have any questions!

Thank you for your help!

Junjian Wang

---

本邮件及其附件含有发送给特定个人和用于特定目的的信息。如果您不是预期的收件人，请立即删除本邮件并通知发件人。严禁任何非预期的收件人使用、传播、分发或复制本邮件或其附件。
This email and its attachments may contain confidential information intended for a specific individual and purpose. If you are not the intended recipient, you should delete this email and notify the sender immediately. Any use, dissemination, distribution, or copying of this email or its attachments by persons other than the intended recipient(s), is strictly prohibited.

| | |
|---|---|
| 主　题: | Re:Re: Confirming co-authors list changes for our manuscript submitted to Cell Death & Disease |
| 发件人: | "郭银锋" <guoyf36@mail2.sysu.edu.cn> 2022-1-28 10:41:33 |
| 收件人: | "Mail.sysu.edu.cn" <liupq@mail.sysu.edu.cn>, "WANG Xiaojuan" <wxj0250@163.com> |
| 抄　送: | "王军舰" <wangjj87@mail.sysu.edu.cn>, zhonglin6 <zhonglin6@mail2.sysu.edu.cn>, yangb23 <yangb23@mail.sysu.edu.cn>, zhangzhh68 <zhangzhh68@mail2.sysu.edu.cn>, wangjunfeng <wangjunfeng@scsio.ac.cn>, huangwf26 <huangwf26@mail2.sysu.edu.cn>, wangqq36 <wangqq36@mail2.sysu.edu.cn>, caigd3 <caigd3@mail2.sysu.edu.cn>, xiaf6 <xiaf6@mail2.sysu.edu.cn>, zhoushn3 <zhoushn3@mail.sysu.edu.cn>, mash33 <mash33@mail2.sysu.edu.cn>, nieyichu2 <nieyichu2@126.com>, leijp <leijp@mail.sysu.edu.cn>, limin65 <limin65@mail.sysu.edu.cn>, dengwb5 <dengwb5@mail.sysu.edu.cn>, yonghongliu <yonghongliu@scsio.ac.cn>, hanfh <hanfh@mail.sysu.edu.cn> |

I agree to these author list changes.
Guo Yinfeng

------------------ Original ------------------

**From:** "Mail.sysu.edu.cn";
**Date:** 2022年1月28日(星期五) 上午8:01
**To:** "WANG Xiaojuan";
**Cc:** "王军舰"; "zhonglin6"; "yangb23"; "zhangzhh68"; "wangjunfeng"; "郭银锋"; "huangwf26"; "wangqq36"; "caigd3"; "xiaf6"; "zhoushn3"; "mash33"; "nieyichu2"; "leijp"; "limin65"; "dengwb5"; "yonghongliu"; "hanfh";
**Subject:** Re: Confirming co-authors list changes for our manuscript submitted to Cell Death & Disease

I agree to these author list changes

Peiqing Liu

发自我的iPhone

在 2022年1月27日，下午10:42，WANG Xiaojuan <wxj0250@163.com> 写道:

I agree to these author list changes.

Wang Xiaojun

---- Replied Message ----

| | |
|---|---|
| From | 王军舰<wangjj87@mail.sysu.edu.cn> |
| Date | 01/27/2022 17:02 |
| To | zhonglin6<zhonglin6@mail2.sysu.edu.cn>, yangb23<yangb23@mail.sysu.edu.cn>, zhangzhh68<zhangzhh68@mail2.sysu.edu.cn>, wangjunfeng<wangjunfeng@scsio.ac.cn>, wxj0250<wxj0250@163.com>, guoyf36<guoyf36@mail2.sysu.edu.cn>, huangwf26<huangwf26@mail2.sysu.edu.cn>, wangqq36<wangqq36@mail2.sysu.edu.cn>, caigd3<caigd3@mail2.sysu.edu.cn>, xiaf6<xiaf6@mail2.sysu.edu.cn>, zhoushn3<zhoushn3@mail.sysu.edu.cn>, mash33<mash33@mail2.sysu.edu.cn>, nieyichu2<nieyichu2@126.com>, leijp<leijp@mail.sysu.edu.cn>, limin65<limin65@mail.sysu.edu.cn>, |

liupq<liupq@mail.sysu.edu.cn>,
dengwb5<dengwb5@mail.sysu.edu.cn>,
yonghongliu<yonghongliu@scsio.ac.cn>,
hanfh<hanfh@mail.sysu.edu.cn>

Subject Confirming co-authors list changes for our manuscript submitted to Cell Death & Disease

Dear ,

I am very pleased to inform you that our manuscript CDDIS-21-2849RR entitled "Targeting autophagy peptidase ATG4B with a novel natural product inhibitor Azalomycin F4a for advanced gastric cancer" has been provisionally accepted for publication in Cell Death & Disease.

Since we changed author list from our original submission (CDDIS-21-2849: Lin Zhong[a†], Bin Yang[a†], Zhenhua zhang[b†], Xiaojuan Wang[d], Yinfeng Guo[e], Weifeng Huang[e], Qianqian Wang[e], Guodi Cai[e], Fan Xia[e], Shengning Zhou[a], Shuai Ma[a], Yichu Nie[b], Jinping Lei[e], Min Li[e, f], Peiqing Liu[e, f], Wenbin Deng[b], Yonghong Liu[c], Junfeng Wang[c]*, Fanghai Han[a]*, Junjian Wang[e, f]*) to current author list (CDDIS-21-2849RR:Lin Zhong[a†], Bin Yang[a†], Zhenhua zhang[b†], Junfeng Wang[c]*, Xiaojuan Wang[d], Yinfeng Guo[e], Weifeng Huang[e], Qianqian Wang[e], Guodi Cai[e], Fan Xia[e], Shengning Zhou[a], Shuai Ma[a], Yichu Nie[b], Jinping Lei[e], Min Li[e, f], Peiqing Liu[e, f], Wenbin Deng[b], Yonghong Liu[c], Fanghai Han[a]*, Junjian Wang[e, f]*) .

We need all the coauthors to confirm whether they agree to these changes. If you agree to these changes, please reply "I agree to these author list changes" followed by your name, otherwise, please let me know if you have any questions!

Thank you for your help!

Junjian Wang

---

本邮件及其附件含有发送给特定个人和用于特定目的的信息。如果您不是预期的收件人，请立即删除本邮件并通知发件人。严禁任何非预期的收件人使用、传播、分发或复制本邮件或其附件。
This email and its attachments may contain confidential information intended for a specific individual and purpose. If you are not the intended recipient, you should delete this email and notify the sender immediately. Any use, dissemination, distribution, or copying of this email or its attachments by persons other than the intended recipient(s), is strictly prohibited.

---

本邮件及其附件含有发送给特定个人和用于特定目的的信息。如果您不是预期的收件人，请立即删除本邮件并通知发件人。严禁任何非预期的收件人使用、传播、分发或复制本邮件或其附件。
This email and its attachments may contain confidential information intended for a specific individual and purpose. If you are not the intended recipient, you should delete this email and notify the sender immediately. Any use, dissemination, distribution, or copying of this email or its attachments by persons other than the intended recipient(s), is strictly prohibited.

| | |
|---|---|
| 主　题: | Re:Confirming co-authors list changes for our manuscript submitted to Cell Death & Disease |
| 发件人: | "黄炜枫" <huangwf26@mail2.sysu.edu.cn> 2022-1-28 11:14:02 |
| 收件人: | "王军舰" <wangjj87@mail.sysu.edu.cn> |

Dear,

I agree to these author list changes, Thank you!

Best regards

Weifeng Huang

------------------ Original ------------------

**From:** "王军舰"<wangjj87@mail.sysu.edu.cn>;
**Date:** Thu, Jan 27, 2022 05:02 PM
**To:** "zhonglin6"<zhonglin6@mail2.sysu.edu.cn>; "yangb23"<yangb23@mail.sysu.edu.cn>; "zhangzhh68" <zhangzhh68@mail2.sysu.edu.cn>; "wangjunfeng"<wangjunfeng@scsio.ac.cn>; "wxj0250"<wxj0250@163.com>; "guoyf36" <guoyf36@mail2.sysu.edu.cn>; "黄炜枫"<huangwf26@mail2.sysu.edu.cn>; "wangqq36"<wangqq36@mail2.sysu.edu.cn>; "caigd3"<caigd3@mail2.sysu.edu.cn>; "xiaf6"<xiaf6@mail2.sysu.edu.cn>; "zhoushn3"<zhoushn3@mail.sysu.edu.cn>; "mash33"<mash33@mail2.sysu.edu.cn>; "nieyichu2"<nieyichu2@126.com>; "雷金平"<leijp@mail.sysu.edu.cn>; "limin65" <limin65@mail.sysu.edu.cn>; "liupq"<liupq@mail.sysu.edu.cn>; "dengwb5"<dengwb5@mail.sysu.edu.cn>; "yonghongliu" <yonghongliu@scsio.ac.cn>; "hanfh"<hanfh@mail.sysu.edu.cn>;
**Subject:** Confirming co-authors list changes for our manuscript submitted to Cell Death & Disease

Dear ,

I am very pleased to inform you that our manuscript CDDIS-21-2849RR entitled "Targeting autophagy peptidase ATG4B with a novel natural product inhibitor Azalomycin F4a for advanced gastric cancer" has been provisionally accepted for publication in Cell Death & Disease.

Since we changed author list from our original submission (CDDIS-21-2849: Lin Zhong[a†], Bin Yang[a†], Zhenhua zhang[b†], Xiaojuan Wang[d], Yinfeng Guo[e], Weifeng Huang[e], Qianqian Wang[e], Guodi Cai[e], Fan Xia[e], Shengning Zhou[a], Shuai Ma[a], Yichu Nie[b], Jinping Lei[e], Min Li[e, f], Peiqing Liu[e, f], Wenbin Deng[b], Yonghong Liu[c], Junfeng Wang[c*], Fanghai Han[a*], Junjian Wang[e, f*]) to current author list (CDDIS-21-2849RR:Lin Zhong[a†], Bin Yang[a†], Zhenhua zhang[b†], Junfeng Wang[c*], Xiaojuan Wang[d], Yinfeng Guo[e], Weifeng Huang[e], Qianqian Wang[e], Guodi Cai[e], Fan Xia[e], Shengning Zhou[a], Shuai Ma[a], Yichu Nie[b], Jinping Lei[e], Min Li[e, f], Peiqing Liu[e, f], Wenbin Deng[b], Yonghong Liu[c], Fanghai Han[a*], Junjian Wang[e, f*]) .

We need all the coauthors to confirm whether they agree to these changes. If you agree to these changes, please reply “I agree to these author list changes” followed by your name, otherwise, please let me know if you have any questions!

Thank you for your help!

Junjian Wang

---

本邮件及其附件含有发送给特定个人和用于特定目的的信息。如果您不是预期的收件人，请立即删除本邮件并通

知发件人。严禁任何非预期的收件人使用、传播、分发或复制本邮件或其附件。
This email and its attachments may contain confidential information intended for a specific individual and purpose. If you are not the intended recipient, you should delete this email and notify the sender immediately. Any use, dissemination, distribution, or copying of this email or its attachments by persons other than the intended recipient(s), is strictly prohibited.

| | |
|---|---|
| 主　题: | 回复:Confirming co-authors list changes for our manuscript submitted to Cell Death & Disease |
| 发件人: | "王千千" <wangqq36@mail2.sysu.edu.cn> 2022-1-28 10:42:34 |
| 收件人: | "王军舰" <wangjj87@mail.sysu.edu.cn> |

Dear Dr. Wang

I agree to these author list changes, Thank you!

Best regards

Qianqian Wang

--------------原始邮件--------------
发件人："王军舰 "<wangjj87@mail.sysu.edu.cn>;
发送时间：2022年1月27日(星期四) 下午5:03
收件人："zhonglin6" <zhonglin6@mail2.sysu.edu.cn>;"yangb23" <yangb23@mail.sysu.edu.cn>;"zhangzhh68" <zhangzhh68@mail2.sysu.edu.cn>;"wangjunfeng" <wangjunfeng@scsio.ac.cn>;"wxj0250" <wxj0250@163.com>;"guoyf36" <guoyf36@mail2.sysu.edu.cn>;"huangwf26" <huangwf26@mail2.sysu.edu.cn>;"王千千" <wangqq36@mail2.sysu.edu.cn>;"caigd3" <caigd3@mail2.sysu.edu.cn>;"xiaf6" <xiaf6@mail2.sysu.edu.cn>;"zhoushn3" <zhoushn3@mail.sysu.edu.cn>;"mash33" <mash33@mail2.sysu.edu.cn>;"nieyichu2" <nieyichu2@126.com>;"leijp" <leijp@mail.sysu.edu.cn>;"limin65" <limin65@mail.sysu.edu.cn>;"liupq" <liupq@mail.sysu.edu.cn>;"dengwb5" <dengwb5@mail.sysu.edu.cn>;"yonghongliu" <yonghongliu@scsio.ac.cn>;"hanfh" <hanfh@mail.sysu.edu.cn>;
主题：Confirming co-authors list changes for our manuscript submitted to Cell Death & Disease
----------------------------------

Dear ,

I am very pleased to inform you that our manuscript CDDIS-21-2849RR entitled ″Targeting autophagy peptidase ATG4B with a novel natural product inhibitor Azalomycin F4a for advanced gastric cancer″ has been provisionally accepted for publication in Cell Death & Disease.

Since we changed author list from our original submission (CDDIS-21-2849: Lin Zhong[a†], Bin Yang[a†], Zhenhua zhang[b†], Xiaojuan Wang[d], Yinfeng Guo[e], Weifeng Huang[e], Qianqian Wang[e], Guodi Cai[e], Fan Xia[e], Shengning Zhou[a], Shuai Ma[a], Yichu Nie[b], Jinping Lei[e], Min Li[e, f], Peiqing Liu[e, f], Wenbin Deng[b], Yonghong Liu[c], Junfeng Wang[c]*, Fanghai Han[a]*, Junjian Wang[e, f]*) to current author list (CDDIS-21-2849RR:Lin Zhong[a†], Bin Yang[a†], Zhenhua zhang[b†], Junfeng Wang[c]*, Xiaojuan Wang[d], Yinfeng Guo[e], Weifeng Huang[e], Qianqian Wang[e], Guodi Cai[e], Fan Xia[e], Shengning Zhou[a], Shuai Ma[a], Yichu Nie[b], Jinping Lei[e], Min Li[e, f], Peiqing Liu[e, f], Wenbin Deng[b], Yonghong Liu[c], Fanghai Han[a]*, Junjian Wang[e, f]*) .

We need all the coauthors to confirm whether they agree to these changes. If you agree to these changes, please reply “I agree to these author list changes” followed by your name, otherwise, please let me know if you have any questions!

Thank you for your help!

Junjian Wang

本邮件及其附件含有发送给特定个人和用于特定目的的信息。如果您不是预期的收件人，请立即删除本邮件并通知发件人。严禁任何非预期的收件人使用、传播、分发或复制本邮件或其附件。
This email and its attachments may contain confidential information intended for a specific individual and purpose. If you are not the intended recipient, you should delete this email and notify the sender immediately. Any use, dissemination, distribution, or copying of this email or its attachments by persons other than the intended recipient(s), is strictly prohibited.

| | | |
|---|---|---|
| 主　题: | Re: Confirming co-authors list changes for our manuscript submitted to Cell Death & Disease | |
| 发件人: | "蔡国弟" <caigd3@mail2.sysu.edu.cn> | 2022-1-27 17:12:08 |
| 收件人: | "王军舰" <wangjj87@mail.sysu.edu.cn> | |

I agree to these author list changes.

Guodi Cai

---Original---

**From:** "王军舰"<wangjj87@mail.sysu.edu.cn>
**Date:** Thu, Jan 27, 2022 17:02 PM
**To:** "zhonglin6"<zhonglin6@mail2.sysu.edu.cn>;"yangb23"<yangb23@mail.sysu.edu.cn>;"zhangzhh68"<zhangzhh68@mail2.sysu.edu.cn>;"wangjunfeng"<wangjunfeng@scsio.ac.cn>;"wxj0250"<wxj0250@163.com>;"guoyf36"<guoyf36@mail2.sysu.edu.cn>;"huangwf26"<huangwf26@mail2.sysu.edu.cn>;"wangqq36"<wangqq36@mail2.sysu.edu.cn>;"caigd3"<caigd3@mail2.sysu.edu.cn>;"xiaf6"<xiaf6@mail2.sysu.edu.cn>;"zhoushn3"<zhoushn3@mail.sysu.edu.cn>;"mash33"<mash33@mail2.sysu.edu.cn>;"nieyichu2"<nieyichu2@126.com>;"leijp"<leijp@mail.sysu.edu.cn>;"limin65"<limin65@mail.sysu.edu.cn>;"liupq"<liupq@mail.sysu.edu.cn>;"dengwb5"<dengwb5@mail.sysu.edu.cn>;"yonghongliu"<yonghongliu@scsio.ac.cn>;"hanfh"<hanfh@mail.sysu.edu.cn>;
**Subject:** Confirming co-authors list changes for our manuscript submitted to Cell Death & Disease

Dear ,

I am very pleased to inform you that our manuscript CDDIS-21-2849RR entitled "Targeting autophagy peptidase ATG4B with a novel natural product inhibitor Azalomycin F4a for advanced gastric cancer" has been provisionally accepted for publication in Cell Death & Disease.

Since we changed author list from our original submission (CDDIS-21-2849: Lin Zhong[a†], Bin Yang[a†], Zhenhua zhang[b†], Xiaojuan Wang[d], Yinfeng Guo[e], Weifeng Huang[e], Qianqian Wang[e], Guodi Cai[e], Fan Xia[e], Shengning Zhou[a], Shuai Ma[a], Yichu Nie[b], Jinping Lei[e], Min Li[e, f], Peiqing Liu[e, f], Wenbin Deng[b], Yonghong Liu[c], Junfeng Wang[c]*, Fanghai Han[a]*, Junjian Wang[e, f]*) to current author list (CDDIS-21-2849RR:Lin Zhong[a†], Bin Yang[a†], Zhenhua zhang[b†], Junfeng Wang[c]*, Xiaojuan Wang[d], Yinfeng Guo[e], Weifeng Huang[e], Qianqian Wang[e], Guodi Cai[e], Fan Xia[e], Shengning Zhou[a], Shuai Ma[a], Yichu Nie[b], Jinping Lei[e], Min Li[e, f], Peiqing Liu[e, f], Wenbin Deng[b], Yonghong Liu[c], Fanghai Han[a]*, Junjian Wang[e, f]*).

We need all the coauthors to confirm whether they agree to these changes. If you agree to these changes, please reply "I agree to these author list changes" followed by your name, otherwise, please let me know if you have any questions!

Thank you for your help!

Junjian Wang

本邮件及其附件含有发送给特定个人和用于特定目的的信息。如果您不是预期的收件人，请立即删除本邮件并通知发件人。严禁任何非预期的收件人使用、传播、分发或复制本邮件或其附件。
This email and its attachments may contain confidential information intended for a specific individual and purpose. If you are not the intended recipient, you should delete this email and notify the sender immediately. Any use, dissemination, distribution, or copying of this email or its attachments by persons other than the intended recipient(s), is strictly prohibited.

| | | |
|---|---|---|
| 主　题: | 回复:Confirming co-authors list changes for our manuscript submitted to Cell Death & Disease | |
| 发件人: | "夏凡" <xiaf6@mail2.sysu.edu.cn> | 2022-1-27 20:50:04 |
| 收件人: | "王军舰老师" <wangjj87@mail.sysu.edu.cn> | |

Dear,

I agree to these author list changes, Thank you!

Best regards

Fan Xia

--------------原始邮件--------------
发件人: "王军舰老师 "<wangjj87@mail.sysu.edu.cn>;
发送时间: 2022年1月27日(星期四) 下午5:03
收件人: "zhonglin6" <zhonglin6@mail2.sysu.edu.cn>;"yangb23" <yangb23@mail.sysu.edu.cn>;"zhangzhh68" <zhangzhh68@mail2.sysu.edu.cn>;"wangjunfeng" <wangjunfeng@scsio.ac.cn>;"wxj0250" <wxj0250@163.com>;"guoyf36" <guoyf36@mail2.sysu.edu.cn>;"huangwf26" <huangwf26@mail2.sysu.edu.cn>;"wangqq36" <wangqq36@mail2.sysu.edu.cn>;"caigd3" <caigd3@mail2.sysu.edu.cn>;"夏凡" <xiaf6@mail2.sysu.edu.cn>;"zhoushn3" <zhoushn3@mail.sysu.edu.cn>;"mash33" <mash33@mail2.sysu.edu.cn>;"nieyichu2" <nieyichu2@126.com>;"leijp" <leijp@mail.sysu.edu.cn>;"李民老师" <limin65@mail.sysu.edu.cn>;"刘培庆老师" <liupq@mail.sysu.edu.cn>;"dengwb5" <dengwb5@mail.sysu.edu.cn>;"yonghongliu" <yonghongliu@scsio.ac.cn>;"hanfh" <hanfh@mail.sysu.edu.cn>;
主题: Confirming co-authors list changes for our manuscript submitted to Cell Death & Disease
-----------------------------------

Dear ,

I am very pleased to inform you that our manuscript CDDIS-21-2849RR entitled "Targeting autophagy peptidase ATG4B with a novel natural product inhibitor Azalomycin F4a for advanced gastric cancer" has been provisionally accepted for publication in Cell Death & Disease.

Since we changed author list from our original submission (CDDIS-21-2849: Lin Zhong[a†], Bin Yang[a†], Zhenhua zhang[b†], Xiaojuan Wang[d], Yinfeng Guo[e], Weifeng Huang[e], Qianqian Wang[e], Guodi Cai[e], Fan Xia[e], Shengning Zhou[a], Shuai Ma[a], Yichu Nie[b], Jinping Lei[e], Min Li[e, f], Peiqing Liu[e, f], Wenbin Deng[b], Yonghong Liu[c], Junfeng Wang[c]*, Fanghai Han[a]*, Junjian Wang[e, f]*) to current author list (CDDIS-21-2849RR:Lin Zhong[a†], Bin Yang[a†], Zhenhua zhang[b†], Junfeng Wang[c]*, Xiaojuan Wang[d], Yinfeng Guo[e], Weifeng Huang[e], Qianqian Wang[e], Guodi Cai[e], Fan Xia[e], Shengning Zhou[a], Shuai Ma[a], Yichu Nie[b], Jinping Lei[e], Min Li[e, f], Peiqing Liu[e, f], Wenbin Deng[b], Yonghong Liu[c], Fanghai Han[a]*, Junjian Wang[e, f]*).

We need all the coauthors to confirm whether they agree to these changes. If you agree to these changes, please reply "I agree to these author list changes" followed by your name, otherwise, please let me know if you have any questions!

Thank you for your help!

Junjian Wang

---

本邮件及其附件含有发送给特定个人和用于特定目的的信息。如果您不是预期的收件人，请立即删除本邮件并通知发件人。严禁任何非预期的收件人使用、传播、分发或复制本邮件或其附件。
This email and its attachments may contain confidential information intended for a specific individual and purpose. If you are not the intended recipient, you should delete this email and notify the sender immediately.

Any use, dissemination, distribution, or copying of this email or its attachments by persons other than the intended recipient(s), is strictly prohibited.

| | | |
|---|---|---|
| 主　题: | Re: Confirming co-authors list changes for our manuscript submitted to Cell Death & Disease | |
| 发件人: | "周声宁" <zhoushn3@mail.sysu.edu.cn> | 2022-1-28 8:31:41 |
| 收件人: | "王军舰" <wangjj87@mail.sysu.edu.cn> | |

I agree to these author list changes,  ShengningZhou

-----原始邮件-----
**发件人:**"王军舰" <wangjj87@mail.sysu.edu.cn>
**发送时间:**2022-01-27 17:02:54 (星期四)
**收件人:** zhonglin6@mail2.sysu.edu.cn, yangb23@mail.sysu.edu.cn, zhangzhh68@mail2.sysu.edu.cn, wangjunfeng@scsio.ac.cn, wxj0250@163.com, guoyf36@mail2.sysu.edu.cn, huangwf26@mail2.sysu.edu.cn, wangqq36@mail2.sysu.edu.cn, caigd3@mail2.sysu.edu.cn, xiaf6@mail2.sysu.edu.cn, zhoushn3@mail.sysu.edu.cn, mash33@mail2.sysu.edu.cn, nieyichu2@126.com, leijp@mail.sysu.edu.cn, limin65@mail.sysu.edu.cn, liupq@mail.sysu.edu.cn, dengwb5@mail.sysu.edu.cn, yonghongliu@scsio.ac.cn, hanfh@mail.sysu.edu.cn
**抄送:**
**主题:** Confirming co-authors list changes for our manuscript submitted to Cell Death & Disease

Dear ,

I am very pleased to inform you that our manuscript CDDIS-21-2849RR entitled "Targeting autophagy peptidase ATG4B with a novel natural product inhibitor Azalomycin F4a for advanced gastric cancer" has been provisionally accepted for publication in Cell Death & Disease.

Since we changed author list from our original submission (CDDIS-21-2849: Lin Zhong[a†], Bin Yang[a†], Zhenhua zhang[b†], Xiaojuan Wang[d], Yinfeng Guo[e], Weifeng Huang[e], Qianqian Wang[e], Guodi Cai[e], Fan Xia[e], Shengning Zhou[a], Shuai Ma[a], Yichu Nie[b], Jinping Lei[e], Min Li[e, f], Peiqing Liu[e, f], Wenbin Deng[b], Yonghong Liu[c], Junfeng Wang[c]*, Fanghai Han[a]*, Junjian Wang[e, f]*) to current author list (CDDIS-21-2849RR:Lin Zhong[a†], Bin Yang[a†], Zhenhua zhang[b†], Junfeng Wang[c]*, Xiaojuan Wang[d], Yinfeng Guo[e], Weifeng Huang[e], Qianqian Wang[e], Guodi Cai[e], Fan Xia[e], Shengning Zhou[a], Shuai Ma[a], Yichu Nie[b], Jinping Lei[e], Min Li[e, f], Peiqing Liu[e, f], Wenbin Deng[b], Yonghong Liu[c], Fanghai Han[a]*, Junjian Wang[e, f]*) .

We need all the coauthors to confirm whether they agree to these changes. If you agree to these changes, please reply "I agree to these author list changes" followed by your name, otherwise, please let me know if you have any questions!

Thank you for your help!

Junjian Wang

本邮件及其附件含有发送给特定个人和用于特定目的的信息。如果您不是预期的收件人，请立即删除本邮件并通知发件人。严禁任何非预期的收件人使用、传播、分发或复制本邮件或其附件。
This email and its attachments may contain confidential information intended for a specific individual and purpose. If you are not the intended recipient, you should delete this email and notify the sender immediately. Any use, dissemination, distribution, or copying of this email or its attachments by persons other than the intended recipient(s), is strictly prohibited.

本邮件及其附件含有发送给特定个人和用于特定目的的信息。如果您不是预期的收件人，请立即删除本邮件并通知发件人。严禁任何非预期的收件人使用、传播、分发或复制本邮件或其附件。
This email and its attachments may contain confidential information intended for a specific individual and purpose. If you are not the intended recipient, you should delete this email and notify the sender immediately. Any use, dissemination, distribution, or copying of this email or its attachments by persons other than the intended recipient(s), is strictly prohibited.

| | | |
|---|---|---|
| 主　题: | 回复:Confirming co-authors list changes for our manuscript submitted to Cell Death & Disease | |
| 发件人: | "马帅" <mash33@mail2.sysu.edu.cn> | 2022-1-27 17:09:19 |
| 收件人: | "王军舰" <wangjj87@mail.sysu.edu.cn> | |

I agree to these author list changes
Shuai Ma

----------

该邮件从移动设备发送

--------------原始邮件--------------
发件人："王军舰 "<wangjj87@mail.sysu.edu.cn>;
发送时间：2022年1月27日(星期四) 下午5:03
收件人："zhonglin6" <zhonglin6@mail2.sysu.edu.cn>;"yangb23" <yangb23@mail.sysu.edu.cn>;"zhangzhh68" <zhangzhh68@mail2.sysu.edu.cn>;"wangjunfeng" <wangjunfeng@scsio.ac.cn>;"wxj0250" <wxj0250@163.com>;"guoyf36" <guoyf36@mail2.sysu.edu.cn>;"huangwf26" <huangwf26@mail2.sysu.edu.cn>;"wangqq36" <wangqq36@mail2.sysu.edu.cn>;"caigd3" <caigd3@mail2.sysu.edu.cn>;"xiaf6" <xiaf6@mail2.sysu.edu.cn>;"zhoushn3" <zhoushn3@mail.sysu.edu.cn>;"马帅" <mash33@mail2.sysu.edu.cn>;"nieyichu2" <nieyichu2@126.com>;"leijp" <leijp@mail.sysu.edu.cn>;"limin65" <limin65@mail.sysu.edu.cn>;"liupq" <liupq@mail.sysu.edu.cn>;"dengwb5" <dengwb5@mail.sysu.edu.cn>;"yonghongliu" <yonghongliu@scsio.ac.cn>;"hanfh" <hanfh@mail.sysu.edu.cn>;
主题：Confirming co-authors list changes for our manuscript submitted to Cell Death & Disease
----------------------------------

Dear ,

I am very pleased to inform you that our manuscript CDDIS-21-2849RR entitled "Targeting autophagy peptidase ATG4B with a novel natural product inhibitor Azalomycin F4a for advanced gastric cancer" has been provisionally accepted for publication in Cell Death & Disease.

Since we changed author list from our original submission (CDDIS-21-2849: Lin Zhong[a†], Bin Yang[a†], Zhenhua zhang[b†], Xiaojuan Wang[d], Yinfeng Guo[e], Weifeng Huang[e], Qianqian Wang[e], Guodi Cai[e], Fan Xia[e], Shengning Zhou[a], Shuai Ma[a], Yichu Nie[b], Jinping Lei[e], Min Li[e, f], Peiqing Liu[e, f], Wenbin Deng[b], Yonghong Liu[c], Junfeng Wang[c*], Fanghai Han[a*], Junjian Wang[e, f*]) to current author list (CDDIS-21-2849RR:Lin Zhong[a†], Bin Yang[a†], Zhenhua zhang[b†], Junfeng Wang[c*], Xiaojuan Wang[d], Yinfeng Guo[e], Weifeng Huang[e], Qianqian Wang[e], Guodi Cai[e], Fan Xia[e], Shengning Zhou[a], Shuai Ma[a], Yichu Nie[b], Jinping Lei[e], Min Li[e, f], Peiqing Liu[e, f], Wenbin Deng[b], Yonghong Liu[c], Fanghai Han[a*], Junjian Wang[e, f*]).

We need all the coauthors to confirm whether they agree to these changes. If you agree to these changes, please reply "I agree to these author list changes" followed by your name, otherwise, please let me know if you have any questions!

Thank you for your help!

Junjian Wang

本邮件及其附件含有发送给特定个人和用于特定目的的信息。如果您不是预期的收件人，请立即删除本邮件并通知发件人。严禁任何非预期的收件人使用、传播、分发或复制本邮件或其附件。
This email and its attachments may contain confidential information intended for a specific individual and

purpose. If you are not the intended recipient, you should delete this email and notify the sender immediately. Any use, dissemination, distribution, or copying of this email or its attachments by persons other than the intended recipient(s), is strictly prohibited.

| 主　题: | Re:Confirming co-authors list changes for our manuscript submitted to Cell Death & Disease | |
|---|---|---|
| 发件人: | nieyichu2 <15902069440@126.com> | 2022-1-27 18:20:00 |
| 收件人: | "王军舰" <wangjj87@mail.sysu.edu.cn> | |

I agree to these author list changes.

Best wishes and happy new year.

Yichu Nie

在 2022-01-27 17:02:54，"王军舰" <wangjj87@mail.sysu.edu.cn> 写道：

> Dear ，
>
> I am very pleased to inform you that our manuscript CDDIS-21-2849RR entitled "Targeting autophagy peptidase ATG4B with a novel natural product inhibitor Azalomycin F4a for advanced gastric cancer" has been provisionally accepted for publication in Cell Death & Disease.
>
> Since we changed author list from our original submission (CDDIS-21-2849: Lin Zhong[a†], Bin Yang[a†], Zhenhua zhang[b†], Xiaojuan Wang[d], Yinfeng Guo[e], Weifeng Huang[e], Qianqian Wang[e], Guodi Cai[e], Fan Xia[e], Shengning Zhou[a], Shuai Ma[a], Yichu Nie[b], Jinping Lei[e], Min Li[e, f], Peiqing Liu[e, f], Wenbin Deng[b], Yonghong Liu[c], Junfeng Wang[c*], Fanghai Han[a*], Junjian Wang[e, f*]) to current author list (CDDIS-21-2849RR:Lin Zhong[a†], Bin Yang[a†], Zhenhua zhang[b†], Junfeng Wang[c*], Xiaojuan Wang[d], Yinfeng Guo[e], Weifeng Huang[e], Qianqian Wang[e], Guodi Cai[e], Fan Xia[e], Shengning Zhou[a], Shuai Ma[a], Yichu Nie[b], Jinping Lei[e], Min Li[e, f], Peiqing Liu[e, f], Wenbin Deng[b], Yonghong Liu[c], Fanghai Han[a*], Junjian Wang[e, f*]) .
>
> We need all the coauthors to confirm whether they agree to these changes. If you agree to these changes, please reply "I agree to these author list changes" followed by your name, otherwise, please let me know if you have any questions!
>
> Thank you for your help!
>
> Junjian Wang

本邮件及其附件含有发送给特定个人和用于特定目的的信息。如果您不是预期的收件人，请立即删除本邮件并通知发件人。严禁任何非预期的收件人使用、传播、分发或复制本邮件或其附件。

This email and its attachments may contain confidential information intended for a specific individual and purpose. If you are not the intended recipient, you should delete this email and notify the sender immediately. Any use, dissemination, distribution, or copying of this email or its attachments by persons other than the intended recipient(s), is strictly prohibited.

| | |
|---|---|
| 主　题: | Re: Confirming co-authors list changes for our manuscript submitted to Cell Death & Disease |
| 发件人: | "雷金平" <leijp@mail.sysu.edu.cn> 2022-1-27 20:26:17 |
| 收件人: | "王军舰" <wangjj87@mail.sysu.edu.cn> |
| 抄　送: | zhonglin6@mail2.sysu.edu.cn, yangb23@mail.sysu.edu.cn, zhangzhh68@mail2.sysu.edu.cn, wangjunfeng@scsio.ac.cn, wxj0250@163.com, guoyf36@mail2.sysu.edu.cn, huangwf26@mail2.sysu.edu.cn, wangqq36@mail2.sysu.edu.cn, caigd3@mail2.sysu.edu.cn, xiaf6@mail2.sysu.edu.cn, zhoushn3@mail.sysu.edu.cn, mash33@mail2.sysu.edu.cn, nieyichu2@126.com, limin65@mail.sysu.edu.cn, liupq@mail.sysu.edu.cn, dengwb5@mail.sysu.edu.cn, yonghongliu@scsio.ac.cn, hanfh@mail.sysu.edu.cn |

Dear,

I agree to these author list changes, Thank you!

Best regards

Jinping Lei

-----原始邮件-----
**发件人:**"王军舰" <wangjj87@mail.sysu.edu.cn>
**发送时间:**2022-01-27 17:02:54 (星期四)
**收件人:** zhonglin6@mail2.sysu.edu.cn, yangb23@mail.sysu.edu.cn, zhangzhh68@mail2.sysu.edu.cn, wangjunfeng@scsio.ac.cn, wxj0250@163.com, guoyf36@mail2.sysu.edu.cn, huangwf26@mail2.sysu.edu.cn, wangqq36@mail2.sysu.edu.cn, caigd3@mail2.sysu.edu.cn, xiaf6@mail2.sysu.edu.cn, zhoushn3@mail.sysu.edu.cn, mash33@mail2.sysu.edu.cn, nieyichu2@126.com, leijp@mail.sysu.edu.cn, limin65@mail.sysu.edu.cn, liupq@mail.sysu.edu.cn, dengwb5@mail.sysu.edu.cn, yonghongliu@scsio.ac.cn, hanfh@mail.sysu.edu.cn
**抄送:**
**主题:** Confirming co-authors list changes for our manuscript submitted to Cell Death & Disease

Dear ,

I am very pleased to inform you that our manuscript CDDIS-21-2849RR entitled "Targeting autophagy peptidase ATG4B with a novel natural product inhibitor Azalomycin F4a for advanced gastric cancer" has been provisionally accepted for publication in Cell Death & Disease.

Since we changed author list from our original submission (CDDIS-21-2849: Lin Zhong[a†], Bin Yang[a†], Zhenhua zhang[b†], Xiaojuan Wang[d], Yinfeng Guo[e], Weifeng Huang[e], Qianqian Wang[e], Guodi Cai[e], Fan Xia[e], Shengning Zhou[a], Shuai Ma[a], Yichu Nie[b], Jinping Lei[e], Min Li[e, f], Peiqing Liu[e, f], Wenbin Deng[b], Yonghong Liu[c], Junfeng Wang[c]*, Fanghai Han[a]*, Junjian Wang[e, f]*) to current author list (CDDIS-21-2849RR:Lin Zhong[a†], Bin Yang[a†], Zhenhua zhang[b†], Junfeng Wang[c]*, Xiaojuan Wang[d], Yinfeng Guo[e], Weifeng Huang[e], Qianqian Wang[e], Guodi Cai[e], Fan Xia[e], Shengning Zhou[a], Shuai Ma[a], Yichu Nie[b], Jinping Lei[e], Min Li[e, f], Peiqing Liu[e, f], Wenbin Deng[b], Yonghong Liu[c], Fanghai Han[a]*, Junjian Wang[e, f]*) .

We need all the coauthors to confirm whether they agree to these changes. If you agree to these changes, please reply "I agree to these author list changes" followed by your name, otherwise, please let me know if you have any questions!

Thank you for your help!

Junjian Wang

---

本邮件及其附件含有发送给特定个人和用于特定目的的信息。如果您不是预期的收件人，请立即删除本邮件并通知发件人。严禁任何非预期的收件人使用、传播、分发或复制本邮件或其附件。
This email and its attachments may contain confidential information intended for a specific individual and purpose. If you are not the intended recipient, you should delete this email and notify the sender immediately. Any use, dissemination, distribution, or copying of this email or its attachments by persons other than the intended recipient(s), is strictly prohibited.

---

本邮件及其附件含有发送给特定个人和用于特定目的的信息。如果您不是预期的收件人，请立即删除本邮件并通知发件人。严禁任何非预期的收件人使用、传播、分发或复制本邮件或其附件。
This email and its attachments may contain confidential information intended for a specific individual and purpose. If you are not the intended recipient, you should delete this email and notify the sender immediately. Any use, dissemination, distribution, or copying of this email or its attachments by persons other than the intended recipient(s), is strictly prohibited.

| 主　题: | 回复：Confirming co-authors list changes for our manuscript submitted to Cell Death & Disease | |
|---|---|---|
| 发件人: | "limin65@mail.sysu.edu.cn" <limin65@mail.sysu.edu.cn> | 2022-1-27 18:17:57 |
| 收件人: | "王军舰" <wangjj87@mail.sysu.edu.cn> | |

Dear JJ,

I agree to these author list changes. Thanks

Min Li

发自我的华为手机

-------- 原始邮件 --------
发件人： 王军舰 <wangjj87@mail.sysu.edu.cn>
日期： 2022年1月27日周四 17:02
收件人： zhonglin6@mail2.sysu.edu.cn, yangb23@mail.sysu.edu.cn, zhangzhh68@mail2.sysu.edu.cn, wangjunfeng@scsio.ac.cn, wxj0250@163.com, guoyf36@mail2.sysu.edu.cn, huangwf26@mail2.sysu.edu.cn, wangqq36@mail2.sysu.edu.cn, caigd3@mail2.sysu.edu.cn, xiaf6@mail2.sysu.edu.cn, zhoushn3@mail.sysu.edu.cn, mash33@mail2.sysu.edu.cn, nieyichu2@126.com, leijp@mail.sysu.edu.cn, limin65@mail.sysu.edu.cn, liupq@mail.sysu.edu.cn, dengwb5@mail.sysu.edu.cn, yonghongliu@scsio.ac.cn, hanfh@mail.sysu.edu.cn
主 题： Confirming co-authors list changes for our manuscript submitted to Cell Death & Disease

Dear ,

I am very pleased to inform you that our manuscript CDDIS-21-2849RR entitled "Targeting autophagy peptidase ATG4B with a novel natural product inhibitor Azalomycin F4a for advanced gastric cancer" has been provisionally accepted for publication in Cell Death & Disease.

Since we changed author list from our original submission (CDDIS-21-2849: Lin Zhong[a†], Bin Yang[a†], Zhenhua zhang[b†], Xiaojuan Wang[d], Yinfeng Guo[e], Weifeng Huang[e], Qianqian Wang[e], Guodi Cai[e], Fan Xia[e], Shengning Zhou[a], Shuai Ma[a], Yichu Nie[b], Jinping Lei[e], Min Li[e, f], Peiqing Liu[e, f], Wenbin Deng[b], Yonghong Liu[c], Junfeng Wang[c*], Fanghai Han[a*], Junjian Wang[e, f*]) to current author list (CDDIS-21-2849RR:Lin Zhong[a†], Bin Yang[a†], Zhenhua zhang[b†], Junfeng Wang[c*], Xiaojuan Wang[d], Yinfeng Guo[e], Weifeng Huang[e], Qianqian Wang[e], Guodi Cai[e], Fan Xia[e], Shengning Zhou[a], Shuai Ma[a], Yichu Nie[b], Jinping Lei[e], Min Li[e, f], Peiqing Liu[e, f], Wenbin Deng[b], Yonghong Liu[c], Fanghai Han[a*], Junjian Wang[e, f*]).

We need all the coauthors to confirm whether they agree to these changes. If you agree to these changes, please reply "I agree to these author list changes" followed by your name, otherwise, please let me know if you have any questions!

Thank you for your help!

Junjian Wang

本邮件及其附件含有发送给特定个人和用于特定目的的信息。如果您不是预期的收件人，请立即删除本邮件并通知发件人。严禁任何非预期的收件人使用、传播、分发或复制本邮件或其附件。
This email and its attachments may contain confidential information intended for a specific individual and purpose. If you are not the intended recipient, you should delete this email and notify the sender immediately. Any use, dissemination, distribution, or copying of this email or its attachments by persons other than the intended recipient(s), is strictly prohibited.

| | |
|---|---|
| 主　题: | Re: Confirming co-authors list changes for our manuscript submitted to Cell Death & Disease |
| 发件人: | "Mail.sysu.edu.cn" <liupq@mail.sysu.edu.cn> 2022-1-28 8:03:48 |
| 收件人: | "WANG Xiaojuan" <wxj0250@163.com> |
| 抄　送: | "王军舰" <wangjj87@mail.sysu.edu.cn>, zhonglin6 <zhonglin6@mail2.sysu.edu.cn>, yangb23 <yangb23@mail.sysu.edu.cn>, zhangzhh68 <zhangzhh68@mail2.sysu.edu.cn>, wangjunfeng <wangjunfeng@scsio.ac.cn>, guoyf36 <guoyf36@mail2.sysu.edu.cn>, huangwf26 <huangwf26@mail2.sysu.edu.cn>, wangqq36 <wangqq36@mail2.sysu.edu.cn>, caigd3 <caigd3@mail2.sysu.edu.cn>, xiaf6 <xiaf6@mail2.sysu.edu.cn>, zhoushn3 <zhoushn3@mail.sysu.edu.cn>, mash33 <mash33@mail2.sysu.edu.cn>, nieyichu2 <nieyichu2@126.com>, leijp <leijp@mail.sysu.edu.cn>, limin65 <limin65@mail.sysu.edu.cn>, dengwb5 <dengwb5@mail.sysu.edu.cn>, yonghongliu <yonghongliu@scsio.ac.cn>, hanfh <hanfh@mail.sysu.edu.cn> |

Dear Dr. Wang

I agree to these author list changes, Thank you!

Best regards

Peiqing Liu

发自我的iPhone

在 2022年1月27日，下午10:44，WANG Xiaojuan <wxj0250@163.com> 写道：

Dear Dr. Wang,

I agree to these author list changes.

Kinds,
Wang Xiaojuan

---- Replied Message ----

| | |
|---|---|
| From | 王军舰<wangjj87@mail.sysu.edu.cn> |
| Date | 01/27/2022 17:02 |
| To | zhonglin6<zhonglin6@mail2.sysu.edu.cn>, yangb23<yangb23@mail.sysu.edu.cn>, zhangzhh68<zhangzhh68@mail2.sysu.edu.cn>, wangjunfeng<wangjunfeng@scsio.ac.cn>, wxj0250<wxj0250@163.com>, guoyf36<guoyf36@mail2.sysu.edu.cn>, huangwf26<huangwf26@mail2.sysu.edu.cn>, wangqq36<wangqq36@mail2.sysu.edu.cn>, caigd3<caigd3@mail2.sysu.edu.cn>, xiaf6<xiaf6@mail2.sysu.edu.cn>, zhoushn3<zhoushn3@mail.sysu.edu.cn>, mash33<mash33@mail2.sysu.edu.cn>, nieyichu2<nieyichu2@126.com>, leijp<leijp@mail.sysu.edu.cn>, limin65<limin65@mail.sysu.edu.cn>, liupq<liupq@mail.sysu.edu.cn>, dengwb5<dengwb5@mail.sysu.edu.cn>, yonghongliu<yonghongliu@scsio.ac.cn>, hanfh<hanfh@mail.sysu.edu.cn> |
| Subject | Confirming co-authors list changes for our manuscript submitted to Cell Death & Disease |

Dear ,

I am very pleased to inform you that our manuscript CDDIS-21-2849RR entitled "Targeting autophagy peptidase ATG4B with a novel natural product inhibitor Azalomycin F4a for advanced gastric cancer" has been provisionally accepted for publication in Cell Death & Disease.

Since we changed author list from our original submission (CDDIS-21-2849: Lin Zhong[a†], Bin Yang[a†], Zhenhua zhang[b†], Xiaojuan Wang[d], Yinfeng Guo[e], Weifeng Huang[e], Qianqian Wang[e], Guodi Cai[e], Fan Xia[e], Shengning Zhou[a], Shuai Ma[a], Yichu Nie[b], Jinping Lei[e], Min Li[e, f], Peiqing Liu[e, f], Wenbin Deng[b], Yonghong Liu[c], Junfeng Wang[c]*, Fanghai Han[a]*, Junjian Wang[e, f]*) to current author list (CDDIS-21-2849RR:Lin Zhong[a†], Bin Yang[a†], Zhenhua zhang[b†], Junfeng Wang[c]*, Xiaojuan Wang[d], Yinfeng Guo[e], Weifeng Huang[e], Qianqian Wang[e], Guodi Cai[e], Fan Xia[e], Shengning Zhou[a], Shuai Ma[a], Yichu Nie[b], Jinping Lei[e], Min Li[e, f], Peiqing Liu[e, f], Wenbin Deng[b], Yonghong Liu[c], Fanghai Han[a]*, Junjian Wang[e, f]*) .

We need all the coauthors to confirm whether they agree to these changes. If you agree to these changes, please reply "I agree to these author list changes" followed by your name, otherwise, please let me know if you have any questions!

Thank you for your help!

Junjian Wang

---

本邮件及其附件含有发送给特定个人和用于特定目的的信息。如果您不是预期的收件人，请立即删除本邮件并通知发件人。严禁任何非预期的收件人使用、传播、分发或复制本邮件或其附件。
This email and its attachments may contain confidential information intended for a specific individual and purpose. If you are not the intended recipient, you should delete this email and notify the sender immediately. Any use, dissemination, distribution, or copying of this email or its attachments by persons other than the intended recipient(s), is strictly prohibited.

---

本邮件及其附件含有发送给特定个人和用于特定目的的信息。如果您不是预期的收件人，请立即删除本邮件并通知发件人。严禁任何非预期的收件人使用、传播、分发或复制本邮件或其附件。
This email and its attachments may contain confidential information intended for a specific individual and purpose. If you are not the intended recipient, you should delete this email and notify the sender immediately. Any use, dissemination, distribution, or copying of this email or its attachments by persons other than the intended recipient(s), is strictly prohibited.

| | | |
|---|---|---|
| 主　题: | Re: Confirming co-authors list changes for our manuscript submitted to Cell Death & Disease | |
| 发件人: | "邓文斌" <dengwb5@mail.sysu.edu.cn> | 2022-1-28 12:59:39 |
| 收件人: | "王军舰" <wangjj87@mail.sysu.edu.cn> | |

I agree to these author list changes. Wenbin Deng

-----原始邮件-----
**发件人:**"王军舰" <wangjj87@mail.sysu.edu.cn>
**发送时间:**2022-01-28 12:50:23 (星期五)
**收件人:** dengwb5@mail.sysu.edu.cn
**抄送:**
**主题:** Confirming co-authors list changes for our manuscript submitted to Cell Death & Disease

Dear Professor Deng,

I am very pleased to inform you that our manuscript CDDIS-21-2849RR entitled "Targeting autophagy peptidase ATG4B with a novel natural product inhibitor Azalomycin F4a for advanced gastric cancer" has been provisionally accepted for publication in Cell Death & Disease.

Since we changed author list from our original submission (CDDIS-21-2849: *Lin Zhong*[a†], *Bin Yang*[a†], *Zhenhua zhang*[b†], *Xiaojuan Wang*[d], *Yinfeng Guo*[e], *Weifeng Huang*[e], *Qianqian Wang*[e], *Guodi Cai*[e], *Fan Xia*[e], *Shengning Zhou*[a], *Shuai Ma*[a], *Yichu Nie*[b], *Jinping Lei*[e], *Min Li*[e, f], *Peiqing Liu*[e, f], *Wenbin Deng*[b], *Yonghong Liu*[c], ***Junfeng Wang***[c*], *Fanghai Han*[a*], *Junjian Wang*[e, f*]) to current author list (CDDIS-21-2849RR: *Lin Zhong*[a†], *Bin Yang*[a†], *Zhenhua zhang*[b†], ***Junfeng Wang***[c*], *Xiaojuan Wang*[d], *Yinfeng Guo*[e], *Weifeng Huang*[e], *Qianqian Wang*[e], *Guodi Cai*[e], *Fan Xia*[e], *Shengning Zhou*[a], *Shuai Ma*[a], *Yichu Nie*[b], *Jinping Lei*[e], *Min Li*[e, f], *Peiqing Liu*[e, f], *Wenbin Deng*[b], *Yonghong Liu*[c], *Fanghai Han*[a*], *Junjian Wang*[e, f*]) .

We need all the coauthors to confirm whether they agree to these changes. **If you agree to these changes, please reply "I agree to these author list changes" followed by your name,** otherwise, please let me know if you have any questions!

Thank you for your help!

Junjian Wang

本邮件及其附件含有发送给特定个人和用于特定目的的信息。如果您不是预期的收件人，请立即删除本邮件并通知发件人。严禁任何非预期的收件人使用、传播、分发或复制本邮件或其附件。
This email and its attachments may contain confidential information intended for a specific individual and purpose. If you are not the intended recipient, you should delete this email and notify the sender immediately. Any use, dissemination, distribution, or copying of this email or its attachments by persons other than the intended recipient(s), is strictly prohibited.

本邮件及其附件含有发送给特定个人和用于特定目的的信息。如果您不是预期的收件人，请立即删除本邮件并通知发件人。严禁任何非预期的收件人使用、传播、分发或复制本邮件或其附件。
This email and its attachments may contain confidential information intended for a specific individual and purpose. If you are not the intended recipient, you should delete this email and notify the sender immediately.

Any use, dissemination, distribution, or copying of this email or its attachments by persons other than the intended recipient(s), is strictly prohibited.

| | | |
|---|---|---|
| 主　题: | Re: Confirming co-authors list changes for our manuscript submitted to Cell Death & Disease | |
| 发件人: | "Yonghong Liu" <yonghongliu@scsio.ac.cn> | 2022-1-27 20:32:02 |
| 收件人: | "王军舰" <wangjj87@mail.sysu.edu.cn> | |

Dear Professor Wang,
**I agree to these author list changes.**

**Sincerely yours,**

**Yonghong Liu**

-----原始邮件-----
**发件人:**"王军舰" <wangjj87@mail.sysu.edu.cn>
**发送时间:**2022-01-27 17:02:54 (星期四)
**收件人:** zhonglin6@mail2.sysu.edu.cn, yangb23@mail.sysu.edu.cn, zhangzhh68@mail2.sysu.edu.cn, wangjunfeng@scsio.ac.cn, wxj0250@163.com, guoyf36@mail2.sysu.edu.cn, huangwf26@mail2.sysu.edu.cn, wangqq36@mail2.sysu.edu.cn, caigd3@mail2.sysu.edu.cn, xiaf6@mail2.sysu.edu.cn, zhoushn3@mail.sysu.edu.cn, mash33@mail2.sysu.edu.cn, nieyichu2@126.com, leijp@mail.sysu.edu.cn, limin65@mail.sysu.edu.cn, liupq@mail.sysu.edu.cn, dengwb5@mail.sysu.edu.cn, yonghongliu@scsio.ac.cn, hanfh@mail.sysu.edu.cn
**抄送:**
**主题:** Confirming co-authors list changes for our manuscript submitted to Cell Death & Disease

Dear ,

I am very pleased to inform you that our manuscript CDDIS-21-2849RR entitled "Targeting autophagy peptidase ATG4B with a novel natural product inhibitor Azalomycin F4a for advanced gastric cancer" has been provisionally accepted for publication in Cell Death & Disease.

Since we changed author list from our original submission (CDDIS-21-2849: Lin Zhong[a†], Bin Yang[a†], Zhenhua zhang[b†], Xiaojuan Wang[d], Yinfeng Guo[e], Weifeng Huang[e], Qianqian Wang[e], Guodi Cai[e], Fan Xia[e], Shengning Zhou[a], Shuai Ma[a], Yichu Nie[b], Jinping Lei[e], Min Li[e, f], Peiqing Liu[e, f], Wenbin Deng[b], Yonghong Liu[c], Junfeng Wang[c]*, Fanghai Han[a]*, Junjian Wang[e, f]*) to current author list (CDDIS-21-2849RR:Lin Zhong[a†], Bin Yang[a†], Zhenhua zhang[b†], Junfeng Wang[c]*, Xiaojuan Wang[d], Yinfeng Guo[e], Weifeng Huang[e], Qianqian Wang[e], Guodi Cai[e], Fan Xia[e], Shengning Zhou[a], Shuai Ma[a], Yichu Nie[b], Jinping Lei[e], Min Li[e, f], Peiqing Liu[e, f], Wenbin Deng[b], Yonghong Liu[c], Fanghai Han[a]*, Junjian Wang[e, f]*) .

We need all the coauthors to confirm whether they agree to these changes. If you agree to these changes, please reply "I agree to these author list changes" followed by your name, otherwise, please let me know if you have any questions!

Thank you for your help!

Junjian Wang

本邮件及其附件含有发送给特定个人和用于特定目的的信息。如果您不是预期的收件人，请立即删除本邮件并通知发件人。严禁任何非预期的收件人使用、传播、分发或复制本邮件或其附件。
This email and its attachments may contain confidential information intended for a specific individual and purpose. If you are not the intended recipient, you should delete this email and notify the sender immediately. Any use, dissemination, distribution, or copying of this email or its attachments by persons other than the intended recipient(s), is strictly prohibited.

致

礼!

刘永宏
广东省海洋药物重点实验室
中国科学院热带海洋生物资源与生态重点实验室
中国科学院海洋微生物中心
中国科学院南海海洋研究所
广州市新港西路164号，广州510301
http://sourcedb.scsio.cas.cn/cn/rck/yjy/201811/t20181115_5178785.html
http://sourcedb.scsio.cas.cn/cn/rck/brjh/200907/t20090716_2086066.html

Sincerely yours,

Yonghong Liu
Guangdong Key Laboratory of Marine Materia Medica
CAS Key Laboratory of Tropical Marine Bio-resources and Ecology
RNAM Center for Marine Microbiology
South China Sea Institute of Oceanology
Chinese Academy of Sciences
No.164 West Xingang Road,Guangzhou 510-301, China
Tel: 0086-20-89023244(O)
http://lmb.scsio.ac.cn/teamshow.asp?action=74
http://sourcedb.scsio.cas.cn/cn/rck/brjh/200907/t20090716_2086066.html
https://www.researchgate.net/profile/Yonghong_Liu4/?ev=hdr_xprf

| 主　题: | Re: Confirming co-authors list changes for our manuscript submitted to Cell Death & Disease | |
|---|---|---|
| 发件人: | "韩方海" <hanfh@mail.sysu.edu.cn> | 2022-1-28 8:37:01 |
| 收件人: | "王军舰" <wangjj87@mail.sysu.edu.cn> | |

Dear Dr. Wang

I agree to these author list changes, Thank you!

Best regards

Fanghai Han

-----原始邮件-----
**发件人:**"王军舰" <wangjj87@mail.sysu.edu.cn>
**发送时间:**2022-01-27 17:02:54 (星期四)
**收件人:** zhonglin6@mail2.sysu.edu.cn, yangb23@mail.sysu.edu.cn, zhangzhh68@mail2.sysu.edu.cn, wangjunfeng@scsio.ac.cn, wxj0250@163.com, guoyf36@mail2.sysu.edu.cn, huangwf26@mail2.sysu.edu.cn, wangqq36@mail2.sysu.edu.cn, caigd3@mail2.sysu.edu.cn, xiaf6@mail2.sysu.edu.cn, zhoushn3@mail.sysu.edu.cn, mash33@mail2.sysu.edu.cn, nieyichu2@126.com, leijp@mail.sysu.edu.cn, limin65@mail.sysu.edu.cn, liupq@mail.sysu.edu.cn, dengwb5@mail.sysu.edu.cn, yonghongliu@scsio.ac.cn, hanfh@mail.sysu.edu.cn
**抄送:**
**主题:** Confirming co-authors list changes for our manuscript submitted to Cell Death & Disease

Dear ,

I am very pleased to inform you that our manuscript CDDIS-21-2849RR entitled "Targeting autophagy peptidase ATG4B with a novel natural product inhibitor Azalomycin F4a for advanced gastric cancer" has been provisionally accepted for publication in Cell Death & Disease.

Since we changed author list from our original submission (CDDIS-21-2849: Lin Zhong[a†], Bin Yang[a†], Zhenhua zhang[b†], Xiaojuan Wang[d], Yinfeng Guo[e], Weifeng Huang[e], Qianqian Wang[e], Guodi Cai[e], Fan Xia[e], Shengning Zhou[a], Shuai Ma[a], Yichu Nie[b], Jinping Lei[e], Min Li[e, f], Peiqing Liu[e, f], Wenbin Deng[b], Yonghong Liu[c], Junfeng Wang[c]*, Fanghai Han[a]*, Junjian Wang[e, f]*) to current author list (CDDIS-21-2849RR:Lin Zhong[a†], Bin Yang[a†], Zhenhua zhang[b†], Junfeng Wang[c]*, Xiaojuan Wang[d], Yinfeng Guo[e], Weifeng Huang[e], Qianqian Wang[e], Guodi Cai[e], Fan Xia[e], Shengning Zhou[a], Shuai Ma[a], Yichu Nie[b], Jinping Lei[e], Min Li[e, f], Peiqing Liu[e, f], Wenbin Deng[b], Yonghong Liu[c], Fanghai Han[a]*, Junjian Wang[e, f]*) .

We need all the coauthors to confirm whether they agree to these changes. If you agree to these changes, please reply "I agree to these author list changes" followed by your name, otherwise, please let me know if you have any questions!

Thank you for your help!

Junjian Wang

本邮件及其附件含有发送给特定个人和用于特定目的的信息。如果您不是预期的收件人，请立即删除本邮件并通知发件人。严禁任何非预期的收件人使用、传播、分发或复制本邮件或其附件。
This email and its attachments may contain confidential information intended for a specific individual and purpose. If you are not the intended recipient, you should delete this email and notify the sender immediately. Any use, dissemination, distribution, or copying of this email or its attachments by persons other than the intended recipient(s), is strictly prohibited.

本邮件及其附件含有发送给特定个人和用于特定目的的信息。如果您不是预期的收件人，请立即删除本邮件并通知发件人。严禁任何非预期的收件人使用、传播、分发或复制本邮件或其附件。
This email and its attachments may contain confidential information intended for a specific individual and purpose. If you are not the intended recipient, you should delete this email and notify the sender immediately. Any use, dissemination, distribution, or copying of this email or its attachments by persons other than the intended recipient(s), is strictly prohibited.
