# Supplementary material for: Targeting autophagy peptidase ATG4B with a novel natural product inhibitor Azalomycin F4a for advanced gastric cancer
Source: Cell Death Dis. 2022 Feb 18;13(2):161. doi: 10.1038/s41419-022-04608-z (PMC8858318; doi:10.1038/s41419-022-04608-z)
Supplement: Supplementary file 2 — Supplemental material [file 41419_2022_4608_MOESM2_ESM.docx]

**Supplemental material**

**Targeting autophagy peptidase ATG4B with a novel natural product inhibitor Azalomycin F4a for advanced gastric cancer**

Lin Zhong^a†^, Bin Yang^a†^, Zhenhua zhang^b†^, Junfeng Wang^c^*****, Xiaojuan Wang^d^, Yinfeng Guo^e^, Weifeng Huang^e^, Qianqian Wang^e^, Guodi Cai^e^, Fan Xia^e^, Shengning Zhou^a^, Shuai Ma^a^, Yichu Nie^b^, Jinping Lei^e^, Min Li^e, f^, Peiqing Liu^e, f^, Wenbin Deng^b^, Yonghong Liu^c^, Fanghai Han^a^*****, Junjian Wang^e, f^*****

^a^ Department of Gastrointestinal Surgery, Sun Yat-sen Memorial Hospital, Sun Yat-sen University, Guangzhou, Guangdong 510120, China;

^b^ Department of Pharmaceutical Sciences (Shenzhen), Sun Yat-Sen University, Guangzhou, Guangdong 510006, China;

^c^ CAS Key Laboratory of Tropical Marine Bio-resources and Ecology/Guangdong Key Laboratory of Marine Materia Medica, South China Sea Institute of Oceanology, Chinese Academy of Sciences, Guangzhou 510301, China;

^d^ Hepatopancreatobiliary Center, Beijing Tsinghua Changgung Hospital, Tsinghua University, No.168, Litang Road, Changping District, Beijing 102218, China.;

^e^ School of Pharmaceutical Sciences, Sun Yat-sen University, Guangzhou, Guangdong, 510006, China

^f^ Guangdong Provincial Key Laboratory of New Drug Design and Evaluation, National-Local Joint Engineering Laboratory of Druggability and New Drugs Evaluation, Sun Yat-sen University, Guangzhou, Guangdong, 510006, China.

***Corresponding author**

wangjj87@mail.sysu.edu.cn (Junjian Wang)，hanfh@mail.sysu.edu.cn (Fanghai Han) or wangjunfeng@scsio.ac.cn (Junfeng Wang)

^†^ These authors made equal contributions to this work.

**
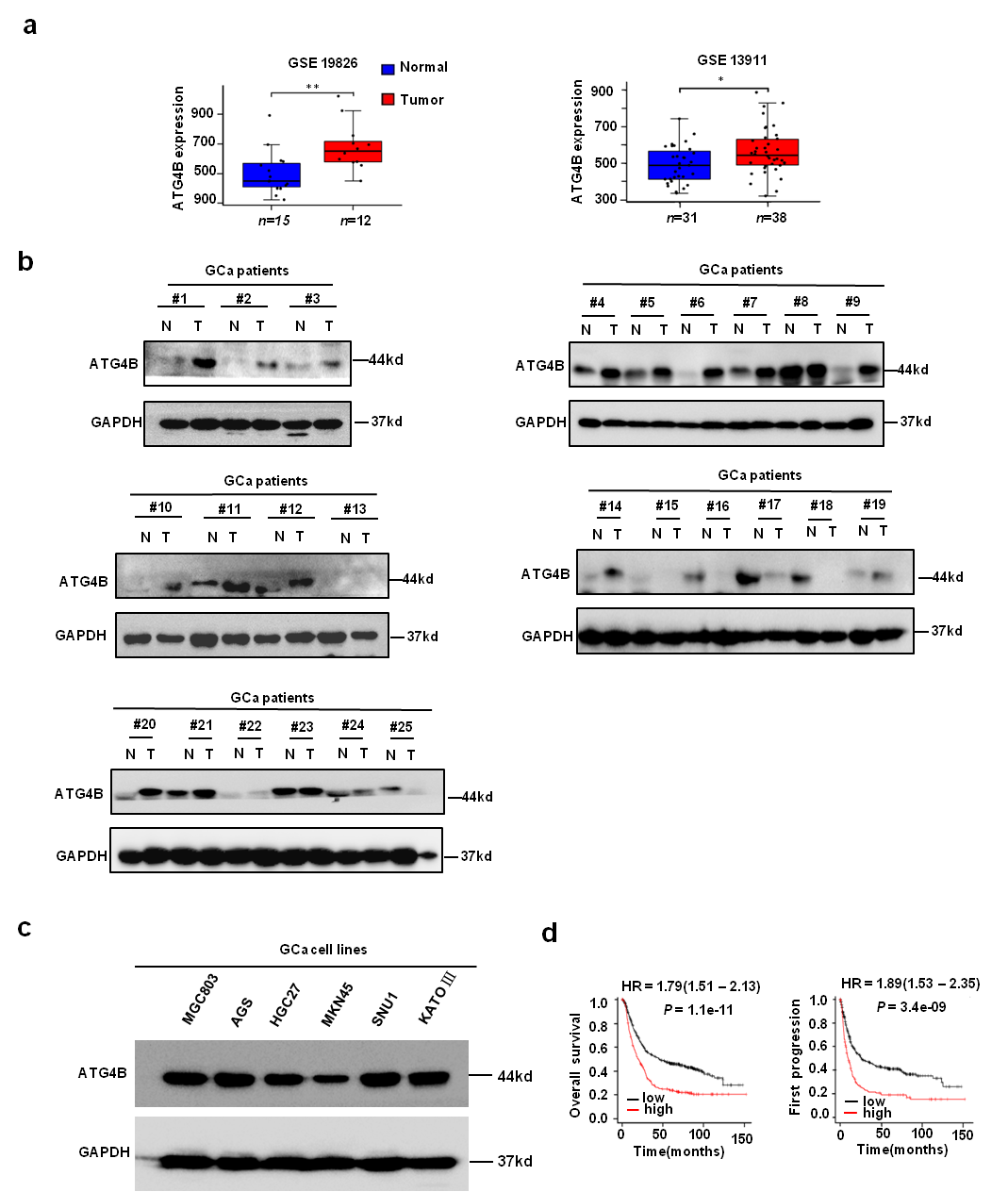
**

**
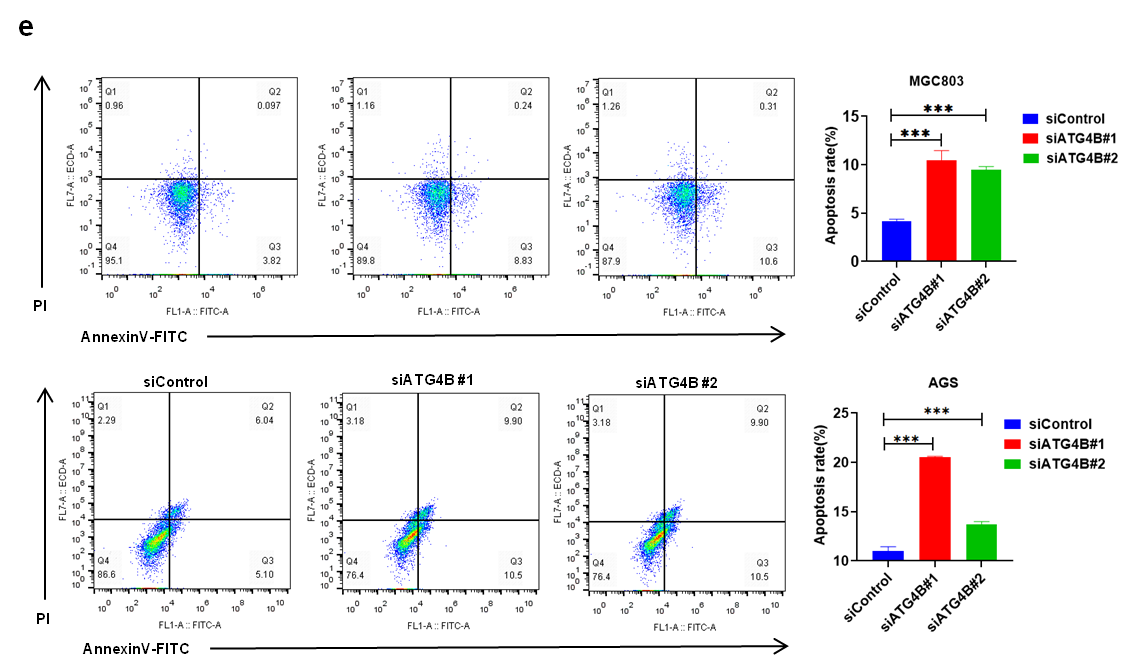
**

**Figure S1. ATG4B is highly overexpressed in GCa cells and tumors. (a).** ATG4B transcript levels from two GEO datasets were queried for association with disease status（normal and tumor). *p* value was calculated by using two-tailed Student’s *t*-test. **(b).** Expression of ATG4B in gastric tumor and paired adjacent noncancerous tissues from 25 patients was measured by WB (Note: the WB of the first six patients is same as the Fig1b in the article). **(c).** ATG4B protein levels were analyzed by immunoblotting in GCa cell lines. **(d).** Kaplan-Meier overall survival and relapse curve analysis of GCa patients, stratified by ATG4B expression (data were obtained from: https://kmplot.com/analysis/). **(e).** The apoptosis percentage of AGS /MGC803 transfected with ATG4B or control siRNA was detected by flow cytometry using AnnexinV/PI staining. Student’s t test, * *p* < 0.05, ** *p* < 0.001，****p* < 0.001.


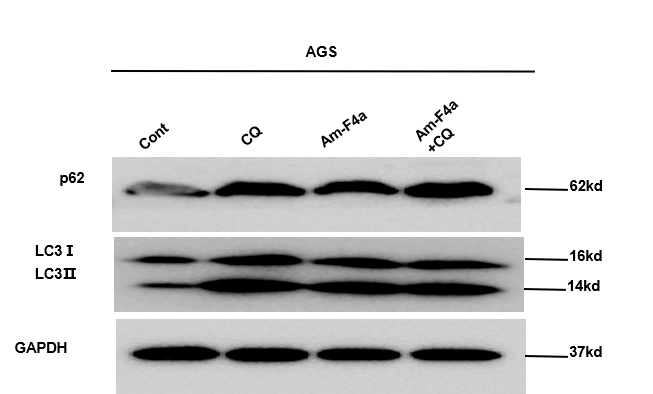


**Figure S2. CQ didn't further enhance LC3-Ⅱ accumulation induced by Am-F4a.** AGS cells treated with Am-F4a (5μM) for 48 hours, then CQ (40μM) was added into Am-F4a-treated cells and control cells for 4h. LC3 and P62/SQSTM1 were analyzed by immunoblot. Representative blots, *n* = 3.


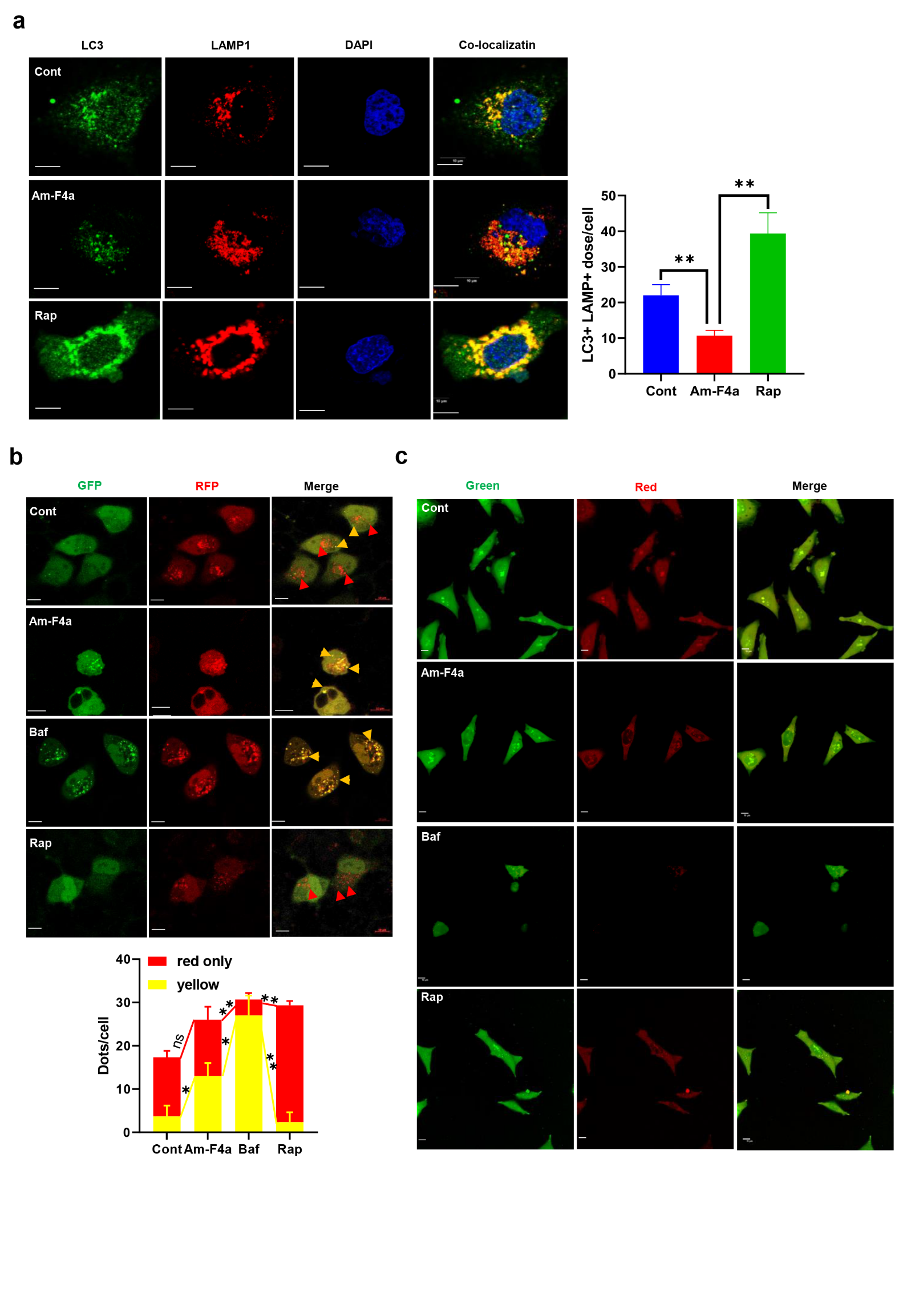


**Figure S3. Am-F4a suppresses autophagy flux in Gca cells.**  **(a) .** MGC803 cells were treated with Am-F4a (10 μM) and Rap (1 μM) for 6 h. The colocalization of LC3 and LAMP1 puncta was examined and quantified (scale bar, 10 μm). **(b).** MGC803 cells expressing GFP-RFP-LC3 were treated with Am-F4a (10 μM) 、Rap (1 μM) and Baf（0.5 μM）for 6 h. The colocalization of GFP and RFP puncta was examined and quantified. Red arrows indicate GFP- or LC3-positive structure, yellow arrows indicate the colocalization of GFP and RFP. Fluorescence images of cells were recorded with fixation (scale bar, 10 μm). **(c) .** Acridine Orange (AO) can indicate normal autolysosome structures of cells. MGC803 cells were treated with Am-F4a (10 μM), Rap (1 μM), Baf (0.5 μM) for 4 h, followed by AO (0.5 μg/mL) for 30 min. Fluorescence images of live cells were recorded without fixation (scale bar, 10 μm). Data are shown as mean ± SD, *n* = 3, Student’s t test, **p* < 0.05, ***p* < 0.01.


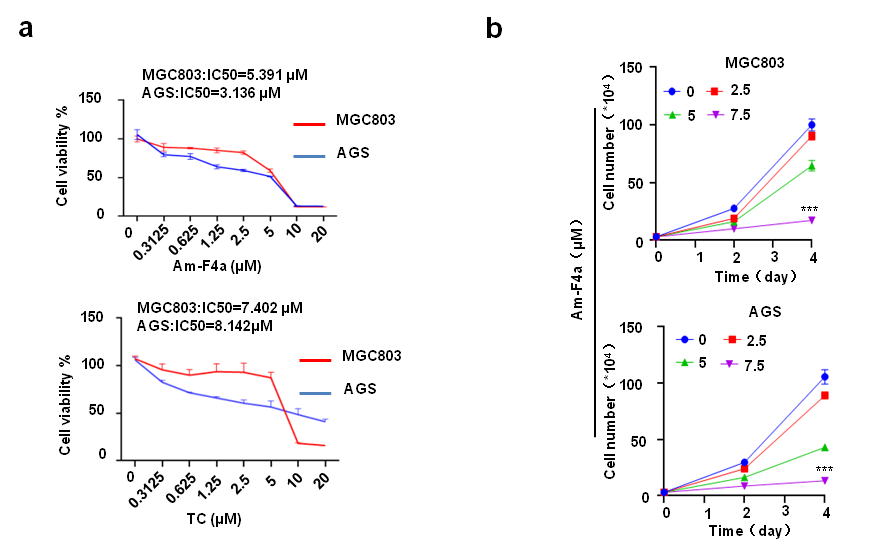


**Figure S4.** **The anti-tumor effect of Am-F4a was showed in a time-dependent manner. (a).** Cell viability was evaluated by CCK-8 assay of MGC803 and AGS cells treated with Am-F4a and Tioconazole (TC). IC50 was counted. **(b).** MGC803 and AGS cells were treated with Am-F4a in indicated concentration. After indicated time points, viable cells were counted. Data shown are mean ± SD. Student’s *t*-test. ****p* < 0.001., *n* = 3.


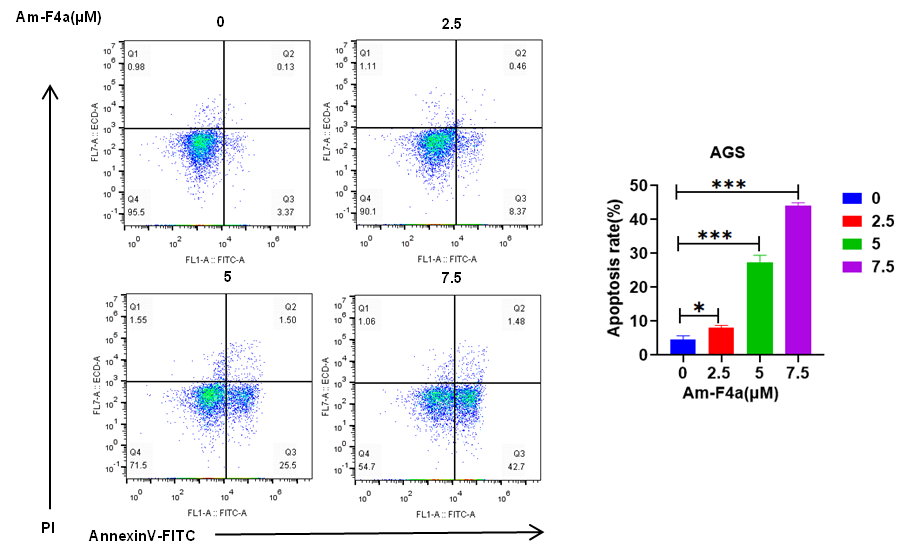


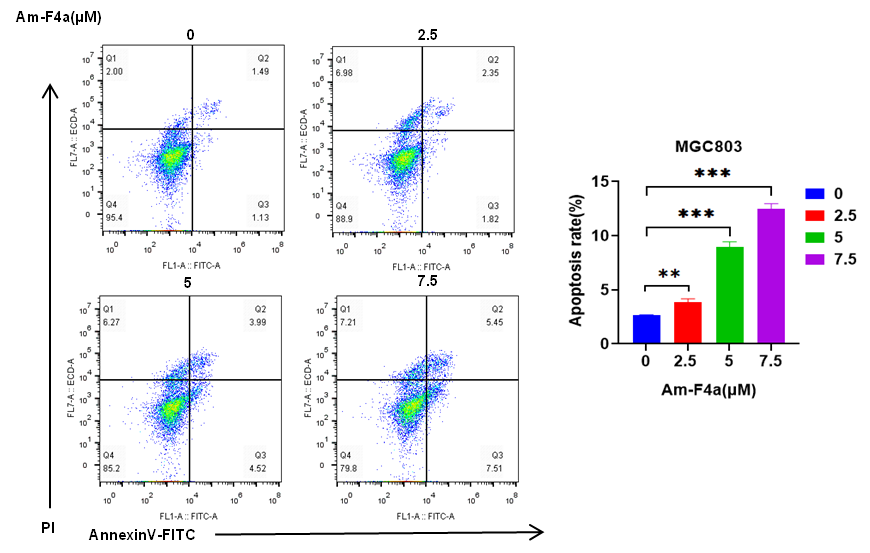


**Figure S5. ATG4B was associated with the apoptosis of GCa cell.** The apoptosis percentage of AGS /MGC803 treated with Am-F4a in different concentration was detected by AnnexinV/PI. Student’s t test, **p*<0.05, ***p*<0.01, ****p* < 0.001.

**
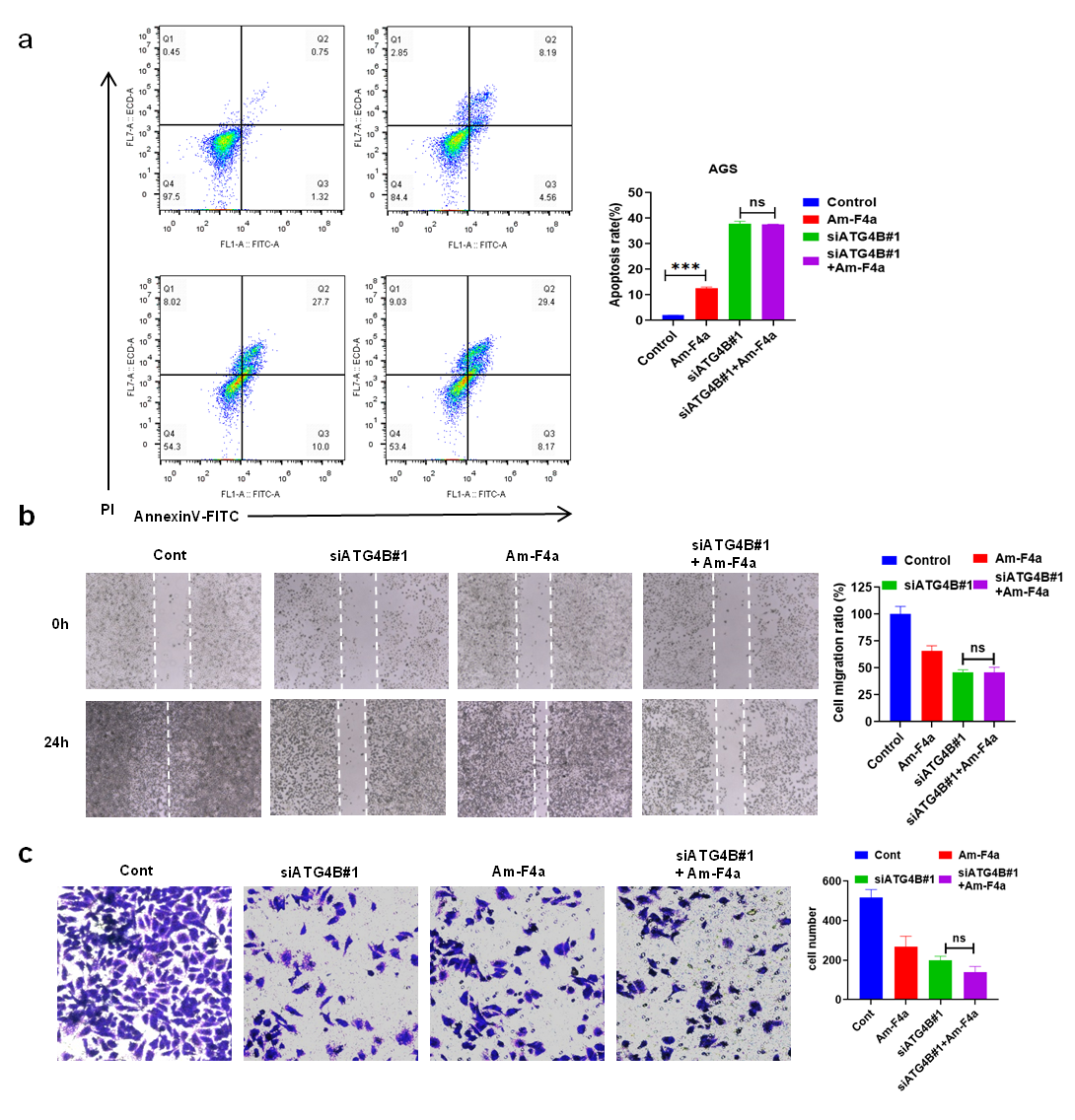
**

**Figure S6. Effect of Am-F4a on GCa cell apoptosis, migration and invasion was obviously attenuated in ATG4B knockdown cells. (a).** AGS cells were transfected with ATG4B or control siRNA for 48 hours and then treated with Am-F4a at for another 24 hours. The apoptosis percentage was detected by AnnexinV/PI. **（b）.** The migration of GCa cells were determined using would healing assay. **（c）.** The invasion ability of AGS cells were evaluated by transwell assay. Student’s t test, ****p* < 0.001.

**
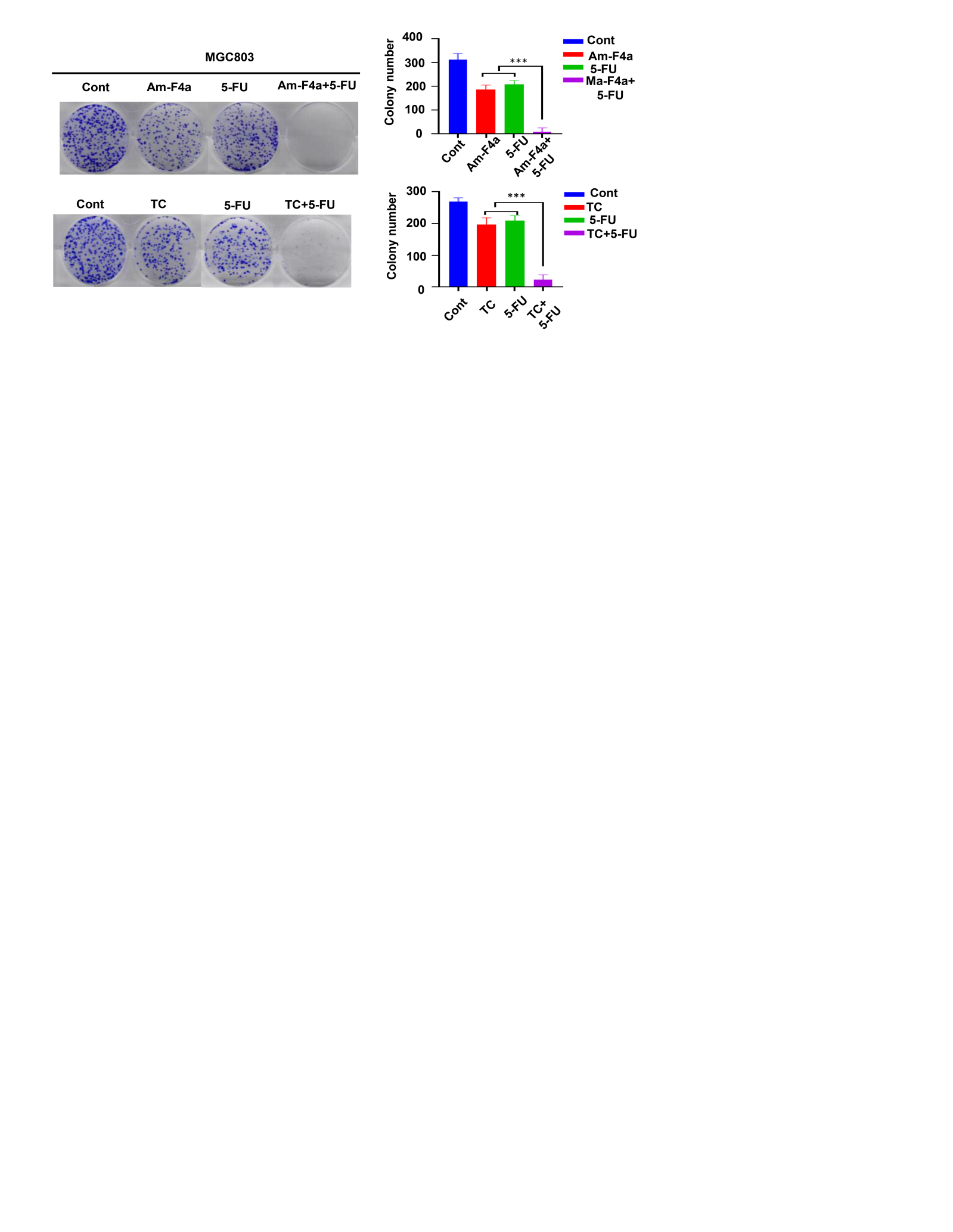
**

**Figure S7. ATG4B inhibitors improved anti-GCa efficacy of 5-FU.** Colony formation of MGC803 and AGS cells treated with Am-F4a at indicated concentration. Representative images were shown, and colonies were counted. Data shown above are mean ± SD, *n* = 3, Student’s t test, ****p* < 0.001.

**
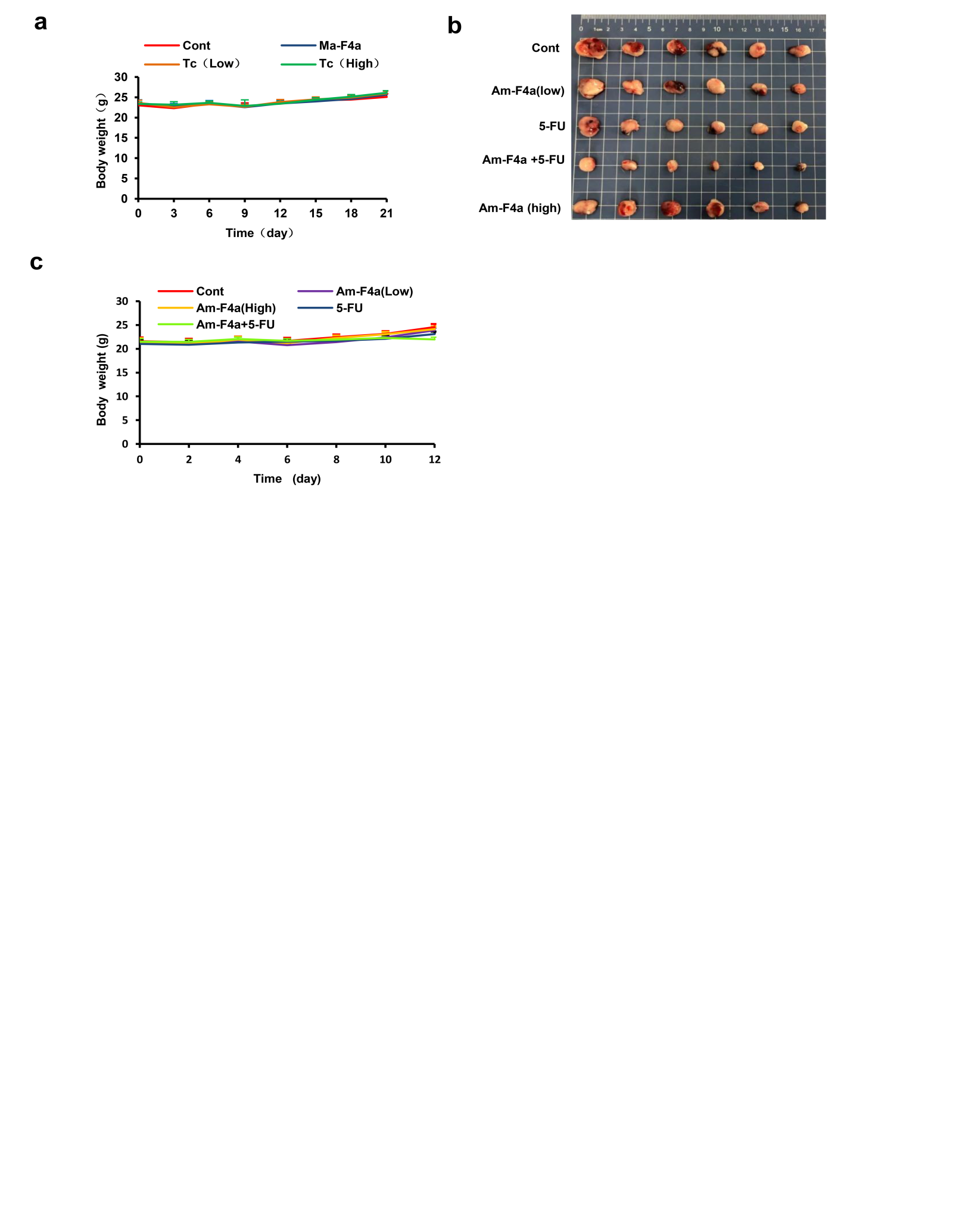
**

**Figure S8. Am-F4a inhibited GCa tumor growth and improved 5-FU treatment *in vivo*. (a).** The weight of four group mice has no obvious change. **(b).** Representative tumor image of PDX xenografts, at the end time point was captured. **(c).** The weight of five group mice has no obvious change.


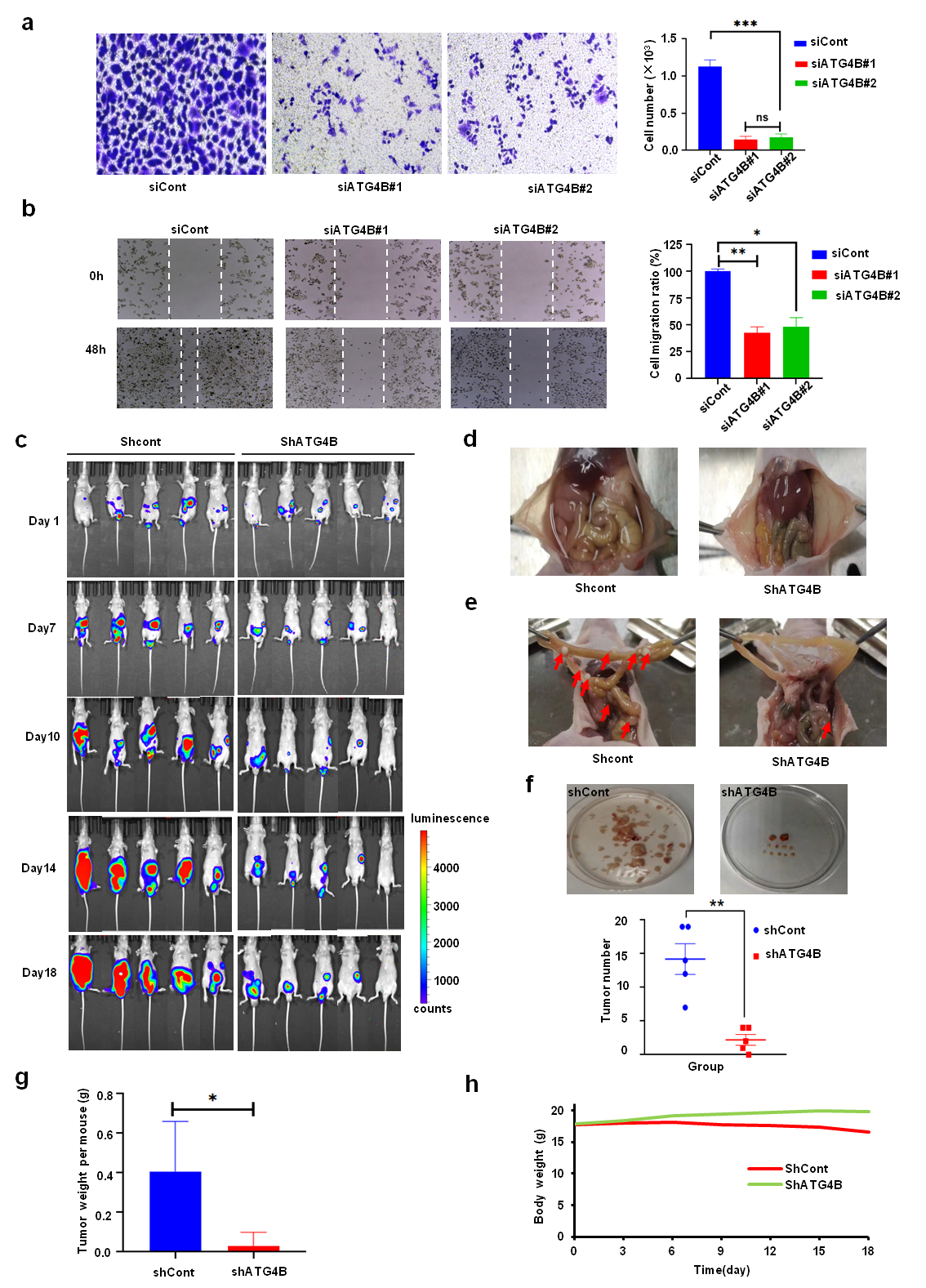


**Figure S9. ATG4B was associated with GCa tumor metastasis. （a）.** AGS cells were transfected with ATG4B or control siRNA for 48 hours, The invasion ability of AGS cells were evaluated by Transwell assay. **（b）.** The migration of AGS cells were determined using would Healing assay. **（c-e）.** MKN-45 cells expressing pLenti-Firefly Luciferase-EGFP were infected with lentiviruses expressing control or shATG4B shRNA before injecting into the peritoneal cavity of nude mice, monitored by bioluminescence. (**d-e).** Representative image of the peritoneal metastasis of MKN-45 cells in mice at the end of study， malignant ascites persent in shCont group（**d**），red arrows indicate tumor**.**  (**f - g).** Representative tumor image and tumor weight per mouse at the end time point was captured. **(h)**. Body weight was monitored. Data shown above was calculated using two-tailed Student’s *t*-test, **p*<0.05, ***p*<0.01, ****p* < 0.001.


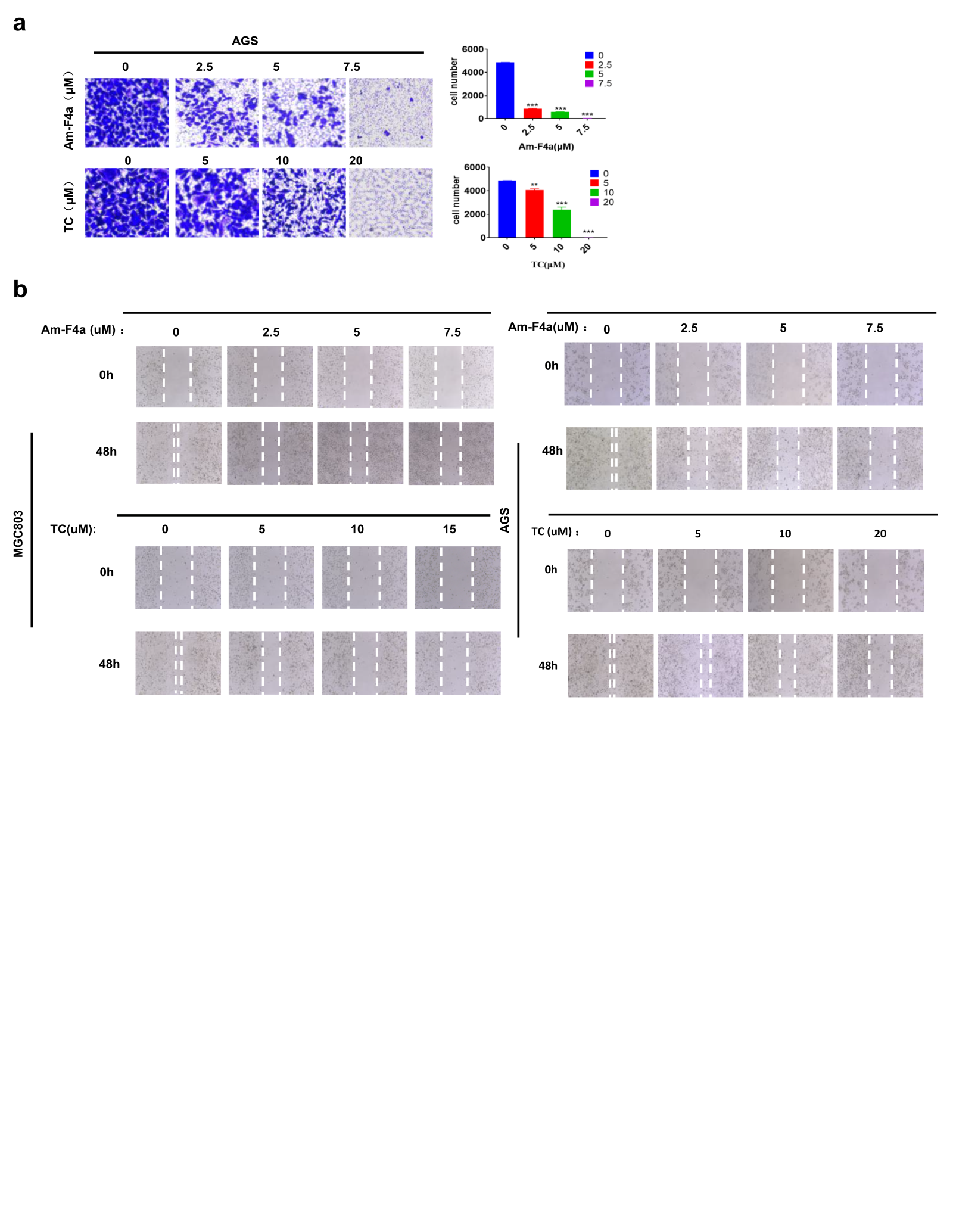


**
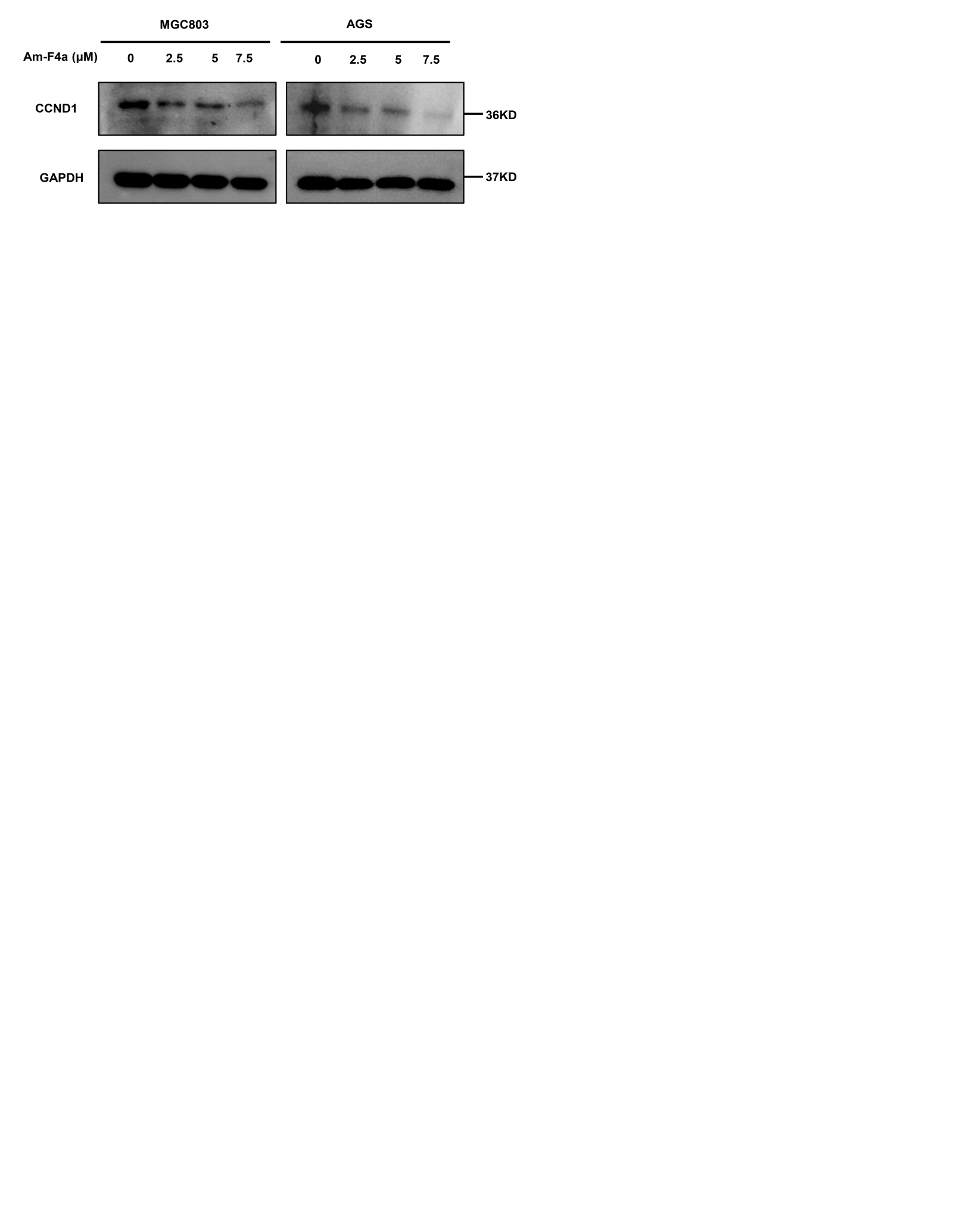
Figure S10. ATG4B inhibitor inhibited GC tumor metastasis. (a).** The invasion ability of AGS cells treated with Am-F4a, TC or vehicle at indicated concentration were evaluated by transwell assay. Data shown mean ± SD, *n* = 3, ****p* < 0.001. **(b).** The migration of GCa cells were determined using would healing assay.

**Figure S11. ATG4B inhibition increased CCND1 expression in GCa cells.** Immunoblotting analysis of CCDN1 in MGC803 and AGS cells treated with Am-F4a for 48 hours. Representative blots, *n* = 3.


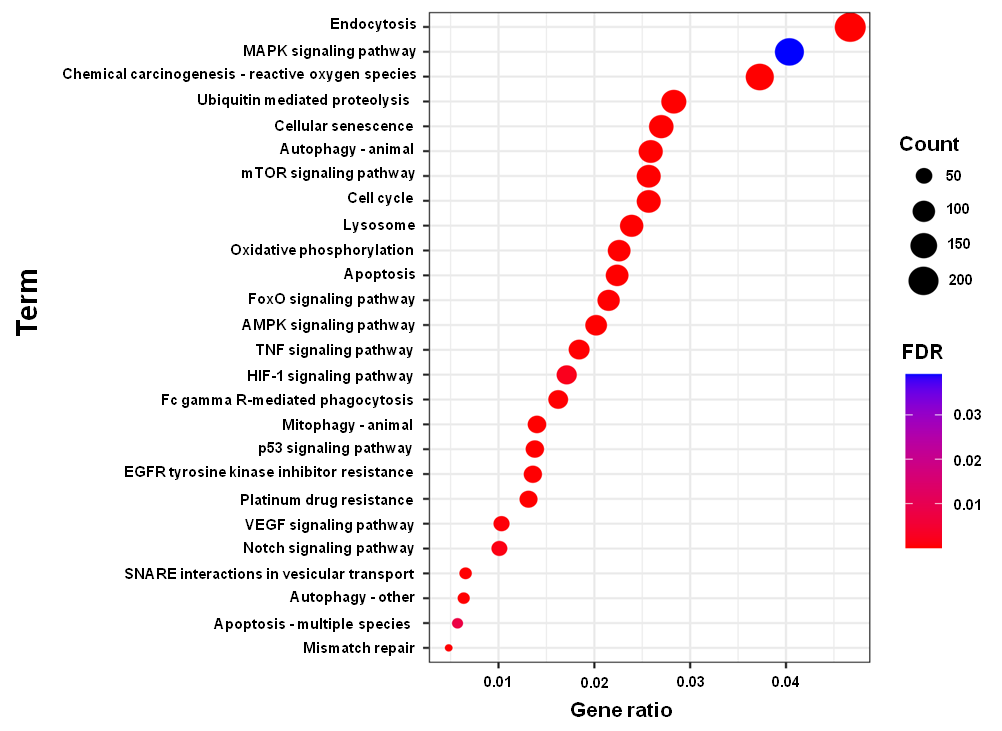


**Figure S12. The potential mechanism of ATG4B in GCa cells.** The bubble plot of the pathways of KEGG enrichment analysis. The KEGG pathway plot was displayed with gene counts, gene ratio, -log10*p* value.
