# Supplementary figures and images for: Targeting autophagy peptidase ATG4B with a novel natural product inhibitor Azalomycin F4a for advanced gastric cancer
Source: Cell Death Dis. 2022 Feb 18;13(2):161. doi: 10.1038/s41419-022-04608-z (PMC8858318; doi:10.1038/s41419-022-04608-z)

Fig. 1b

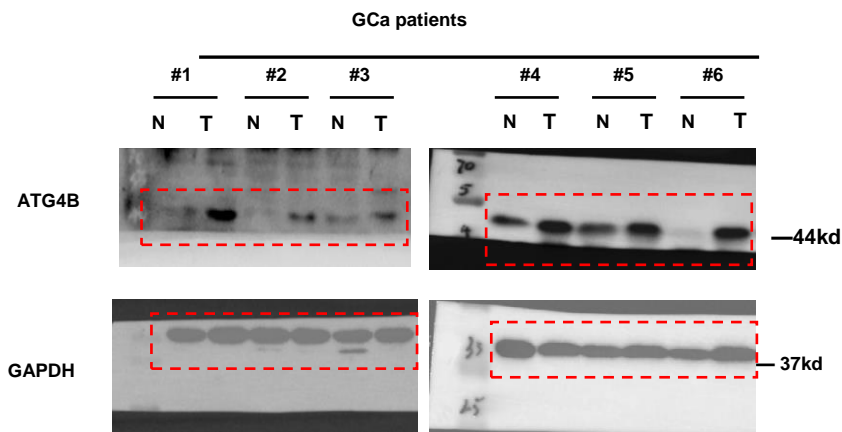

Fig. 1f

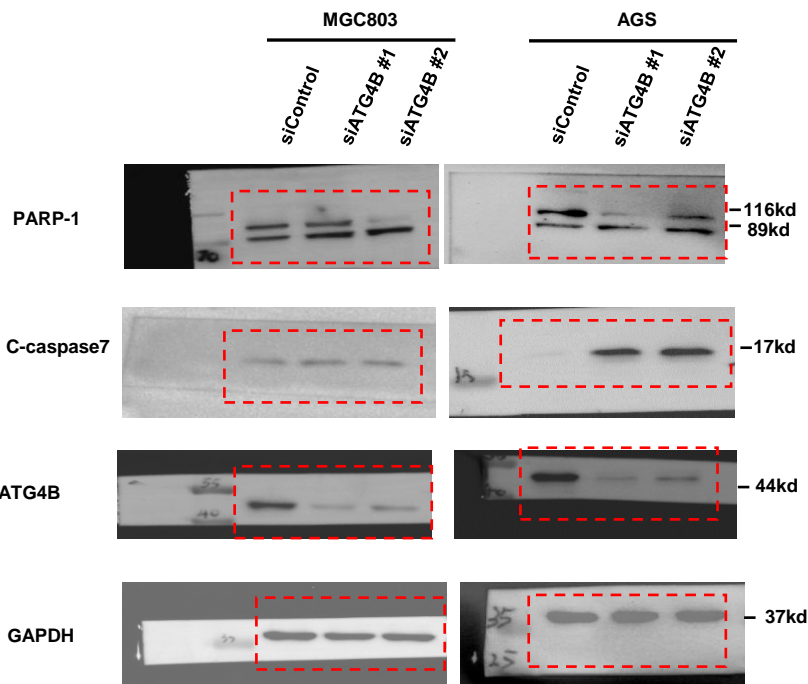

**Fig. 2b**

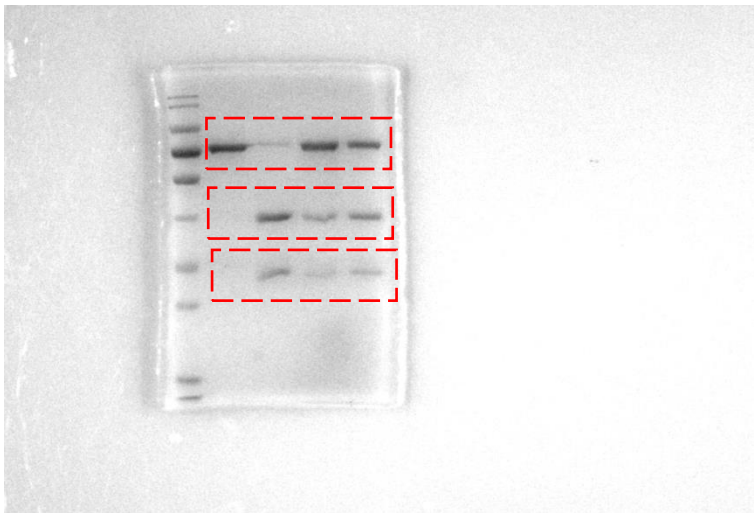

**Fig. 3a**

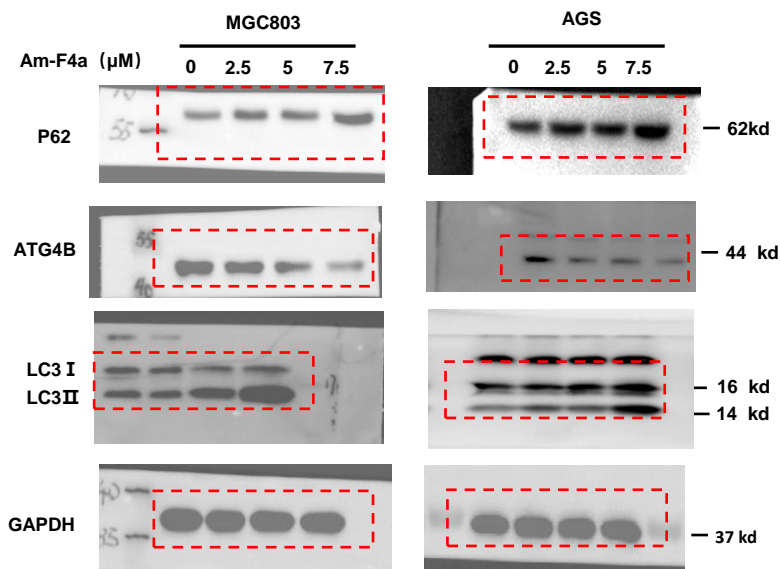

Fig. 4c

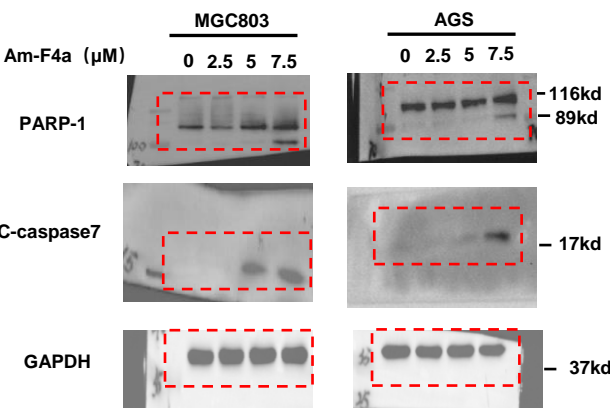

Fig. 5d

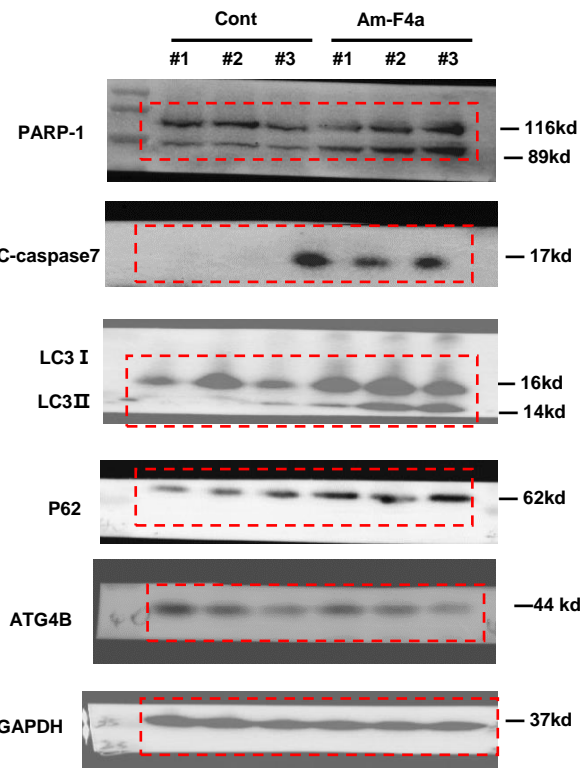

**Fig. 6c**

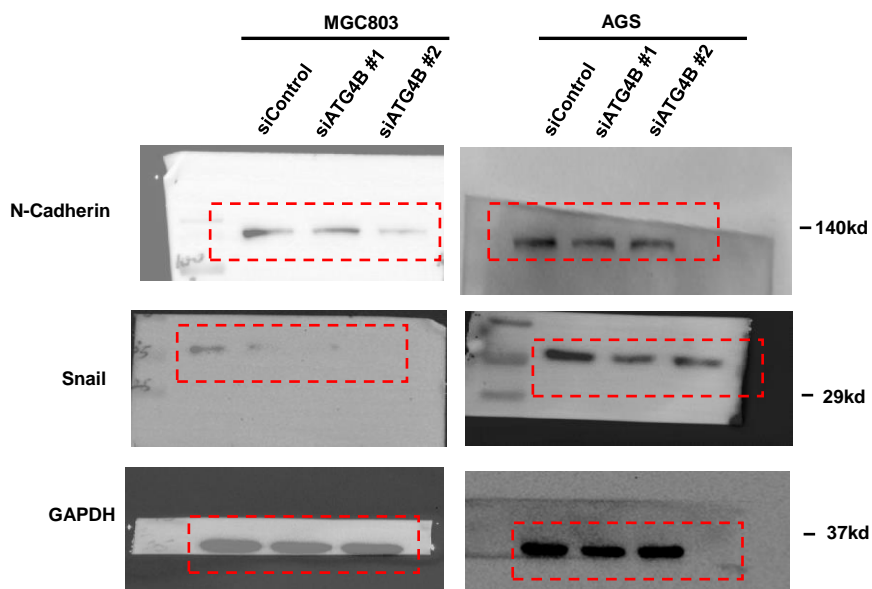

**Fig. 6d**

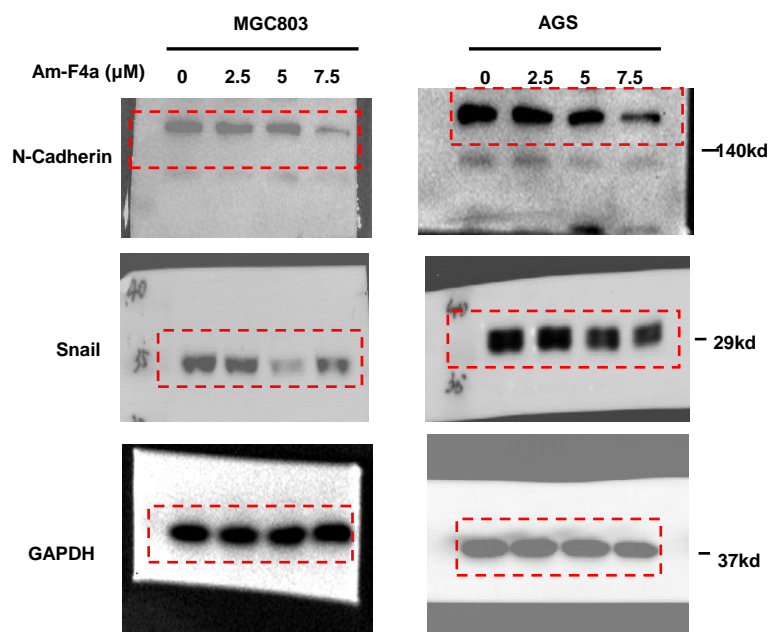

Fig.S1b

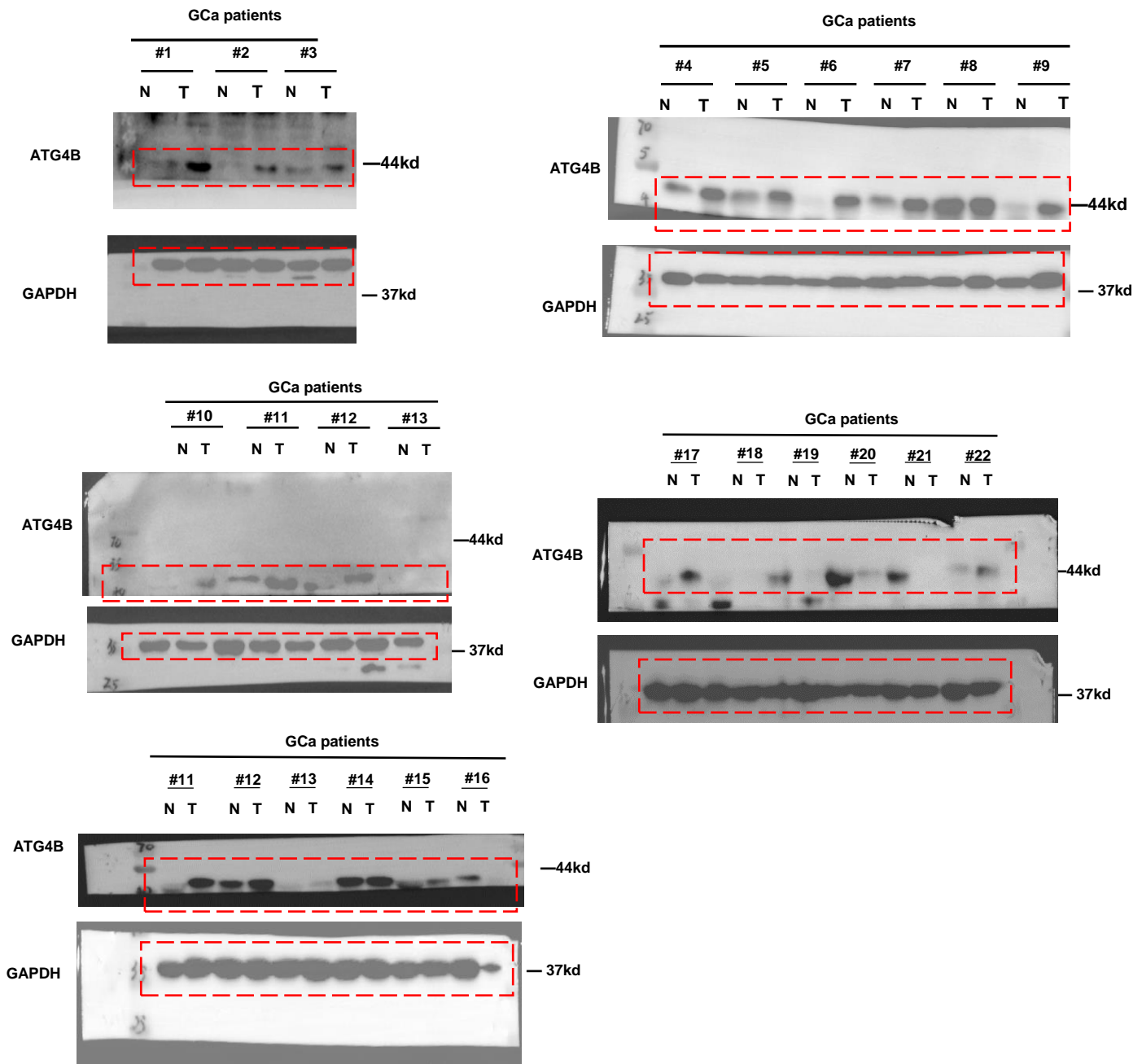

Fig. S1 c

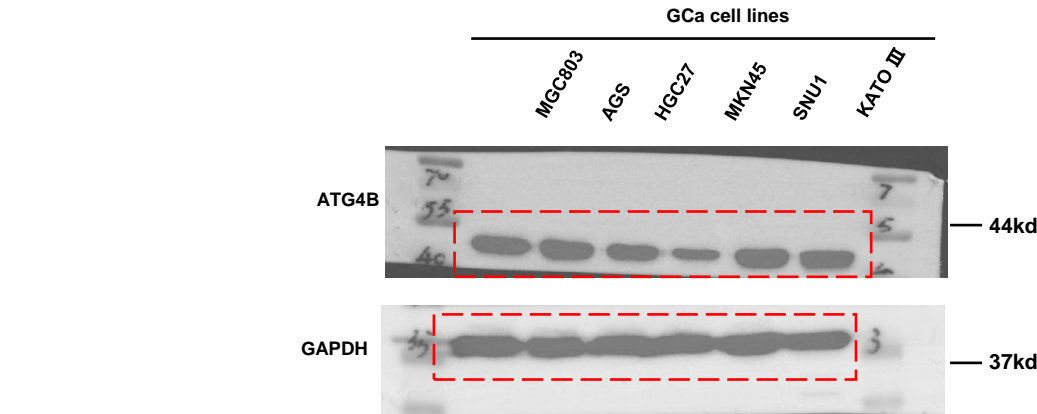

Fig.S2

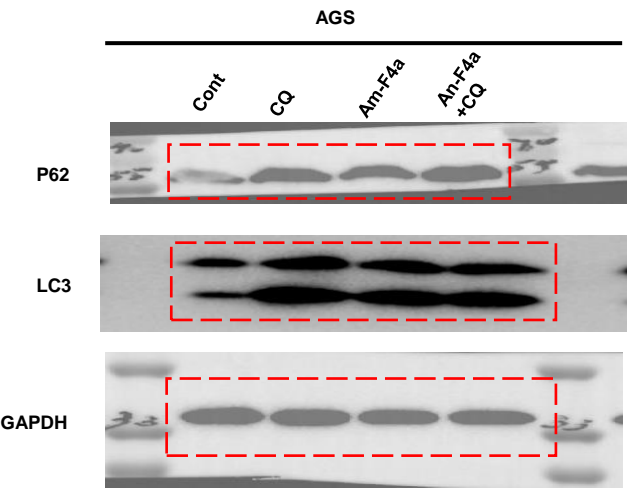

Fig.S11

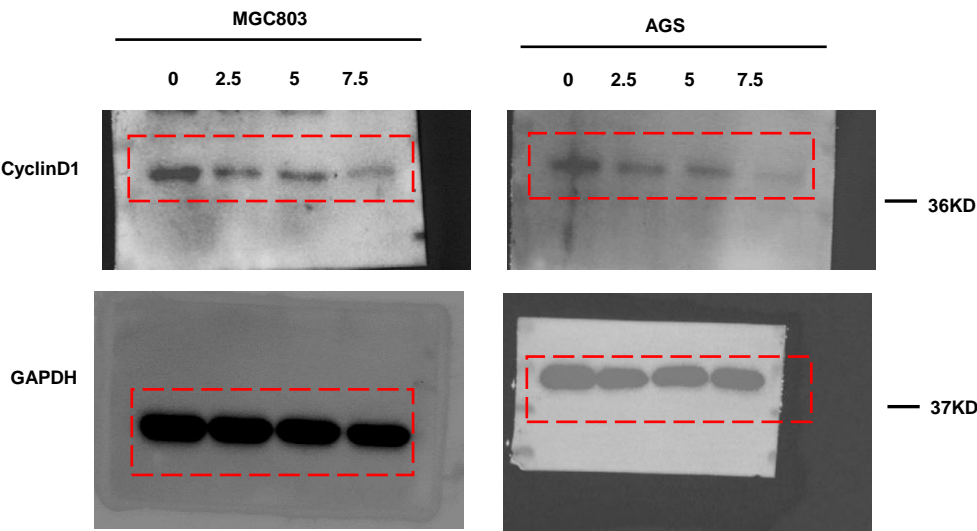

Supplement: Supplementary file 3 — Original western blots [file 41419_2022_4608_MOESM3_ESM.pdf]
